# Spatiotemporal and direct capturing global substrates of lysine-modifying enzymes in living cells

**DOI:** 10.1038/s41467-024-45765-3

**Published:** 2024-02-17

**Authors:** Hao Hu, Wei Hu, An-Di Guo, Linhui Zhai, Song Ma, Hui-Jun Nie, Bin-Shan Zhou, Tianxian Liu, Xinglong Jia, Xing Liu, Xuebiao Yao, Minjia Tan, Xiao-Hua Chen

**Affiliations:** 1 State Key Laboratory of Drug Research, Shanghai Institute of Materia Medica, Chinese Academy of Sciences, Shanghai, 201203 China; 2 Translational Research Institute of Brain and Brain-Like Intelligence, Shanghai Fourth People’s Hospital, School of Medicine, Tongji University, Shanghai, 200434 China; 3 MOE Key Laboratory for Cellular Dynamics and Hefei National Center for Physical Sciences at the Microscale, University of Science and Technology of China, Hefei, 230026 China; 4 University of Chinese Academy of Sciences, Beijing, 100049 China; 5 Zhongshan Institute for Drug Discovery, Shanghai Institute of Materia Medica, Chinese Academy of Sciences, Zhongshan Guangdong, 528400 China; 6 School of Pharmaceutical Science and Technology, Hangzhou Institute for Advanced Study, University of Chinese Academy of Sciences, Hangzhou, 310024 China

**Keywords:** Chemical tools, Acetylation, Transferases, Acetyltransferases, Protein-protein interaction networks

## Abstract

Protein-modifying enzymes regulate the dynamics of myriad post-translational modification (PTM) substrates. Precise characterization of enzyme-substrate associations is essential for the molecular basis of cellular function and phenotype. Methods for direct capturing global substrates of protein-modifying enzymes in living cells are with many challenges, and yet largely unexplored. Here, we report a strategy to directly capture substrates of lysine-modifying enzymes via PTM-acceptor residue crosslinking in living cells, enabling global profiling of substrates of PTM-enzymes and validation of PTM-sites in a straightforward manner. By integrating enzymatic PTM-mechanisms, and genetically encoding residue-selective photo-crosslinker into PTM-enzymes, our strategy expands the substrate profiles of both bacterial and mammalian lysine acylation enzymes, including bacterial lysine acylases PatZ, YiaC, LplA, TmcA, and YjaB, as well as mammalian acyltransferases GCN5 and Tip60, leading to discovery of distinct yet functionally important substrates and acylation sites. The concept of direct capturing substrates of PTM-enzymes via residue crosslinking may extend to the other types of amino acid residues beyond lysine, which has the potential to facilitate the investigation of diverse types of PTMs and substrate-enzyme interactive proteomics.

## Abstract (web summary)

Here the authors report a strategy to directly capture substrates of lysine-modifying enzymes via post-translational modification (PTM)-acceptor residue crosslinking in living cells, enabling global profiling of substrates of PTM-enzymes and validation of PTM-sites in a straightforward manner.

## Introduction

**Fig. 1:**
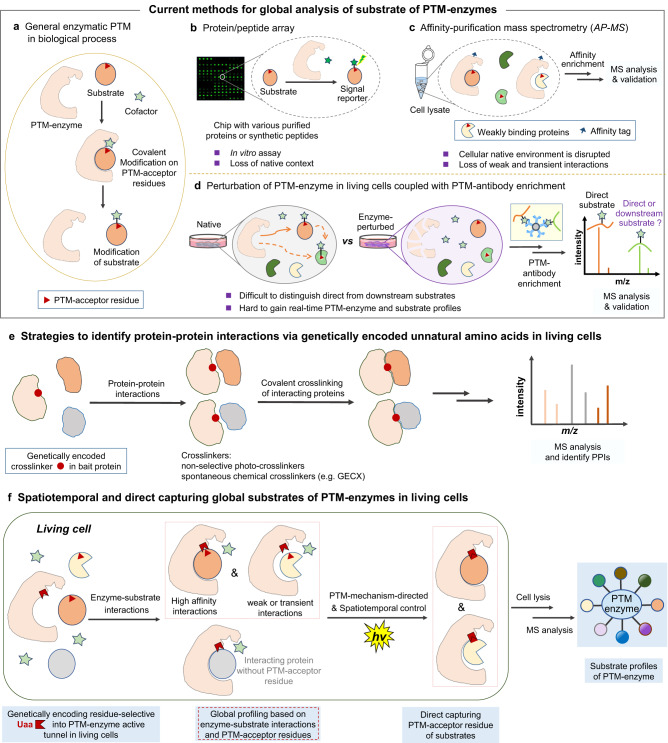
The development of post-translational modification (PTM)-mechanism-directed covalent capturing strategy via PTM-acceptor residue in living cells. **a** General enzymatic PTM in biological processes. **b** Protein or peptide array for validation of substrate modification via PTM-acceptor residue in the presence of enzymes in vitro. **c** Affinity purification coupled with MS analysis for validation of substrate-enzyme interactions. **d** Pharmacological or genetic perturbation of cellular enzyme activity coupled with PTM-antibody enrichment-based proteomics **e** Strategies to identify protein-protein interactions (PPIs) via genetically encoded non-selective photo-crosslinkers or spontaneous chemical crosslinkers (e.g., GECX) in living cells. **f** Spatiotemporal and direct capturing global substrates of post-translational modification enzyme (PTM-enzyme) via PTM-acceptor residue of substrates, through genetically encoding residue-selective unnatural amino acid (Uaa) photo-crosslinker in living cells, offering substrate profiles of PTM-enzyme from MS analysis.

Post-translational modifications (PTMs) are covalent chemical alterations of proteins after ribosome biosynthesis, which are typically mediated by enzymes (Fig. 1a). Currently, there are more than 500 types of PTMs that extensively modulate protein functions^1^. Many PTMs, such as acylation, phosphorylation, and ubiquitination, are reversibly controlled by protein-modifying enzymes in a dynamic manner at corresponding localization and time, orchestrating complex cellular processes^2^. Aberrant PTM states caused by dysfunction of regulatory enzymes are broadly involved in the development of various complex diseases^3^. Therefore, precise characterization of enzyme-substrate associations is essential for functional annotation of PTMs under physiological and pathological conditions^4^. Indeed, the PTM substrate-centric strategies with the aim to identify corresponding protein-modifying enzymes, such as chemical proteomics based on PTM-decorated substrate surrogates^5,6^, protein microarray (or peptide libraries)^7^, and tethered catalytic complex^8^, have successfully dissected various enzyme-substrate associations. On the other hand, enzyme-centric methods enable deciphering substrate profiles of PTM-enzymes to determine enzymatic activities and biological functions^4^. Since protein-modifying enzymes usually control the dynamics for myriad PTM substrates, global profiling of substrates could dissect the complicated and elusive enzyme-substrate interactions, and further illuminate the landscape of complex regulatory networks^2,4^. Thus, the development of such enzyme-centric methods is particularly significant and highly desired.

Current enzyme-centric methods for substrate profiling could be categorized into those conducted in vitro and within cells. In vitro methods usually screen against protein or peptide microarrays (Fig. 1b) in an unbiased and high-throughput manner^7,9^, but the cellular native context is eliminated. Although methods (e.g., affinity purification mass spectrometry, Fig. 1c)^2,4,10^ performed on cell lysate retain certain complex biological environments, the cellular native compartmentalization is disrupted, and they still suffer from the loss of weak and transient interactions, thus, resulting in reduced spatial resolution and accuracy^2,10^. In contrast, approaches conducted in cells allow dissection of the enzyme-substrate relationships in a native milieu. Genetic perturbation of cellular enzyme activity coupled with PTM-antibody enrichment-based proteomics is the most widely used strategy (Fig. 1d)^11,12^. However, such genetic perturbation not only impacts the direct substrates of the enzyme but also those downstream/bypassing events. Indeed, protein PTMs often modulate protein (e.g., enzymes) activity which could, in turn, reshape the PTM states of downstream proteins, such as phosphorylation cascade^13^. An obvious drawback of this approach is difficult to distinguish the direct substrates of the enzyme from those indirect ones^12^. Moreover, what the genetic perturbation-based PTM-proteomic profiling experiment measures is the change of PTM level accumulated over a time course (e.g., from genetic perturbation of PTM-enzyme to cell harvest for PTM-profiling) instead of an instantaneous “snapshot” of substrate-enzyme interaction profiles at given time points. Thus, it is almost impossible to identify the real-time interacting substrates for the PTM-enzyme. Therefore, the genetic perturbation approach makes it difficult to distinguish the direct substrates from the indirectly altered PTM-proteins, and is not fit for the real-time PTM-enzyme and substrate interaction study.

In principle, higher decoding precision could be achieved by methods that directly capture enzyme-substrate interactions in living cells, conferring irreversibly covalent enzyme-substrate complexes for subsequent stringent purification, enrichment, and proteomic analysis^14–16^. In fact, within highly dynamic and complicated native cellular contexts, a major challenge in developing a direct substrate-capturing strategy for post-translational modification enzymes is to capture bona fide substrates, including weak and transient interacting substrates, but not the non-substrate binding proteins. Indeed, the genetic code expansion technology to site-specifically incorporate unnatural amino acids (Uaas) renders minimal structural perturbation on bait proteins (Fig. 1e)^17–22^, enabling the formation of covalent linkages to irreversibly crosslink protein complexes^23–34^. Recently, an important advancement of the genetically encoded chemical crosslinking (GECX) method by Wang’s group, is an elegant strategy and exhibited excellent abilities to covalently capture the protein-protein interactions in living cells^27,32–34^, while GECX method as yet is mainly focused on the investigation of the interacting proteins (e.g., interacting partners of non-enzyme or enzyme proteins). Thus, the potential and capability of PTM mechanism-guided direct capturing global substrates of post-translational modification enzymes via PTM-acceptor residues presents an exciting prospect yet largely unexplored.

Herein, we present a method to directly capture substrates of lysine-modifying enzymes via PTM-acceptor residue crosslinking chemistry in living cells, enabling global profiling of substrates of PTM-enzymes and their modification sites in a straightforward manner (Fig. 1f). This method covalently captures PTM-enzyme and interacting substrates in spatiotemporal resolution, with high sensitivity and accuracy. Harnessing these advantages, our developed strategy expands the substrate profiles of lysine acylation enzymes, such as the bacterial lysine acylases PatZ, YiaC, LplA, TmcA, YjaB, and the mammalian acyltransferases GCN5 and Tip60, leading to discovery of more potential substrates and modification sites with biological significance. Notably, using this approach, we provide advanced insights into the acetylation on K131 of AcrA involved in the AcrA-TolC multidrug efflux pump assembly and the potential regulatory role in antibiotic resistance. Our work demonstrates that direct capture and validation of substrates of lysine-modifying enzymes via specific PTM-acceptor residue crosslinking chemistry in living cells (Fig. 1f) is a feasible and innovative paradigm for even more types of PTM research.

## Results

### Design of PTM mechanism-directed substrate capturing strategy

**Fig. 2:**
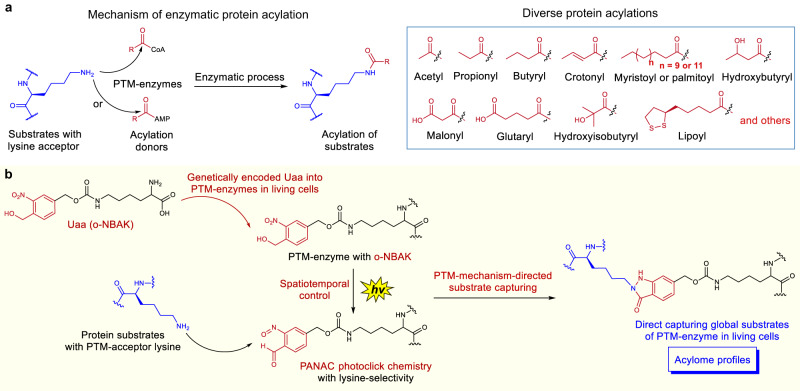
PTM mechanism-directed covalent capturing of substrates. **a** Mechanism of enzymatic protein acylation and diverse protein acylations. **b** Direct capturing substrates with PTM-acceptor lysine via spatiotemporal lysine-selective PANAC photoclick chemistry. CoA coenzyme A, AMP adenosine monophosphate.

In general, the enzymatic PTMs are covalent alterations of the acceptor residue of substrates, which are carried out via enzyme-substrate complex formation and catalytic activities of the specific functional sites of the enzyme (Figs. 1a, 2a). Inspired by the widespread success of crosslinking chemistry for capturing protein and interacting partner (or ligand) interactions, as well as exemplified by the power of GECX method to capture protein-protein interactions via diverse residue-reactivities in living cells^23–34^, we recently questioned whether it might be possible to combine the mechanism-based covalent process of a PTM with crosslinking chemistry, with an aim to directly capture enzyme-substrate interactions in living cells. More specifically, we reasoned that a genetically encoded residue-selective crosslinker in the active site tunnel of PTM enzyme should crosslink with the PTM-acceptor residues of the substrate via stable covalent linkage formation (Fig. 1f). Thus, the crosslinking would allow direct capturing global substrates of the subjected protein-modifying enzyme in living cells. However, because of the great complexity and dynamic nature of PTMs in living systems, several challenges are associated with the development of methods to directly capture substrates of PTM-enzymes. Most notably, it is elusive and difficult to discriminate the direct substrates from non-substrate binding proteins, due to the complexity of the multiprotein interacting networks^2,27^. In addition, the modifications on substrate proteins are dynamic and reversible, thus, a temporal resolution of capturing enzyme–substrate interactions at a certain stage is indispensable. Furthermore, as enzyme-substrate interactions are weak and transient in many cases, capturing these unstable and low-stoichiometry enzyme-substrate complexes requires high crosslinking efficiency from PTM-enzymes with PTM-acceptor residues of substrates^10,27^.

To fulfill these stringent demands, we reasoned that several characteristics are required for a generalizable direct capturing strategy: (i) a crosslinker with rigorous selectivity for the PTM-acceptor residue and located in the enzyme catalytic site tunnel to enable chemical (i.e., PTM-acceptor residue) and spatial dual-specificity in crosslinking, thus rendering high accuracy; (ii) temporal-control and fast-on activation of crosslinking that permits time-resolved dissection of the substrate interactome; (iii) relatively high reactivity and long reaction window of the activated crosslinker, allowing to capture sufficient amount of weak and transient enzyme-substrate complexes to satisfy the detection limit of mass spectrometry (MS) analysis; (iv) global substrate profiling criteria based on identification of captured proteins via crosslinking mass spectrometry (XL-MS) with stringent data filtration criteria built on properly designed control to further rule out non-specific background signals, which would help in identification accuracy.

As a proof-of-concept and general entry point, we herein showcase our strategy to directly capture the global substrates of several lysine-modifying enzymes. Lysine residues of proteins are among the most frequently modified amino acids with more than 40 discrete types of modification, such as acetylation, diverse short-chain acylations, lipidations, lipoylation, ubiquitination, and so on (Fig. 2a)^1^. These lysine acylations regulate numerous protein functions (e.g., stability, enzymatic activity, cellular translocation, interactions, etc.) and almost are involved in many biological processes^2,4,35^. In addition, several studies showed that lysine-modifying enzymes have promiscuous acylation activity other than certain specific modification^36,37^. More importantly, short-chain acylations of bacterial virulence regulators can modulate host immunity and susceptibility to infection^38,39^. However, the substrate profiles of acylation enzymes have been elusive^36,40^. Thus, connecting lysine-modifying enzymes to their substrates (acylome) is crucial for understanding their regulatory mechanisms and functions^41,42^.

In this context, the initial step of our study was to identify a genetically encoded crosslinker that was suitable for directly capturing acylation substrates of lysine-modifying enzymes. We reasoned that the advantages of the light-induced primary amines and o-nitrobenzyl alcohols cyclization (PANAC photoclick) are inherently linked to the direct capturing chemistry (Fig. 2b)^43^. The photo-generated aryl-nitroso intermediates feature reliable chemo-selectivity with primary amines, high reactivity, good biocompatibility under physiological conditions, and a long half-life (with hours in buffer)^43^. Based on these insights into the PANAC photoclick chemistry, we employed the o-nitrobenzyl alcohol-derived lysine (o-NBAK), an unnatural amino acid (Uaa) as a photo-crosslinker, which is able to selectively and efficiently react with lysine upon light-activation^44,45^. Thus, genetically incorporating o-NBAK in the (or near) catalytic pocket of enzymes would capture the proximal PTM-acceptor lysine on the interacting substrates with high sensitivity and accuracy, in a spatiotemporally controlled manner (Fig. 2b).

### Proteome-wide characterizing direct substrates of protein lysine acetyltransferase PatZ

We initially tested the feasibility of the designed strategy on global substrate profiling for PatZ enzyme in *E. coli*. PatZ and its homologs are the major lysine acetyltransferases in bacteria which regulate diverse biological processes, including growth, mobility, stress response, etc, but the corresponding acylation substrates are still elusive^46^. In *E. coli*, most of the protein acetylation events were reported to be non-enzymatically mediated by acetyl phosphate, thus, genetic perturbation of PatZ resulted in negligible alteration on the level of protein acetylation^47^. In addition, the previous studies from us and other groups showed PatZ and its homologs exhibit promiscuous acylation activity other than acetylation^36,48,49^. Therefore, it is challenging to assign acylation substrates of PatZ by genetic perturbation of enzymes coupled with PTM-antibody enrichment and MS analysis approach.

**Fig. 3:**
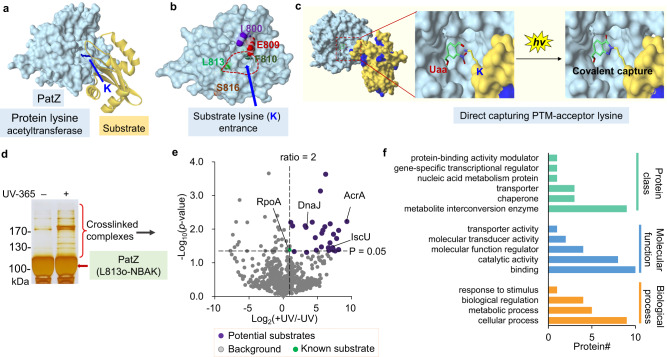
Global profiling substrates of PatZ enzyme via direct capturing strategy in living cells. **a** Schematic view of the homology-modeled catalytic domain of PatZ (shown in blue surface mode) interacting with substrate (yellow ribbon). Blue arrow indicates the substrate lysine shown in stick. **b** Structure of PatZ catalytic domain is shown in surface mode with I800, E809, F810, L813, and S816 indicated. **c** The mechanism of the light-activated primary amines and o-nitrobenzyl alcohols cyclization (PANAC photoclick reaction). **d** Sliver staining of samples from ±UV-irradiation treatment and His6-tag affinity-enrichment, with red arrow indicating PatZ-L813o-NBAK. **e** Volcano plot displaying enriched proteins (+UV/−UV ratio >2, *p* value < 0.05, purple dots) by PatZ-L813o-NBAK from biological duplicates. Statistical tests are unpaired, two-sided two-sided Student’s *t* test. Protein identification and label-free quantification were performed with MaxQuant software (see Supplementary methods for details). The abundance of a known PatZ substrate RpoA in +UV group is 1.9-fold of that in –UV group (*p* value < 0.05). **f** Annotation of enriched proteins with regards to protein class, molecular function, and biological process according to PANTHER database (v16). Source data are provided as a Source Data file. UV ultraviolet light.

To explore the appropriate sites for genetic incorporation of the o-NBAK, we homology-modeled the structure of the catalytic domain (GNAT-domain) of PatZ since no crystal structure is currently available. The crystal structure of *Sl*PatA (PDB ID: 4NXY) was selected as the template because it has a known binding mode with substrate^50^, which provided a hint of how PatZ would interact with its substrates (Fig. 3a). We initially selected three sites (I800, F810, L813) in PatZ for incorporation of the o-NBAK Uaa (Fig. 3b). I800 and F810 are located directly adjacent to a conserved residue E809 in the catalytic pocket^51^, but with distinct structural environments: F810 is deeply buried in the protein fold while I800 is exposed to solvent, and L813 is half-buried in the catalytic site (Fig. 3b). The PatZ mutants with site-specifically incorporated o-NBAK Uaa were successfully prepared according to our previous method^44^, but the PatZ-F810o-NBAK mutant showed limited expression of soluble protein (Supplementary Fig. 1a), and thus we chose those PatZ mutants incorporated with o-NBAK at sites I800 and L813.

After reaching the stationary phase, *E. coli* cells expressing PatZ-F810o-NBAK protein or PatZ-L813o-NBAK protein were subjected to 365 nm UV light activation for 20 min (+UV), and samples without UV light (−UV) activation were used as controls. After cell lysis and affinity purification, the eluent was further resolved by SDS-PAGE. UV-irradiated sample generated several higher molecular weight bands for PatZ-L813o-NBAK mutant group, which indicated the efficient crosslinking of interacting proteins (Fig. 3c, d). In contrast, the other PatZ-I800o-NBAK mutant distal to the catalytic site or unfavorably orientated, showed almost no difference between UV-irradiation samples and controls (Supplementary Fig. 1b). Moreover, we chose another distal site S816 for comparison (Fig. 3b). S816 is solvent-exposed and more far away from the active site tunnel. Thus, the incorporated o-NBAK in this distant site (S816o-NBAK) could serve as an important control for capturing non-substrate binding proteins or distal PPIs, rather than interacting substrates of PatZ (Fig. 3b). In identical conditions, PatZ-S816o-NBAK mutant showed almost non-crosslinking of interacting proteins, which was similar to the results of PatZ-I800o-NBAK mutant (Supplementary Fig. 1b). Although these two sites (I800, S816) of o-NBAKs are more solvent-exposed than L813o-NBAK, and expected to have more crosslinking possibility with surrounding proteins, the crosslinking efficiency mainly relied on suitable position of the genetically encoded photo-crosslinker o-NBAK which indicates the spatial resolution features (Fig. 3b, d, Supplementary Fig. 1b). These results demonstrate that the direct capturing efficiency largely depends on the substrate recognition and the engagement of the PTM-acceptor lysine.

Next, samples from PatZ-L813o-NBAK mutant were used for the subsequent substrate profiling. The bands with higher molecular weight than free PatZ mutant enzyme in SDS-PAGE gel with UV light (+UV) or without UV light exposure (−UV) groups were excised, respectively (Fig. 3d), which correspond to potential crosslinked enzyme-substrate assemblies. Based on the covalent crosslinking of mutant PatZ enzyme and substrates, the sequential affinity purification with washing process and SDS-PAGE separation would increase the detection sensitivity for substrates, due to the removal of high-abundance non-specific backgrounds and free PatZ mutant enzyme (Fig. 3d, PatZ-L813o-NBAK). The gel slices were then subjected to trypsin digestion, LC-MS/MS analysis and label-free quantification. A known PatZ substrate, DNA-directed RNA polymerase subunit alpha (RpoA), displayed significantly higher abundance in +UV group over –UV group. Proteins that were significantly enriched in +UV group were considered as candidate substrates (Fig. 3e and Supplementary Data 1). In total, PatZ-L813o-NBAK mutant captured 28 candidate substrates with diverse functional classes and various biological processes (Fig. 3f). More importantly, the temporally-controlled crosslinking allows us to focus on the investigation of corresponding substrates in a given growth phase (at several time points, also see Supplementary Fig. 18). Taken together, our herein developed strategy enabled direct capturing of PatZ-substrate interactions with tolerable perturbation (Supplementary Figs. 10–12) of the cellular milieu, and thus presents a straightforward and general method for the discovery of acylation substrates of lysine-modifying enzyme in a spatiotemporal manner.

### Validation of PatZ substrates and acetylation sites

**Fig. 4:**
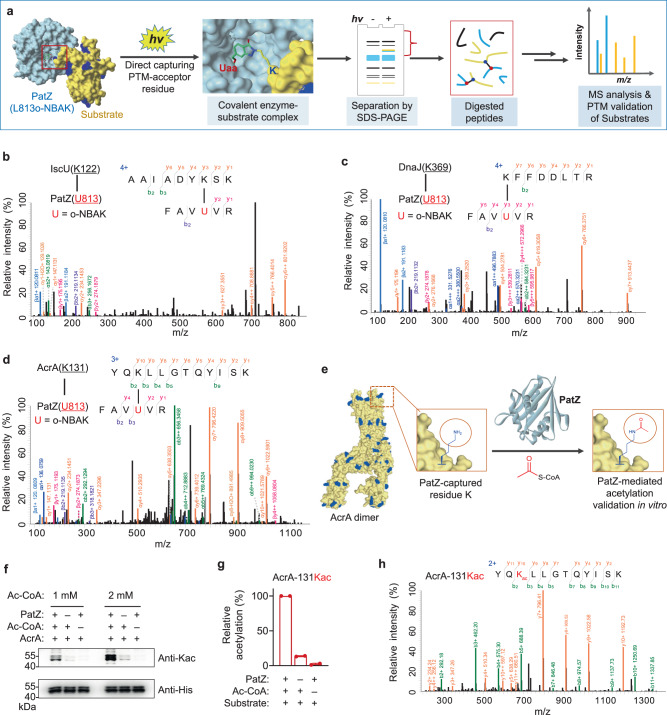
Validation of direct crosslinking of o-NBAK encoded enzyme (PatZ-L813o-NBAK) with candidate substrates, and acetylation of substrate AcrA by PatZ enzyme. **a** Schematic of the workflow of light-induced direct capturing strategy for PatZ-L813o-NBAK mutant enzyme. **b**–**d** Annotated MS/MS spectra indicating o-NBAK of PatZ mutant crosslinks with IscU-K122 (**b**), DnaJ-K369 (**c**), AcrA-K131 (**d**), respectively, U = o-NBAK. The crosslinked peptides were identified with MeroX software (see Supplementary methods for details), and the quality of the crosslink-spectrum matches was manually inspected. **e** Schematic of PatZ enzyme mediated acetylation. **f** Western blot analysis confirming lysine acetylation (Kac) of AcrA by PatZ. **g** Relative acetylation level at AcrA-131Kac under different conditions. Error bars represent standard errors of mean. *n* = 2 biologically independent experiments. **h** Annotated MS/MS spectrum of peptide bearing AcrA-131Kac, and acetylated lysine residue was red-colored. Source data are provided as a Source Data file.

To validate the direct interaction between PatZ and candidate substrates, we performed in vitro crosslinking by selecting purified recombinant candidate proteins (Fig. 4a), including a reported PatZ substrate IscU^52^, a reported PatZ interacting protein DnaJ^53^, and a distinct candidate substrate, multidrug efflux pump subunit AcrA (Supplementary Fig. 26a)^54^. The purified proteins were crosslinked with PatZ-L813o-NBAK mutant under UV irradiation for 15 min on ice and resolved on SDS-PAGE. Compared with the corresponding control groups, extra bands consistent with the expected molecular weight of crosslinking were observed in UV-irradiated mixture groups (Supplementary Fig. 2). These bands were then excised and subjected to LC-MS/MS analysis for further validation of the covalent crosslinks. We identified high-confidence crosslink-spectrum matches (CSMs) for each substrate. Extensive b- and y-ions indicated that the covalent linkages exclusively formed between o-NBAK and the lysine residues on the candidate proteins (Fig. 4b–d and Supplementary Fig. 3), which indicated the direct and residue-specific crosslinking of the L813o-NBAK with lysine residues.

Notably, encouraged by the capability to capture K122 of substrate IscU (Fig. 4b), which had previously been implied as an enzymatic acetylation site^52^, we further investigated whether the crosslinked sites in the distinct candidate substrates (Fig. 4d) could actually be acetylated by PatZ. Therefore, we carried out validation for PatZ-mediated acetylation on AcrA (Fig. 4e). Purified AcrA protein was subjected to in vitro acetylation assay, compared with samples without PatZ or acetyl-coenzyme A (Ac-CoA) incubation as controls, and then analyzed by Western blot (Fig. 4f). Although intrinsic acetylation and Ac-CoA-mediated non-enzymatic acetylation were observed in controls, the acetylation level was significantly higher in the presence of both PatZ and Ac-CoA (Fig. 4f). In addition, the mutations of several active site residues of PatZ (e.g. E809A, V814A and G824A)^51^ diminished the acetylation level to certain extents (Supplementary Fig. 4). These results confirmed the enzymatic acetylation of the substrate AcrA by PatZ enzyme. After the MS analysis of the samples from the in vitro acetylation assay, we found K131 of AcrA, which formed a covalent linkage with PatZ-L138o-NBAK in our direct substrate capturing analysis, was indeed acetylated by PatZ enzyme (Fig. 4g, h and Supplementary Fig. 5), implicating AcrA is a substrate of PatZ in vitro. Moreover, we also identified some crosslinked peptides/sites from the cell lysate samples directly for several above-mentioned candidate substrates, such as grpE, nusG, arcA, dxs, etc. (Supplementary Figs. 13, 14). Thus, the crosslinking-MS analysis is complementary to enzymatic acetylation assays to validate the protein substrates and their modified sites. Altogether, these results clearly demonstrated that our strategy was able to directly capture enzymatic substrates via crosslinking the PTM-acceptor residues.

### In vitro validation of the potential biological consequence of substrate AcrA acetylation

Protein AcrA is a previously unreported candidate substrate of PatZ enzyme, which is a component of AcrA-AcrB-AcrZ-TolC multidrug efflux pump^54^, leading to intrinsic resistance to a range of antibiotics^54^. Although acetylation is a ubiquitous PTM in bacteria with different regulatory roles^46^, the biological consequence of acetylation of AcrA has not been disclosed. The acetylation site K131 of AcrA belongs to one of the four co-evolved residue pairs in the interface between AcrA and TolC (Supplementary Fig. 26a)^54^. We reasoned that acetylation on K131 may interfere with its binding with TolC. Thus, we prepared purified recombinant TolC and wild-type AcrA (AcrA-wt), as well as genetically incorporated acetyl-lysine (Kac) into position 131 of AcrA (AcrA-K131Kac) (Supplementary Fig. 26b, c). The interactions of TolC with AcrA-wt, and TolC with AcrA-K131Kac were analyzed by bio-layer interferometry assay. After incubation of wild-type AcrA with TolC, the interaction assay led to a substantial and concentration-dependent increase of signals. In contrast, minimal signal change was observed when the mutant AcrA-K131Kac was incubated with TolC (Supplementary Fig. 26d, e), indicating the subassembly with TolC was largely abolished by the acetylation on K131. To investigate whether AcrA-131Kac affected *E. coli* drug resistance, we overexpressed wild-type AcrA and AcrA-K131Q mutant in the *acrA* gene-deleted *E. coli* strains for antibiotic susceptibility test (i.e., erythromycin, oxacillin, and linezolid), respectively. The K131Q mutation of AcrA neutralized its positive charge and therefore mimicked K131 acetylated AcrA. The growth inhibition assay results suggested that AcrA-131Kac was potentially vital to the antibiotics susceptibility of *E. coli*, as AcrA-K131Q mutant exhibited severe growth inhibition in all three tested conditions (Supplementary Fig. 26f). Taken together, these results implicated a potential role of PatZ enzyme in regulating the formation of AcrA-TolC complex via acetylation K131 of AcrA (Supplementary Fig. 26b–d), which possibly involved in the AcrA-AcrB-AcrZ-TolC multidrug efflux pump assembly^54^. These findings provide a perspective for understanding the regulatory mechanism of the multidrug efflux pump and, consequentially, the regulation of antibiotic resistance.

### Application of direct capturing strategy in substrate profiling for the recently identified acetyltransferase YiaC

**Fig. 5:**
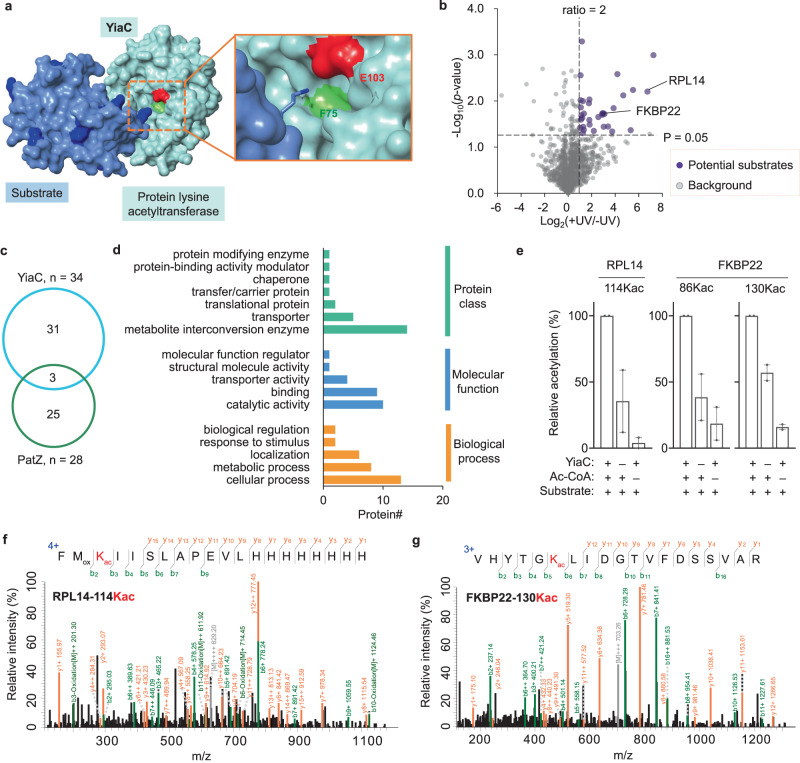
Global profiling of the substrates of YiaC enzyme. **a** Schematic view of homology-modeled YiaC (cyan) interacting with substrate (blue). The surfaces of residues F75 and E103 of YiaC enzyme are rendered in green and red, respectively. **b** Volcano plot displaying enriched proteins (+UV/−UV ratio >2, *p* value < 0.05, purple dots) by YiaC-F75o-NBAK from biological triplicates. Statistical tests are unpaired, two-sided two-sided Student’s *t* test. **c** Overlap between candidate substrates enriched by YiaC-F75o-NBAK and PatZ-L813o-NBAK. **d** Annotation of enriched proteins with regards to protein class, molecular function, and biological process according to PANTHER database (v16). **e** Relative acetylation level at K114 of RPL14, K86 and K130 of FKBP22 under different conditions. Error bars represent standard errors of mean. *n* = 2 biologically independent experiments. **f**, **g** Annotated MS/MS spectra of peptides bearing RPL14-114Kac (**f**) and FKBP22-130Kac (**g**). Acetylated lysine (Kac) residues are red-colored. *M*_ox_ Oxidized methionine. Source data are provided as a Source Data file.

With proof of concept for the identification of both known and distinct substrates of PatZ, we next tested the applicability of our strategy for YiaC, an enzyme that is conserved across bacteria taxa. YiaC has been proposed as GNAT-domain lysine acetyltransferase, but no acetylation assay was performed to validate the potential substrates^52^. A recent study has shown that YiaC has specific impacts on the global level of lysine 2-hydroxyisobutyrylation in *E. coli*^40^. In addition, YiaC in *S. enterica* has been characterized as N-terminal acetyltransferase^55^. These studies implicate YiaC might have promiscuous acyltransferase activities, thus further investigation is required to understand its substrate repertoire and function. As there is no crystal structure of YiaC, we homology-modeled its structure (Fig. 5a). Since both YiaC and PatZ adopted standard GNAT fold^52^, they are expected to have a similar binding mode with substrate lysine. Similarly, we chose F75 as the site for genetic incorporation of o-NBAK, as F75 has spatial localization similar to L813 of PatZ, and F75 is near the E103 residue, which was deduced to be engaged in catalytic reaction^52^. *E. coli* cells expressing YiaC-F75o-NBAK mutant (Supplementary Fig. 6a) with or without UV-irradiation, underwent SDS-PAGE separation and LC-MS/MS analysis. We obtained a total of 34 candidate substrates of YiaC (Fig. 5b, Supplementary Data 2). Of all these candidate substrates of YiaC, only 3 proteins were also identified as potential substrates of PatZ (Fig. 5c), which suggested minimal target redundancy between the two GNAT-domain acetyltransferases. Functional annotation analysis revealed these proteins were implicated in various biological processes (Fig. 5d).

We next validated YiaC-dependent acetylation activity for two selected candidate substrates, 50 S ribosomal protein L14 (RPL14) and FKBP-type 22 kDa peptidyl-prolyl cis-trans isomerase (FKBP22), by in vitro acetylation assay and Western blot (Supplementary Fig. 6b). The exact modification sites (K114 on RPL14, K86 and K130 on FKBP22) were confirmed by LC-MS/MS analyses (Fig. 5e–g and Supplementary Fig. 6c, d). Intriguingly, K114 of RPL14 is highly conserved and critical for binding with the ribosomal silencing factor RsfS^56^. Thus, we inferred that acetylation of K114 would likely affect this interaction, implicating a role of YiaC in regulating the translation process. Altogether, with our developed direct global capturing strategy, we revealed a collection of potential acylation substrates of YiaC and the related biological processes. Moreover, our results verified the protein lysine acetylation activity of YiaC enzyme in biochemical experiments.

### Profiling substrates of bacterial acylases LplA, TmcA, YjaB, and mammalian acyltransferases Tip60, and GCN5

**Fig. 6:**
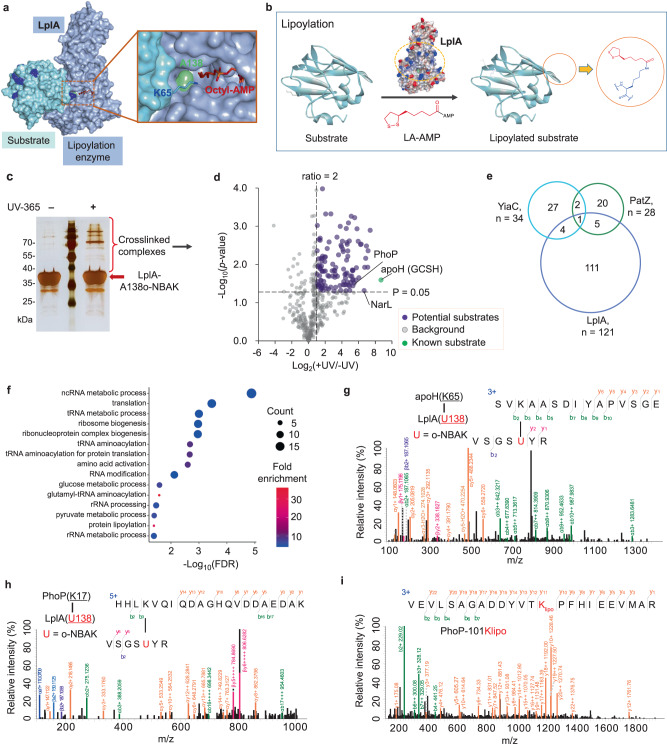
Global profiling of substrates of LplA enzyme and validation of substrate lipoylation by LplA enzyme. **a** Schematic of LplA (blue) interacting with its substrate protein (apoH protein, or called GCSH, cyan) (modified from structure, PDB ID: 3A7A). A138 (green) and the octyl-AMP (a stable surrogate for lipoic acid-AMP conjugate, LA-AMP, red stick) are shown, respectively. **b** Schematic of LplA-mediated lipoylation. The surface of LplA is colored by atom charge, with red and blue indicating positively and negatively charged surfaces. The brown dashed oval indicates binding area with substrates. **c** Sliver staining of samples from ±UV-irradiation treatment and His6-tag affinity-enrichment, red arrow indicating LplA-A138o-NBAK, and the higher molecular weight bands indicating crosslinked complexes. **d** Volcano plot displaying enriched proteins (+UV/−UV ratio >2, *p* value < 0.05, purple and green dots) by LplA-A138o-NBAK from four replicates. Statistical tests are unpaired, two-sided two-sided Student’s *t* test. **e** Venn diagram showing overlap of proteins enriched by LplA-A138o-NBAK, YiaC-F75o-NBAK, and PatZ-L813o-NBAK. **f** Gene ontology – biological process (GO-BP) enrichment analysis of enriched proteins. Significantly enriched (false discovery rate, FDR < 0.05) GO-BP terms of 15 highest fold enrichment value (color-coded) are shown, with size of the circle indicating number of proteins in the GO-BP term. **g**, **h** Annotated MS/MS spectra indicating o-NBAK of LplA mutant crosslinks with apoH-K65 (**g**) and PhoP-K17 (**h**) respectively, U = o-NBAK. **i** Annotated MS/MS spectrum of peptide bearing PhoP-101Klipo. lipo lipoylation. Source data are provided as a Source Data file.

To further evaluate our developed strategy for directly capturing substrates of another acylation type of PTM-enzymes, we selected the lysine-modifying enzyme lipoate-protein ligase A (LplA, Fig. 6a) in *E. coli*^57^. Protein lipoylation (Fig. 6b), as well as its regulatory enzymes and substrates, are all evolutionarily conserved and play essential roles in energy metabolism and amino acid catabolism; however, there are only a limited number of lipoylated substrates previously reported^57^. It remains to know the global lipoylated substrate proteins and there was no validation for lipoylation performed on the identified potential substrates. Thus, we questioned whether our direct capturing strategy could expand the inventory of potential substrates for LplA enzyme.

Based on the crystal structure of LplA (PDB ID: 3A7A), we genetically incorporated o-NBAK into site A138, which is located in the channel for substrate lysine accessing to the lipoic acid-AMP (LA-AMP) intermediate (Fig. 6a and Supplementary Fig. 7a). As a proof of the enzyme-substrate crosslinking feasibility, we performed in vitro crosslinking between o-NBAK encoded enzyme LplA-A138o-NBAK and apoH protein of the glycine cleavage system in which K65 is a reported lipoylation site by LplA^57^. Compared to controls without apoH protein or without UV-irradiation (−UV), an extra band in the UV-irradiated mixture suggested the success of crosslinking (Supplementary Fig. 7b). The following LC-MS/MS analysis of proteins in this band unambiguously confirmed the crosslink between LplA-A138o-NBAK and the expected K65 of apoH protein (Fig. 6g).

Having confirmed the ability to capture the known substrate apoH, we next turned to a global profiling of substrates of LplA enzyme. *E. coli* cells expressing His6-tagged LplA-A138o-NBAK mutant were submitted to in vivo crosslinking, subsequent lysis, and affinity purification. The UV-irradiated sample showed multiple extra bands in SDS-PAGE gel, compared to the control group, indicating efficient crosslinking (Fig. 6c). The proteins with higher molecular weight than mutant enzyme LplA-A138o-NBAK (Fig. 6c), in both groups were submitted to LC-MS/MS analysis. We identified 121 proteins significantly enriched in +UV group over –UV group, including the known substrate apoH (Fig. 6d, Supplementary Data 3A, log_2_ratio = 8.68, *p* value = 0.025), highlighting the suitability of our approach in the identification of substrates. Of note, the apoH and LplA binding is low affinity (*K*_*m*_ = 1.2 μM)^58^, indicating our strategy could capture weak substrate and PTM-enzyme interactions. It is noteworthy that <10% of the proteins enriched by LplA-A138o-NBAK were identified as candidate substrates of acetyltransferases YiaC and PatZ (Fig. 6e), suggesting the proteins were enriched based on specific enzyme-substrate recognition rather than non-specific binding. Gene ontology enrichment analysis for these enriched proteins revealed that various biological processes were significantly enriched, such as RNA metabolism, ribosome biogenesis, translation, glucose, and pyruvate metabolism, etc. (Fig. 6f, Supplementary Data 3B).

We then performed in vitro crosslinking validation for a candidate substrate of LplA enzyme (Supplementary Fig. 27a), the transcriptional regulatory protein PhoP, which plays central roles in the control of transcription of virulence genes in several gram-negative bacterial species and is considered an intriguing drug target^59^. Compared to controls, additional bands with higher molecular weight showed up in the UV-irradiated LplA-A138o-NBAK and candidate mixture (Supplementary Fig. 7c). The following tandem MS analysis confirmed crosslinking between LplA and PhoP (Fig. 6h). Then, in vitro enzymatic assay was conducted to validate the lipoylation by wild type LplA. We identified 3 lysine lipoylation residues of PhoP in the presence of LplA enzyme and LA-ATP (Fig. 6i, and Supplementary Fig. 27b, c) based on the high-confidence peptide-MS spectrum matches and unambiguous site localization. In contrast, no lipoylation was identified in the control groups that lacked LplA or LA-ATP. In addition, we further confirmed another potential substrate, the nitrate/nitrite response regulator protein (NarL) of LplA enzyme. We identified 2 lysine lipoylation residues of NarL (Supplementary Fig. 27d, e), which suggested that NarL was the substrate of the LplA enzyme and was readily lipoylated by LplA. In addition, from the crosslinking-MS analysis, we could identify crosslinked peptides/sites from the cell lysate samples directly for some above-mentioned candidate substrates, such as galR, gapA, hisS, narH, rlmL, rpmB, etc. (Supplementary Figs. 13, 14), which suggested that our method was capable to capture PTM-acceptor lysine of substrates for other acylation, other than acetylation. Taken together, these results indicated that our strategy was able to capture the potential substrates of another type of acylation enzymes, such as lipoylation enzyme LplA.

Finally, to demonstrate more acyltransferases incorporating o-NBAK at the catalytic site tunnel to capture substrates, we expanded our strategy to other bacterial lysine acyltransferases TmcA, and YjaB, as well as mammalian acyltransferases Tip60 and GCN5. The TmcA enzyme, previously known as a RNA acetyltransferase, has recently been reported as a lysine 2-hydroxyisobutyryltransferase in the regulation of transcription and acid resistance of *E. coli*., but few substrates have been characterized^40^. Our o-NBAK-incorporated TmcA captured dozens of candidate substrates (Supplementary Figs. 22, 23, Supplementary Data 4) in *E. coli* cells, and some of them were validated by in vitro acylation assay using wild-type enzyme (e.g., 2-hydroxyisobutyrylation of MTOX and FolC proteins, Supplementary Fig. 23). Indeed, we also could identify some crosslinked peptides/sites from the cell lysate samples directly, including lysines of substrates such as acnB-K571, aspS-K123, gatD-K341, hsdR-K669, leuS-K34, pepD-K59, etc. (Supplementary Figs. 13, 14). Another acetyltransferase YjaB was reported to regulate the acetylation levels for a number of proteins, but no direct substrate has been reported to date^52^. Again, our direct substrate capturing method could capture a variety of candidate substrates (Supplementary Fig. 22b, 24, Supplementary Data 5) in *E. coli* cells, and some of the candidate substrates were confirmed by in vitro enzymatic assay (e.g., EF-Ts, Rho and Rob proteins, Supplementary Fig. 24). We further demonstrated this method in mammalian acyltransferase substrate identification. The mammalian acyltransferase Tip60 enzyme plays important roles in many processes, such as chromosome segregation and DNA damage repair^60,61^. In living human 293 F cells, using our direct substrate capturing strategy, we identified 89 candidate substrates (Supplementary Fig. 19, 20, Supplementary Data 6) for Tip60, including several previously reported substrates, such as H4 protein, H2A protein, and Ran protein. Another example is the mammalian acyltransferase GCN5, which is crucial for chromatin function regulation^61^. Our strategy has uncovered substrate profiles of GCN5, providing dozens of candidate substrates (Supplementary Fig. 19, 21, Supplementary Data 7), as well as previously reported substrates, such as H4 protein. Altogether, these results indicated our direct substrate-capturing strategy could successfully be applied in different acyltransferases in both bacteria and mammalian cells.

## Discussion

By integrating enzymatic PTM mechanisms, residue-selective crosslinking chemistry, and tolerable perturbation to the cellular enzymes, we have established a direct capturing strategy for global substrate profiling of lysine-modifying enzymes in living cells, conferring irreversibly covalent enzyme-substrate complexes for purification and enrichment tolerating stringent processing, subsequent quantitative proteomic analysis. Based on our developed strategy, we have extended the inventory of acylation substrate candidates of PTM-enzymes, such as protein lysine acylases PatZ (Fig. 3a), YiaC (Fig. 5a), LplA, (Fig. 6a), TmcA (Supplementary Fig. 22a) and YjaB (Supplementary Fig. 22b) in *E. coli*., as well as mammalian acyltransferases Tip60 (Supplementary Fig. 19a) and GCN5 (Supplementary Fig. 19b). In addition, we have confirmed the enzymatic modifications (i.e., acetylation by PatZ and YjaB, promiscuous acylation by YiaC, and lipoylation by LplA, 2-hydroxyisobutyrylation by TmcA) for a selection of the putative substrates. Our direct substrate capturing approach is capable of identifying the directly interacting substrates in a spatiotemporal-resolved manner (Fig. 3b, Supplementary Figs. 1b, 17, 18), as well as capturing and validating of acylation site of the substrates in a straightforward fashion.

PTMs of lysine residues are crucial for cellular processes from gene expression to signal transduction. In current studies, our strategy was applied to decode substrate profiles of five different protein lysine acylases PatZ, YiaC, TmcA, YjaB, and LplA, as well as two mammalian acyltransferases Tip60 and GCN5, which not only retrieved known substrates but also identified candidate substrates. Several candidates were validated by in vitro crosslinking and enzymatic assay, and the two assays agreed well with each other regarding the modification sites, which further corroborated the concept of our strategy. In addition, this approach also could identify crosslinked peptides/sites from the cell lysate samples directly (Supplementary Figs. 13, 14). Remarkably, biochemical studies on acetylation sites (AcrA-131Kac) regulated by PatZ showed a potential role of PatZ in intrinsic antibiotic drug resistance of bacteria (Fig. 4e–h and Supplementary Fig. 26), suggesting that the AcrA-131Kac was potentially vital to the antibiotic susceptibility of *E. coli* (Supplementary Fig. 26). Moreover, we revealed various potential functional substrates of YiaC, such as the YiaC-dependent acetylation on K114 of RPL14 in translation process. Furthermore, in our work, many putative substrates of LplA have been revealed, which are involved in diverse biological processes (Fig. 6f). After the analysis of the interfaces of enzyme-substrate (Supplementary Fig. 8a), we found that the lipoylation sites of potential substrates PhoP and NarL might be recognized by LplA enzyme in a mode similar to the known substrate apoH (Supplementary Fig. 8b–e). Compared with the acetylation of K102 and K201 of *St*PhoP in *S. Typhimurium*^39,62^, we revealed the lipoylation of K101 (Supplementary Fig. 9) and K200 on PhoP in *E. coli*. possibly have an effect on the function of PhoP to suppress the transcriptional activity and block DNA binding, suggesting a potential role of LplA in the regulation of bacterial virulence. Of note, in these substrate profiling studies, the photo-crosslinker o-NBAK was incorporated into the catalytic site tunnel of the acyltransferases (Supplementary Figs. 10, 19, 22), rather than on the interaction interface of PPIs, thus the o-NBAK mainly captures the proximal PTM-acceptor lysines (within appropriate distance and suitable angle, Supplementary Fig. 17) on interacting substrates, conferring the spatial resolution in our substrate capturing process.

Indeed, despite several excellent crosslinking strategies for capturing protein and interacting partner (or ligand) interactions^17–26,28–30^, as well as GECX strategy^27,32–34^, methods for direct capturing substrates of protein-modifying enzymes remain largely unexplored with an aim to capture bona fide substrates but not the non-substrate binding proteins^31^. On the other hand, the potential steric hindrance would be caused by the side chain of Uaas, thus, the o-NBAK should be incorporated at the appropriate position in the active site tunnels of acylases (Supplementary method, guideline for the selection of appropriate sites). Based on the insights into the structures of acylases that the active site tunnels of acylases are relatively large^38^ (Supplementary Fig. 10), and our investigation of the perturbation to the enzyme-substrate interactions with o-NBAK incorporation at appropriate position in active site tunnels of the target enzymes (Supplementary Fig. 11), as well as our success in capturing previously known substrates for several enzymes (e.g., Fig. 3e, RpoA; Fig. 6d, apoH; Supplementary Fig. 20, histone H4, histone H2A, Ran; Supplementary Fig. 21, histone H4), we demonstrated that the o-NBAK incorporated at appropriate sites on PTM-enzymes could have tolerable perturbation to the enzyme-substrate interactions. As demonstrated in our work, the direct capturing strategy for global substrates of PTM-enzymes crucially depends on tolerable perturbation to the cellular enzyme-substrate interacting complex formation, the chemical mechanism of a certain PTM, and the accessibility of PTM-acceptor residues in proximity to the enzyme active site for residue-specific covalent crosslinking. Our developed strategy that spatiotemporally directly captures global substrates of PTM-enzymes in living cells has several features: (i) Genetic incorporation of a light-induced residue-selective crosslinker into enzyme catalytic site tunnel, which not only introduces tolerable perturbation to native enzymes (Supplementary Fig. 11), but also allows direct and spatiotemporal capture of PTM-acceptor residues on interacting substrates in situ (Fig. 3b, Supplementary Fig.1b, 17, 18), deciphering substrates with multiple lysine modifications as well as the precise modification sites of the substrates. (ii) In comparison with genetically encoded non-selective photo-crosslinkers, our lysine-selectivity photo-crosslinkers o-NBAK yielded higher overall enrichment efficiency (Supplementary Figs. 15, 16, AbK Uaa, etc.), thus, conferring higher sensitivity for substrate capturing. (iii) As substrate profiling solely based on the identification of crosslinked peptides via MS/MS analysis would result in high-level of false negative result (i.e., failure to identify a large number of bona fide substrates), owing to the intrinsic technical challenge of XL-MS experiment^63,64^, we used global substrate profiling criteria based on the identification of captured proteins via XL-MS with stringent data filtration criteria (i.e., proteins required to be identified across all replicates in either UV-treat or control group, and with ratio >2 and *p* value < 0.05 were considered as candidate substrates), which would help in decreasing false negative result and excluding elusive false positive substrates. (iv) Lysine acyltransferases are known to have promiscuous activity, i.e., one enzyme can catalyze different types of lysine acylation. Although our method could not identify a certain type of lysine modifications directly, we can determine the crosslinked peptides/sites by MS/MS analysis (Figs. 4b–d, Figs. 6g, h, Supplementary Figs. 3, 13, 14). In principle, our approach is capable of identifying all types of acylation substrates of a given acyltransferase in current studies rather than a single type of PTM substrate. Subsequent enzymatic acylation assays, or modification-specific antibody-based enrichment experiments, combined with crosslinked peptides/sites (Fig. 4b–d, Fig. 6g, h, Supplementary Figs. 3, 13, 14) will be helpful to further characterize a certain type of lysine acylation substrates and PTM-sites (Figs. 4f–h and 5e–g, Supplementary Figs. 12, 23b, c, 24b, c, 25). Thus, our method provides a complementary approach to the genetic perturbation-based method for acyltransferase research. Collectively, integrating these features, the developed strategy for direct capturing substrates of PTM-enzymes confers higher sensitivity and accuracy for the characterization of enzyme-substrate interactions, and, thus, the substrate profiles of PTM-enzymes.

The lysine-selectivity-based direct capturing strategy in living cells is expected to have broad applicability^41^. Over the past two decades, incorporation of Uaas into protein of interest has provided a widespread and powerful method for capturing protein-protein interactions in living cells^19,20,22^, while the capturing strategy may not work in the case of structures unknown. Harnessing the power in protein structure prediction, for the PTM-enzymes with structure unknown, the structures could be predicted based on the recent advance of the accurate protein structure prediction with AlphaFold^65^. Therefore, integrating the advancement of genetic code expansion technology and protein structure prediction, it is worth to note that our direct capturing strategy could be applied to capture substrates of other acylation enzymes, such as enzymes for malonylation, glutarylation, crotonylation, lipidation, and so on. In principle, the lysine-selectivity-based capturing chemistry also could be expanded to other lysine involved interactome, such as determining ligand-receptor interactions, mapping protein interfaces, and so on^20–22^. Thus, this strategy expands the landscape of enzyme-centric methods to investigate substrate profiles of lysine-specific PTM-enzymes (e.g., lysine acylome) and protein interactive proteomics^36,42^.

In conclusion, the direct capturing substrates of PTM-enzymes strategy offers a chemical tool for global substrate profiling of PTM-enzymes in living cells, largely expanding the substrate profiles of PTM-enzymes, such as the bacterial PatZ, YiaC, LplA, TmcA and YjaB, and the mammalian GCN5 and Tip60. With high residue-selectivity, spatiotemporal resolution, covalent capture, and tolerable perturbations introduced to the native PTM enzymes, this strategy enables decoding substrates in higher sensitivity and accuracy, as well as validation of the acylation site of the substrates in a straightforward manner. The concept of direct capturing substrates of PTM-enzyme could inspire and lead to further development of advanced capturing strategies based on other kinds of residue-selectivity crosslinking chemistry harnessing very recent advances^22^ for other types of PTM-enzymes, to expand the repertoire of enzyme-centric methods investigating more PTMs and the landscape of substrate-enzymes interactive proteomics.

## Methods

### Site-specific incorporation of o-NBAK into proteins, expression, and purification

The plasmid pBK-*Mm*PylRS-384F/306 A encoding the evolved PylRS and its cognitive tRNA, which inserts o-NBAK into the in-frame amber codon site on target genes in *E. coli* cells was described in the previous literature^44^. For more information on choosing the sites on target enzymes that could be incorporated with Uaas, please see Supplementary Information. Plasmids pTAK-PatZ-I800TAG, pTAK-PatZ-F810TAG, pTAK-PatZ-L813TAG, pTAK-PatZ-S816TAG, pTAK-YiaC-F75TAG, pTAK-LplA-A138TAG, pTAK-TmcA-R457TAG, or pTAK-YjaB-F77TAG were co-transformed with pBK-*Mm*PylRS-Y384F/Y306A into BL21(DE3) complement cells (50 µL) using heat shock. The transformants were recovered in 500 µL SOC media at 37 °C for 1 hour before plating to 2YT agar plate containing 50 µg/mL of kanamycin and 34 µg/mL of chloramphenicol. A single colony from the plate was picked and used to inoculate 5 mL 2YT containing 50 µg/mL kanamycin and 34 µg/mL chloramphenicol. A 2-mL aliquot of overnight culture was then used to inoculate 200 mL 2YT containing the same concentrations of antibiotics. The cells were grown at 37 °C until OD600 reached ~0.5. The culture was divided into two 100 mL portions. One portion of the culture was supplemented with 1 mM o-NBAK, and the other portion served as a control by adding medium at the same volume. After culturing for another 20 min at 37 °C, the protein expression was induced by adding 0.5 mM IPTG at 30 °C for 8 hours. The cells were pelletized in 50-mL conical tubes by centrifugation at 4200 × *g* for 30 min at 4 °C and stored at −80 °C.

Next day, the cell pellets were re-suspended in 10 mL lysis buffer. After incubation on ice for 30 min, the lysates were further treated with ultrasonication. Following centrifuge, the supernatants were incubated with 100 µL Ni-NTA agarose beads at 4 °C for 2 hours with gentle shaking. The resin was centrifuged briefly and washed with 10 column volume (CV) of lysis buffer (devoid of lysozyme) and 10 CV of washing buffer. Finally, the protein was eluted with elution buffer. A 10-µL aliquot from the elution fraction was mixed with one-fourth amount of 5× SDS loading buffer and heated at 98 °C for 8 minutes, before loading onto SDS-PAGE gel.

### Expression and photo-crosslinking of o-NBAK incorporated enzyme in 293-F cells

FreeStyle™ 293-F cells were cultured in SMM 293-TII Expression Medium and passaged every 2–3 days, starting with the cell density around 0.2 × 10^6^ cells per ml. Transfection was performed at cell density ~2.0 × 10^6^ cells per ml. Polyethyleneimine molecular mass 40,000 (PEI, 1 mg/mL) of 60 μL was diluted in 1 mL SMM 293-TII Expression Medium. DNA mixture (Tip60-C369TAG or GCN5-E575TAG: PylRS = 1: 3; or empty vector: PylRS = 1:3) of 20 μg was diluted in 1 mL SMM 293-TII Expression Medium. Diluted DNA and PEI solution were mixed and incubated at room temperature for 15 min before adding to the cell culture. o-NBAK (0.5 mM) was added immediately after transfection for o-NBAK incorporation.

Forty to forty-eight hours after transfection, the cells were collected, washed with cold PBS twice and illuminated for 15 min at 10 mm distance from a ZF-7A 16 W 365 nm UV light on ice. The cells were collected, washed with cold PBS, lysed ultrasonically in cooled RIPA lysis buffer supplemented with protease inhibitor cocktail. The lysates were centrifuged at 15,000 × *g* for 30 min at 4 °C. The supernatant was obtained and normalized by BCA protein assay. The lysates were subjected to incubation with MagStrep type3 XT bead at 4 °C overnight. The beads were washed with twofold diluted RIPA lysis buffer and wash buffer (100 mM Tris•HCl, 150 mM NaCl, 1 mM EDTA, pH = 8) three times each. Proteins attached to the beads were eluted in 2× Laemmli buffer by heating at 95 °C for 8 min. The eluates were resolved by SDS-PAGE and visualized by silver staining. Gel lanes above the free Tip60 or GCN5 enzyme were cut for subsequent in-gel digestion.

### In vitro photo-crosslinking reactions

Purified o-NBAK-containing proteins (in PBS buffer 150 mM NaCl, pH 7.5) were mixed with partner proteins (in PBS buffer 150 mM NaCl, pH 7.5), the mixture was divided and transferred to a 96-well plate (BD Biosciences), and irradiated at 10 mm distance from a handheld 365-nm UV lamp for 15 minutes on ice. Samples were collected, diluted with SDS loading buffer, and analyzed by SDS-PAGE.

### Photo-crosslinking of o-NBAK incorporated proteins in living *E. coli* cells

For each sample, BL21(DE3) cells were co-transfected plasmid pBK-*Mm*PylRS-384F/306 A, as well as pTAK-PatZ-I800TAG, pTAK-PatZ-F810TAG, pTAK-PatZ-L813TAG, pTAK-PatZ-S816TAG, pTAK-YiaC-F75TAG, pTAK-LplA-A138TAG, pTAK-TmcA-R457TAG, or pTAK-YjaB-F77TAG. The cells were grown in 2YT medium containing 50 µg/mL of kanamycin and 34 µg/mL of chloramphenicol overnight, and then were used to inoculate 200 mL 2YT containing the same concentrations of antibiotics. The cells were grown at 37 °C until OD600 reached ~0.5, and 1 mM o-NBAK was supplemented. After culturing for another 20 min at 37 °C, the protein expression was induced by adding 0.5 mM IPTG at 30 °C for 5 hours. Cells were harvested, washed twice with PBS (Gibco), and re-suspended to PBS. The mixture was divided into two portions. One portion was transferred to sixwell plate (3–4 mL/well) and irradiated at 10 mm distance with a handheld UV lamp (365-nm) for 20 min on ice. The other portion was kept in dark acting as the control group. The cells were harvested by centrifugation at 1000 × *g* for 5 min and lysed in lysis buffer. The lysate was incubated with Ni-NTA agarose beads at 4 °C for 2 hours with gentle shaking. The beads were then washed with wash buffer three times. The proteins were finally eluted with elution buffer, concentrated using a 0.5 mL centrifugal filter, and separated by SDS-PAGE. Then the gel was visualized by coomassie blue stain or silver stain.

### In vitro enzymatic acylation assay

The in vitro enzymatic acylation was performed in the reaction buffer containing 20 mM sodium phosphate (pH 8.0), 150 mM NaCl, 10% glycerol, 0.5 mM tris(2-chloroethyl) phosphate (TCEP). The in vitro acetylation by PatZ was carried out by incubation of PatZ (20 μg), acetyl-CoA (with concentrated indicated in Fig. 4 for AcrA; or 0.1 mM for DnaJ, HflX), and candidate substrates (30 μg) in 100 μL reaction buffer at 37 °C for 2 hours. The in vitro acetylation by YiaC was performed in 50 μL reaction buffer containing YiaC (4 μg), acyl-CoA (1 mM for acetyl CoA; 0.5 mM for 2-hydroxyisobutyryl-CoA, propionyl-CoA, or malonyl-CoA), and candidate substrates (20 μg) at 37 °C for three hours. The in vitro acetylation by TmcA was performed in 50 μL reaction buffer containing TmcA (15 μg), 2-hydroxyisobutyryl-CoA (0.5 mM), ATP (1 mM), and candidate substrates (20 μg) at 37 °C for three hours. The in vitro acetylation by YjaB was performed in 50 μL reaction buffer containing YjaB (4 μg), Ac-CoA (0.5 mM), and candidate substrates (20 μg) at 37 °C for three hours. The samples were resolved by SDS-PAGE for subsequent western blot analysis, or in-gel digestion and LC-MS/MS analysis.

### Western blot analysis

Loading buffer with additional *β*-Me (2%) to denature the protein sample, protein samples in SDS-PAGE sample buffer were boiled for 8 min, resolved on an SDS-PAGE gel, and transferred to PVDF membrane (Thermo Fisher Scientific). The membrane was blocked in 5% skim milk in TBST (50 mM Tris, 150 mM NaCl, 0.05% Tween-20, pH 7.5) at room temperature for 1 hour. Then the membrane was incubated with mouse anti-His tag antibody (1:10000, Proteintech, Cat. No. HRP-66005), mouse anti-vinculin monoclonal antibody (1:2000, Cat. No. 66305-1-Ig), mouse monoclonal anti-acetyl lysine antibody (1:1000, Cell Signaling Technology, Cat. No. 9681), mouse anti-strep-tag II monoclonal antibody (1:2000, Abbkine, Cat. No. ABT2230), rabbit polyclonal FtsZ antibody (1:1000, CUSABIO, Cat. No. CSB-PA359270HA01EGX) in TBST at 4 °C overnight. The membrane was washed with TBST (4 × 5 min) before the addition of the secondary goat anti-mouse horseradish peroxidase conjugate (1:4000, Proteintech, Cat. No. SA00001-1) or HRP-conjugated goat anti-rabbit antibody (1:5000, Cell Signaling Technology, Cat. No. 7074). After 1 hour, the membrane was washed with TBST (4 × 5 min). Final detection of HRP activity was performed using ECL Plus chemiluminescent substrate (Thermo Fisher Scientific). The image was acquired with Image Quant LAS 4000 or ChemiScope 6300. Uncropped images can be found in the Source Data File.

### Bio-layer interferometry (BLI) assay

The binding characteristics between dN-AcrA-wt or dN-AcrA-K131Kac and TolC were determined using anti-HIS (HIS2) biosensors in the Octet RED96 system (ForteBio Inc., Menlo Park, CA, USA). The membrane protein TolC was loaded onto the HIS2 biosensors at 4 μM in PBS containing 150 mM NaCl, 0.03% DDM, and 5% glycerol. The biosensors were blocked with His8-tagged MBP protein (2 mg/ml) for 60 s. Diluted AcrA in PBS solution containing 150 mM NaCl, 0.03% DDM, and 5% glycerol was then added to the HIS2 biosensors loaded with TolC. The real-time binding response (Δλ in nanometer, nm) between AcrA and TolC was calculated by subtracting the non-specific binding of AcrA to the HIS2 biosensors from the binding of AcrA with TolC.

### In vitro lipoylation reaction

The in vitro lipoylation reaction was performed in the buffer containing 40 mM PBS (pH 7.0), 0.5 mM dithiothreitol (DTT), 10 μM LplA, 20 μM PhoP or NarL, 2 mM ATP, 2 mM MgCl_2_, 1 mM lipoic acid for 3 h at 37 °C. Then, the reaction mixture was separated by SDS-PAGE and visualized by Coomassie blue stain.

### In-gel digestion

The gel slices containing bands of interest were destained with 50% ethanol. After being balanced with distilled water, the bands were cut into cubes (~1 mm^3^) and dehydrated in acetonitrile (ACN). The cubes were then incubated with 25 mM NH_4_HCO_3_ containing 10 mM DTT at 56 °C for 1 h and then with 25 mM NH_4_HCO_3_ containing 55 mM iodoacetamide (IAA) at r.t. for 45 min in darkness. The gel cubes were then dehydrated in 50% ACN, vacuum-dried, and incubated with 50 mM NH_4_HCO_3_ containing 10–20 ng trypsin (sequencing grade, Hualishi Scientific, Beijing) at 37 °C overnight. For in vitro PatZ-substrate crosslinking, LplA-apoH crosslinking, crosslinking, and lipoylation reactions of LplA-NarL, samples were further incubated with 10–20 ng endoproteinase Glu-C (sequencing grade, Roche) at 37 °C for additional 4 h. The peptides were sequentially extracted with 50% ACN (containing 5% TFA), 75% ACN (containing 0.1% TFA) and 100% ACN, and vacuum-dried. The peptides were desalted with ZipTip C18 pipette tips (Merck-Millipore, Darmstadt, Germany) and ready for subsequent LC-MS/MS analysis.

### LC-MS/MS analysis

The peptides were reconstituted in buffer A (0.1% formic acid, 2% ACN) and loaded onto 15 cm C18 reversed-phase capillary analytical column (3 μm particle size, 90 Å pore size, Dikma Technologies, Lake Forest, CA) connected to an EASY-nLC 1000 HPLC system (Thermo Fisher Scientific, Waltham, MA).

For analyses of the samples from substrate profiling by PatZ-L813o-NBAK crosslinking in living cells, the peptides were eluted with a linear gradient of buffer B (0.1% formic acid, 90% ACN) from 8% to 32% in 58 min, followed by an increase to 48% in 6 min and further increase to 80% in 2 min at a constant flow rate of 300 nL/min. The samples from crosslinking substrate profiling in living cells (by YiaC-F75o-NBAK, LplA-A138o-NBAK, TmcA-R457o-NBAK, YjaB-F77o-NBAK, GCN5-E575o-NBAK or Tip60-C369o-NBAK), substrate validation by in vitro enzymatic assays (catalyzed by wild type YiaC, LplA, TmcA, and YjaB), the peptides were eluted with a linear gradient of buffer B from 8% to 13% in 20 min, followed by an increase to 26% in 31 min, to 45% in 5 min and to 80% in 1 min at a constant flow rate of 300 nL/min. The eluted peptides were ionized and sprayed into an Orbitrap Fusion mass spectrometer (Thermo Fisher Scientific, Waltham, MA) in a positive mode. The mass spectrometric analysis was carried out in a “Top speed” data-dependent acquisition (DDA) mode with a cycle time of 3 s. The peptides with a range of m/z 350–1300 were analyzed by an Orbitrap mass analyzer with a resolution of 120,000, automatic gain control (AGC) target of 5 × 10^5^, and maximum ion injection time (IT) of 50 ms. The dynamic exclusion was set as 60 s, and the charge inclusion was set to 2~6+. The isolated precursor ions were subjected to fragmentation via higher-energy collisional dissociation (HCD) with a normalized collision energy (NCE) of 32%, and analyzed by an ion trap analyzer.

For analyses of samples from in vitro acetylation by wt YiaC or lipoylation by wt LplA, the setting for LC and MS are the same as above except that the dynamic exclusion was set as 30 s, and the charge inclusion was set to 2~4+ for MS1 analysis.

For analyses of samples from in vitro acetylation by wt PatZ, the peptides were eluted with a linear gradient of buffer B from 7% to 35% in 24 min, followed by an increase to 80% in 3 min. The eluted peptides were ionized and sprayed into an Orbitrap Fusion mass spectrometer in a positive mode. The mass spectrometric analysis was carried out in a “Top 20” DDA mode. The peptides with a range of m/z 350–1500 were analyzed by an Orbitrap mass analyzer with a resolution of 240,000, AGC target of 7 × 10^5^, and IT of 50 ms. The dynamic exclusion was set as 10 s, and the charge inclusion was set to 2~4+. The isolated precursor ions were subjected to fragmentation via collision-induced dissociation with an NCE of 35%, and analyzed by ion trap analyzer.

To identify crosslinking sites, the peptides were eluted with a linear gradient of buffer B from 5% to 7% in 13 min, followed by an increase to 10% in 20 min, 25% in 55 min, 45% in 22 min, 80% in 3 min at a constant flow rate of 300 nL/min. The eluted peptides were ionized and sprayed into an Orbitrap Fusion mass spectrometer in a positive mode. The mass spectrometric analysis was carried out in a “Top speed” DDA mode with a cycle time of 3 s. The peptides with a range of m/z 300–1700 were analyzed by an Orbitrap mass analyzer with a resolution of 120,000, AGC target of 5 × 10^5^, and maximum IT of 100 ms. The dynamic exclusion was set as 60 s, and the charge inclusion was set to 3~7+. The isolated precursor ions were subjected to fragmentation via HCD with an NCE of 27–33% (stepped collision energy = 3%), and analyzed by Orbitrap analyzer at a resolution of 15,000, automatic AGC target of 2 × 10^5^ and IT of 250 ms, the dynamic exclusion was set as 10 s.

All LC-MS/MS data were collected with XCalibur (3.0). For identification of proteins in experiments of substrate profiling by enzyme-o-NBAK mutants, raw MS data were processed with MaxQuant software (v. 1.6.6.0)^66^ against UniProt *Escherichia coli* database (2019.2 release, 4350 sequences) or human database (2019.2 release, 95943 sequences). For data from the in vitro acetylation assay, MS raw data were analyzed by Mascot search engine (v2.3, Matrix Science, London, UK)^67^ against the corresponding protein sequences. For the identification of crosslinks, raw data were searched against MeroX search engine (v2.0)^68^ against the sequences for proteins of interest. For details, see Supplementary Information. The MS/MS spectra of identified acylated or crosslinked peptides were annotated by Mascot or MeroX software, respectively. In some cases, the annotated MS/MS spectra were displayed using pLabel^69^ for better visualization.

### Reporting summary

Further information on research design is available in the Nature Portfolio Reporting Summary linked to this article.

### Supplementary information

Supplementary Information

Reporting Summary

Dataset 1

Dataset 2

Dataset 3

Dataset 4

Dataset 5

Dataset 6

Dataset 7

Description of Additional Supplementary Files

Peer Review File

### Source data

Source data file

## Data Availability

All the mass spectrometry proteomics raw data and the database search result files have been deposited to ProteomeXchange consortium via the iProX partner repository under the dataset identifier PXD040318. The GO-BP enrichment analysis was performed with PANTHER database (v16). Protein structures were retrieved from PBD: *Sl*PatA (PDB ID: 4NXY), YjaB (PDB ID: 2KCW), LplA (PDB ID: 3A7A), PhoP (PDB ID: 2PKX; 2PL1), NarL (PDB ID: 1RNL), TmcA (PDB ID: 2ZPA), GCN5 (PDB ID: 1Z4R), Tip60 (PDB ID: 2OU2). The processed proteomics data and the GO-BP enrichment analysis data are available in the Supplementary Data files. Other data generated in this study are provided in the Supplementary Information/Source Data file. Source data are provided in this paper.
