# Supplementary material for: Spatiotemporal and direct capturing global substrates of lysine-modifying enzymes in living cells
Source: Nat Commun. 2024 Feb 17;15:1465. doi: 10.1038/s41467-024-45765-3 (PMC10874396; doi:10.1038/s41467-024-45765-3)

Supplementary Information

Spatiotemporal and direct capturing global substrates of post-translational modification enzymes in living cells

Hao Hu,[1,7] *Wei Hu,*[1,7] *An-Di Guo,*[1,7] *Linhui Zhai,*[1,2,7] *Song Ma,*[1] *Hui-Jun Nie,*[1] *Bin-Shan Zhou,*[1] *Tianxian Liu,*[1] *Xinglong Jia,*[1] *Xing Liu,*[3] *Xuebiao Yao*[3], *Minjia Tan*[1,4,5 *] *Xiao-Hua Chen*[1,4,6 *]

[1] State Key Laboratory of Drug Research, Shanghai Institute of Materia Medica, Chinese Academy of Sciences, Shanghai, 201203, China

[2] Translational Research Institute of Brain and Brain-Like Intelligence, Shanghai Fourth People's Hospital, School of Medicine, Tongji University, Shanghai, 200434, China

[3] MOE Key Laboratory for Cellular Dynamics and Hefei National Center for Physical Sciences at the Microscale, University of Science and Technology of China, Hefei, 230026, China.

[4] University of Chinese Academy of Sciences, Beijing 100049, China

[5] Zhongshan Institute for Drug Discovery, Shanghai Institute of Materia Medica, Chinese Academy of Sciences, Zhongshan, Guangdong, 528400, China

[6] School of Pharmaceutical Science and Technology, Hangzhou Institute for Advanced Study, University of Chinese Academy of Sciences, Hangzhou, 310024, China.

[7] These authors contributed equally to this work.

***Correspondence:** xhchen@simm.ac.cn, mjtan@simm.ac.cn

Table of contents

Supplemental Figures 3

Supplementary Note 1. Guideline for the selection of appropriate photoUaa incorporation sites. 37

Supplemental Methods 38

General Information 38

1. Protein expression and purification 38

2. *In vitro* photo-crosslinking reactions 45

3. Photo-crosslinking of o-NBAK (or other photoUaas) incorporated acyltransferases in living cells 46

4. *In vitro* enzymatic activity assays 46

5. Western blot analysis 47

6. Biolayer interferometry (BLI) assay 47

7. Profiling of protein-protein interactome (PPI) by BS2G-mediated crosslinking 47

8. In-gel digestion 48

9. LC-MS/MS analysis 48

10. MS data analysis 49

11. Bioinformatics. 50

12. Homology modeling and protein-protein docking. 51

13. Drug sensitivity assay. 51

References 52

Uncropped scans of blots and gels in Supplementary Figures 54

Supplemental Figures

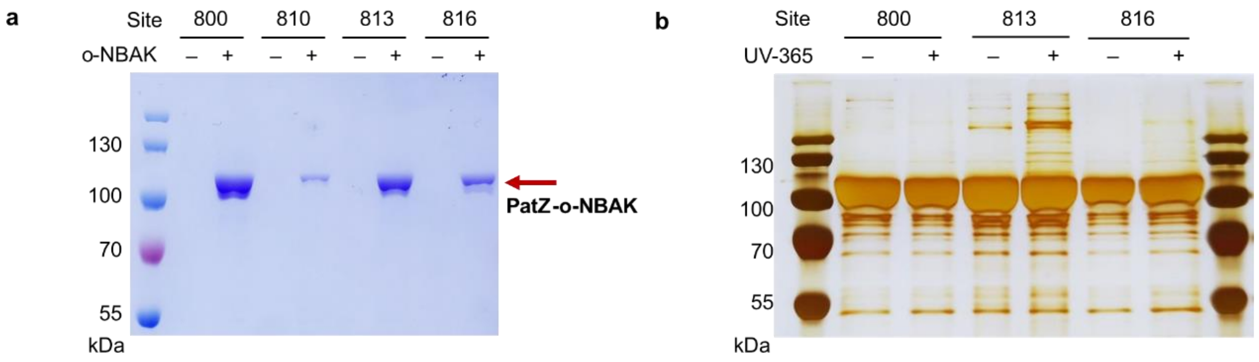

Supplementary Fig. 1. Genetic incorporation of o-NBAK into PatZ and *in situ* crosslinking. a, SDS-PAGE analysis of incorporation of o-NBAK into PatZ mutants. **b**, Sliver staining of samples from *in situ* ±UV-irradiation treatment, cell lysis, His6-tag affinity-enrichment and SDS-PAGE analysis.

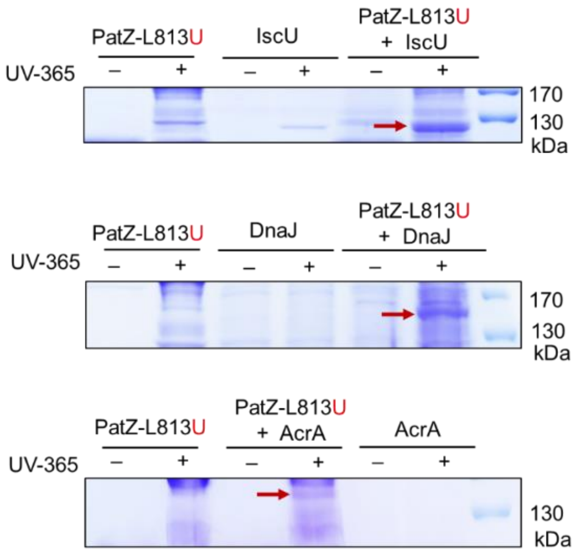

Supplementary Fig. 2. SDS-PAGE analysis of *in vitro* crosslinking between PatZ-L813o-NBAK and candidate substrates IscU, DnaJ and AcrA. PatZ-L813o-NBAK, candidate substrates and their mixture treated with UV-365 irradiation or not were subjected to SDS-PAGE analysis. Extra bands (marked by red arrows) in the lane of UV-365-treated mixture compared to other lanes indicated crosslinking complexes. These extra bands were sliced and subjected to in-gel digestion and LC-MS/MS analysis for identification of crosslinked peptides, the spectra of which are shown in Fig. 4b-d in the main text and Supplementary Fig. 3. U = o-NBAK. Source data are provided as a Source Data file.

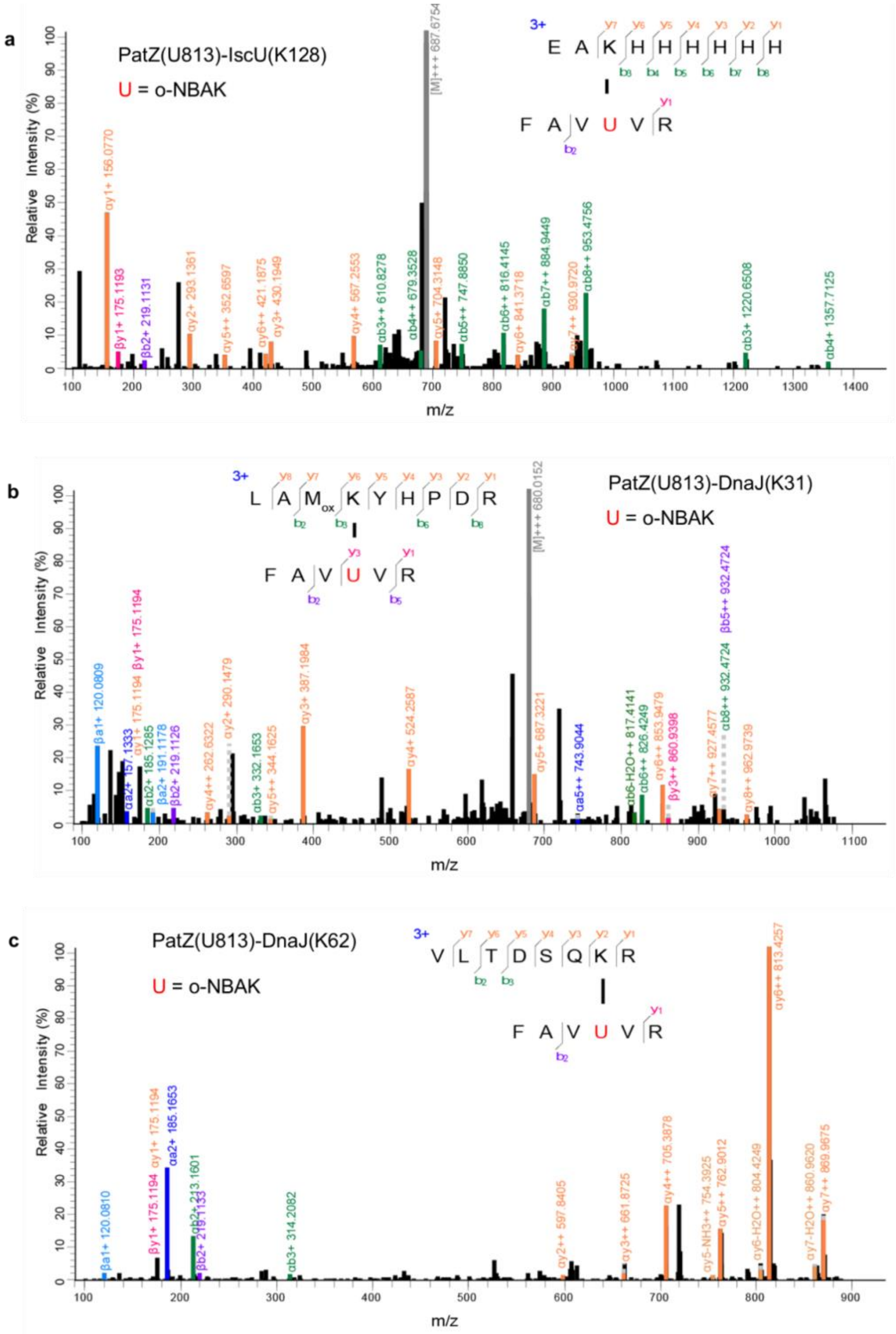
a
PatZ(U813)-IscU(K128)
U = o-NBAK
E A K H H H H H H
F A V U V R
Relative Intensity (%)
m/z
b
PatZ(U813)-DnaJ(K31)
U = o-NBAK
L A M K Y H P D R
F A V U V R
Relative Intensity (%)
m/z
c
PatZ(U813)-DnaJ(K62)
U = o-NBAK
V L T D S Q K R
F A V U V R
Relative Intensity (%)
m/z

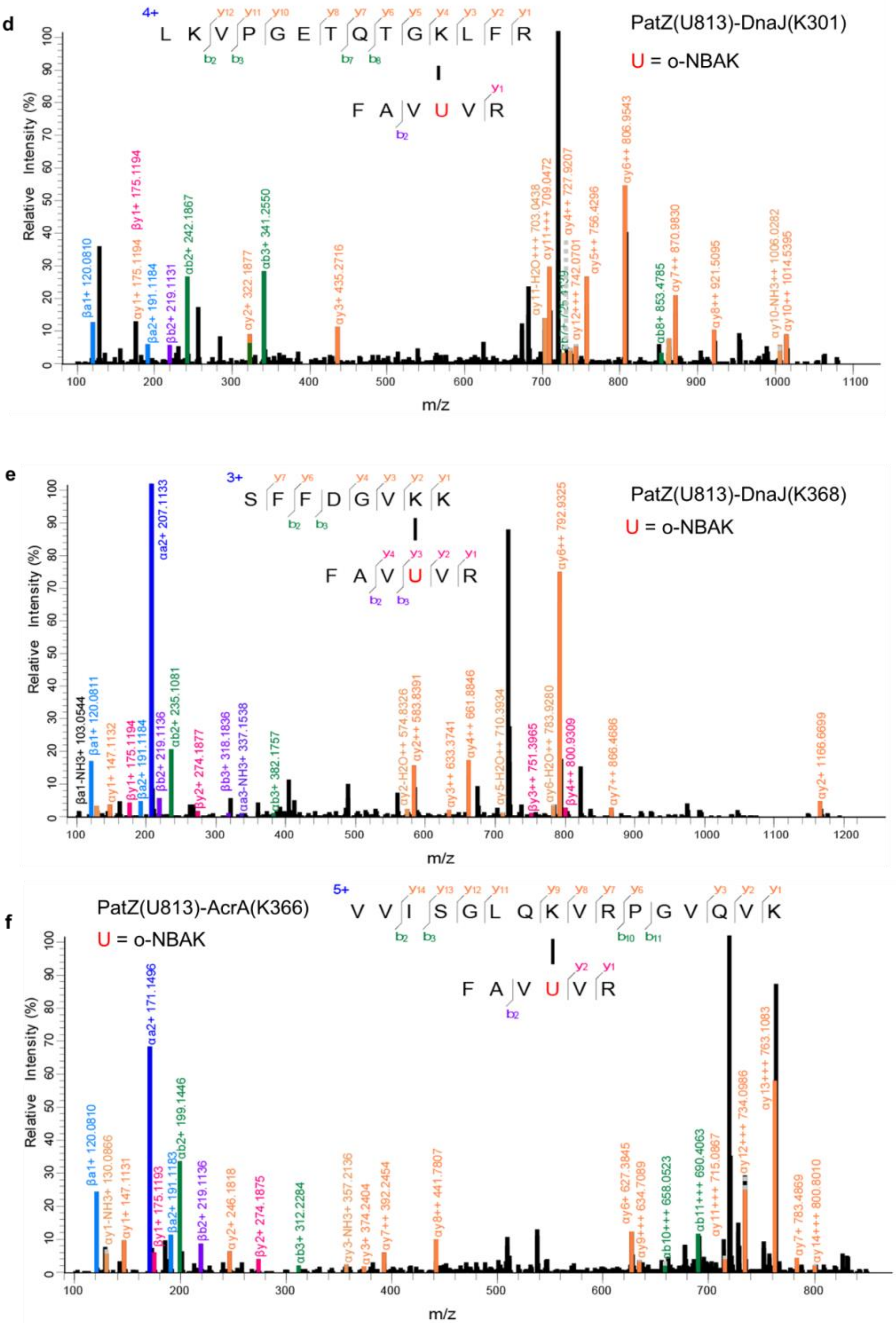

Supplementary Fig. 3. Annotated MS/MS spectra of crosslinked peptides between PatZ-L813o-NBAK and candidate substrates. Extensive b-ions and y-ions unambiguously confirmed the precise crosslink sites which are indicated in parentheses.

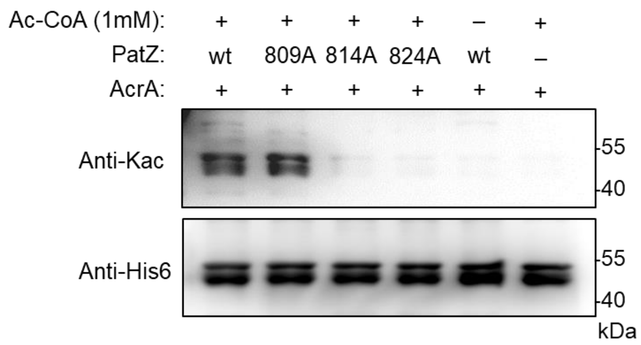

Supplementary Fig. 4. Western blot analysis of enzymatic acetylation of ArcA by wild type PatZ and mutants. Samples in absence of Ac-CoA or wild-type (wt) PatZ were used as controls to indicate intrinsic acetylation or non-enzymatic acetylation, respectively. The mutated sites were selected according to the proposed active sites in previous study.[1] E809A mutation on PatZ displayed no apparent effect on the acetylation of AcrA. This is consistent with previous study[1] in which E809 was reported to only slightly facilitate acetylation at physiological pH. V814 and G824 on PatZ are critical for Ac-CoA binding and conserved in multiple GNAT-domain acetyltransferases, thus it is within expectation that the V814A or G824A mutations complete abolished the enzymatic acetylation on AcrA. These results corroborated the enzymatic mechanism of the acetylation on AcrA by PatZ. Source data are provided as a Source Data file.

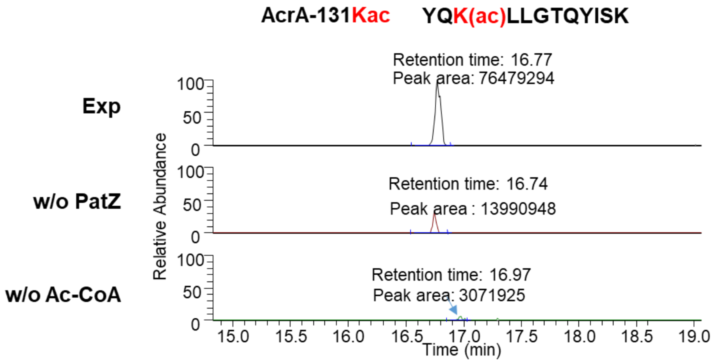

	PatZ	Ac-CoA	Substrate
Exp	+	+	+
w/o PatZ	-	+	+
w/o Ac-CoA	+	-	+

Supplementary Fig. 5. Extracted ion currents of acetylated peptides from different assay conditions. In **Exp** group, substrate was incubated with PatZ enzyme and Ac-CoA. In **w/o PatZ** or **w/o Ac-CoA** groups, substrate was only incubated with Ac-CoA or PatZ enzyme, respectively. The peak areas of acetylated peptides in **Exp** group are apparently larger compared with other two conditions, indicative of PatZ-dependent acetylation.

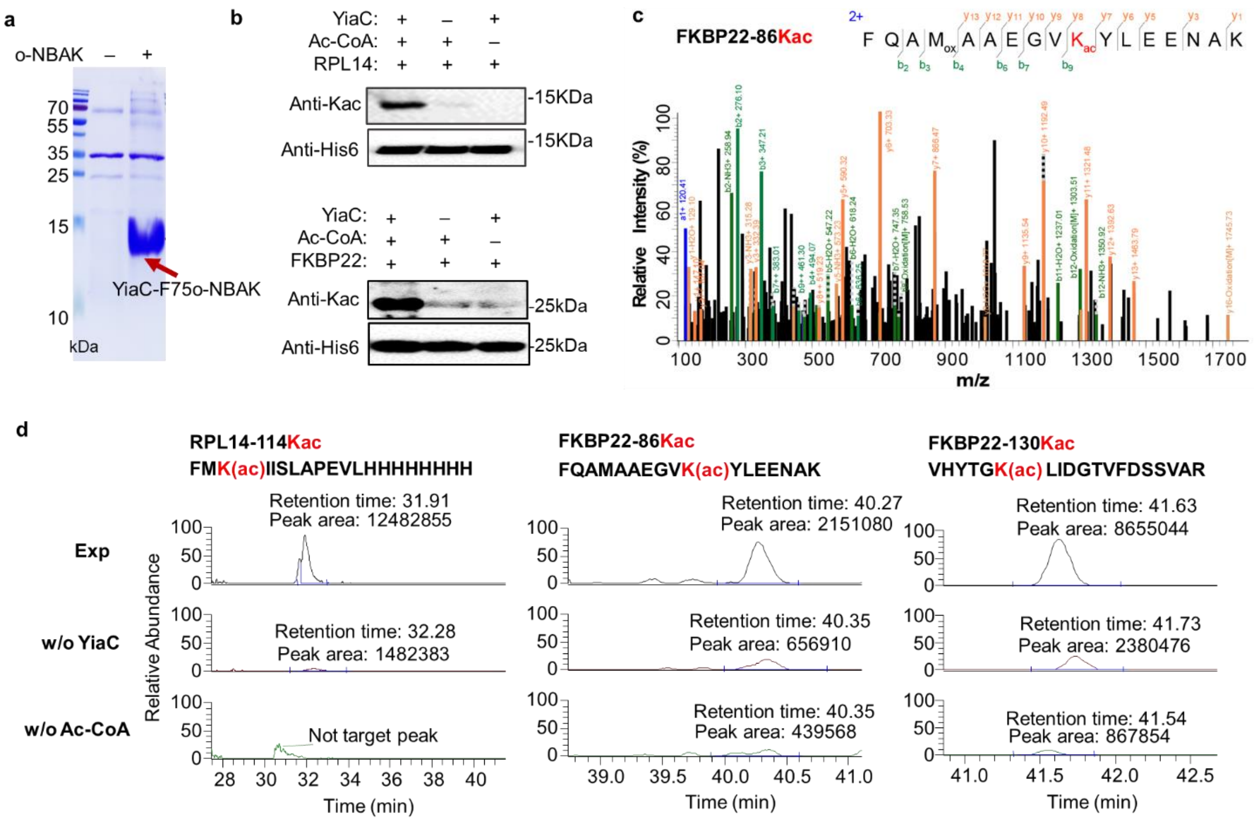

Supplementary Fig. 6. Genetic incorporation of o-NBAK into of YiaC and *in vitro* acetylation by YiaC. a, SDS-PAGE analysis of incorporation of o-NBAK into position 75 of YiaC. **b**, Western blot analyses confirming acetylation of RPL14 or FKBP22 by YiaC *in vitro*. The acetylation level is monitored by anti-Kac antibody and the loading amount of substrate is monitored with anti-His-tag antibody. The signals in samples containing YiaC, Ac-CoA, substrate (RPL14 or FKBP22) are much stronger than other two conditions, suggesting YiaC-dependent acetylation. **c**, Annotated MS/MS spectrum of peptide bearing FKBP22-86Kac. **d**, Extracted ion currents of acetylated peptides from different assay conditions. In **Exp** group, substrate was incubated with YiaC enzyme and Ac-CoA. In **w/o YiaC** or **w/o Ac-CoA** groups, substrate was only incubated with Ac-CoA or YiaC enzyme, respectively. For both substrates RPL14 and FKBP22, the peak areas of acetylated peptides in **Exp** group are apparently larger than other two conditions, indicative of YiaC-dependent acetylation. Source data are provided as a Source Data file.

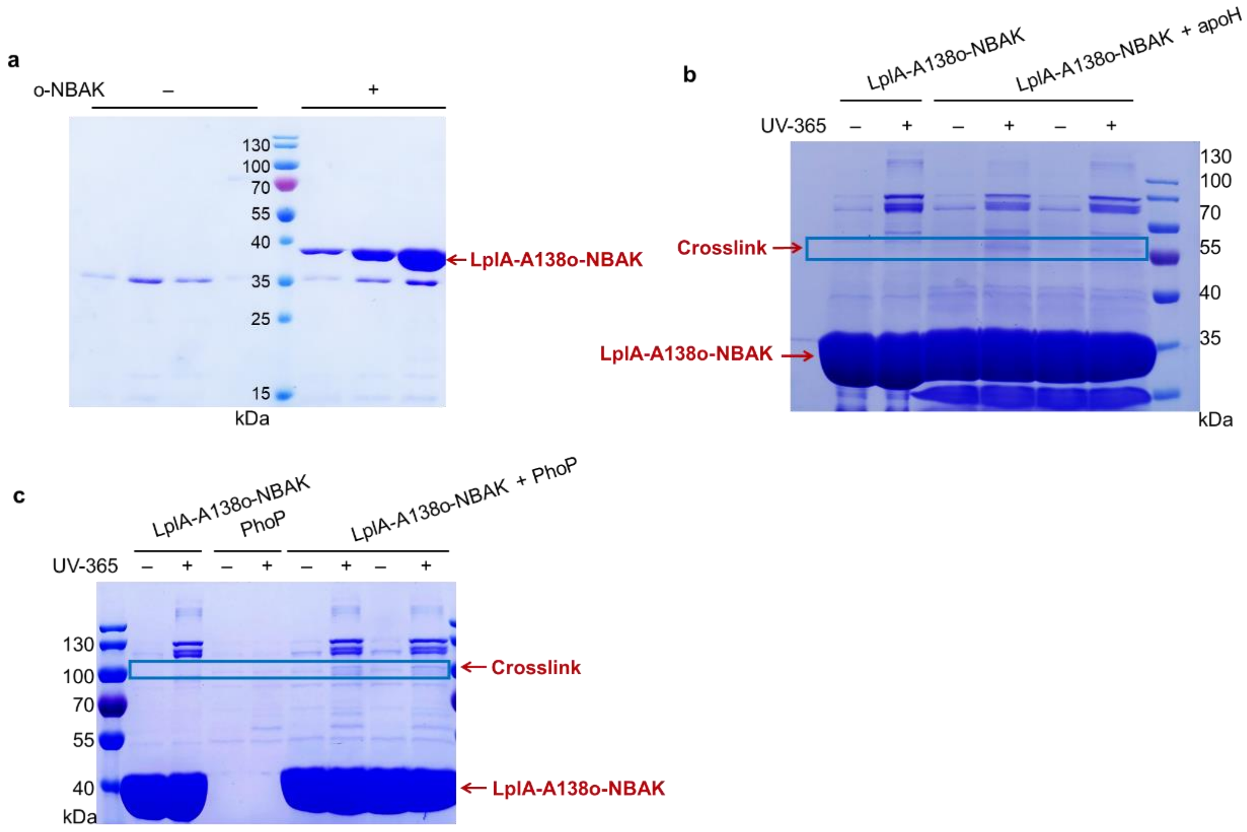

Supplementary Fig. 7. Characterization for substrates of LplA-A138o-NBAK. a, SDS-PAGE analysis of incorporation of o-NBAK into position 138 of LplA with serial concentrations of protein in different lanes. Only in the presence of o-NBAK, the LplA-A138o-NBAK protein was expressed. **b**, *in vitro* crosslinking between purified LplA-A138o-NBAK and known substrate apoH protein. Bands in blue box indicate crosslinked proteins. **c**, *in vitro* crosslinking between purified LplA-A138o-NBAK and potential substrate PhoP protein. Bands in blue box indicate crosslinked proteins.

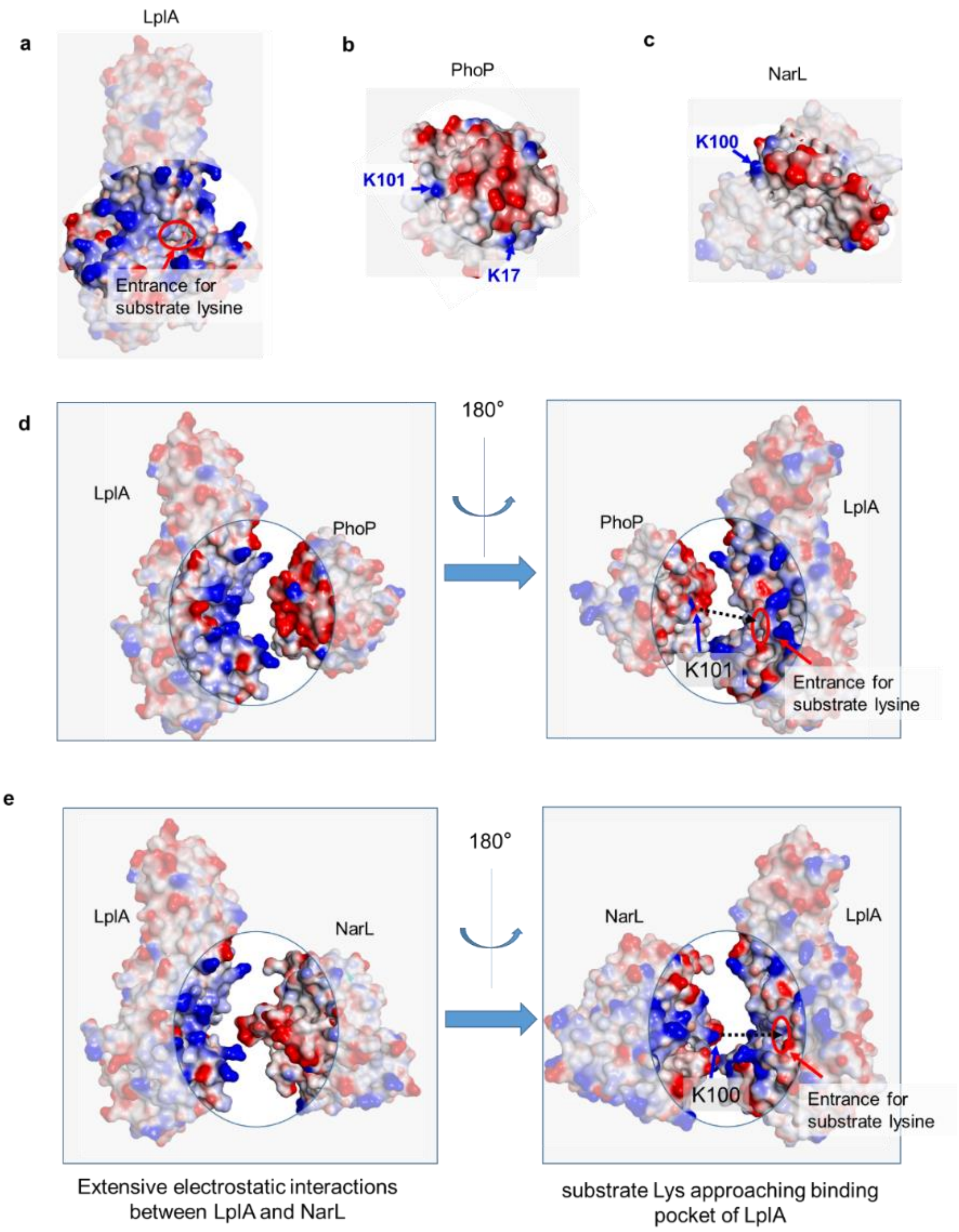

Supplementary Fig. 8. Electrostatic interactions between LplA enzyme and candidate substrates PhoP and NarL. a-c, Surface of the structures of LplA (PDB ID: 3A7A), PhoP (PDB ID: 2PKX) and NarL (right, PDB ID: 1RNL), which are rendered by atom charges. Positively and negatively charged surfaces are shown in blue and red. Blue arrows indicating lysine residues lipoylated by LplA *in vitro*. The surface of LplA enzyme is covered with "positively charged patch" (**a**), which is supposed to mediate the recognition of substrate by interacting with acidic residue clusters on the substrate surface.[2] Indeed, we found several lipoylated lysines (e.g. K17 and K101 of PhoP, and K100 of NarL) located close to clusters of acidic residues (**b-c**), suggesting they might be recognized by LplA enzyme in a mode similar to the known substrates apoH.[2] The schematic binding model between LplA enzyme and candidate substrates PhoP and NarL are shown in (**d**) and (**e**), respectively. The binding model of LplA-PhoP is generated by GRAMM-X Protein-Protein Docking Web Server[3] (v.1.2.0), with the distance of the two proteins slightly adjusted for better visualization. The relative position between LplA and NarL are manually adjusted for visualization of the electrostatic interactions.

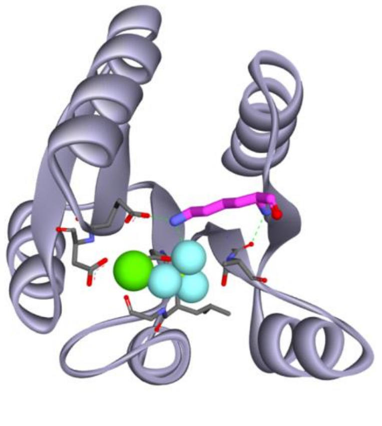

PhoP bound with a phosphoryl analog (BeF_3)

Supplementary Fig. 9. Structure of PhoP (PDB ID: 2PL1) bound with Mg^{2+} ion (green ball) and the activating agent berrylium fluoride (a phosphoryl analog, cyan ball). The PhoP-K101 residue is shown in bold stick with carbon atom in purple. The green dashes indicate hydrogen bonds between K101 and adjacent Glu residue and fluoride. K101 of PhoP in *E. coli* is conserved and participates in PhoP activation by forming essential salt bridges with nearby Glu and activating agent BeF_3 in the active site. [4] It has been reported that acetylation of K102 of *St*PhoP (corresponding to K101 in *E. coli*) suppressed transcriptional activity.[5] In addition, in *S. Typhimurium*, the acetylation of K201 of *St*PhoP (corresponding to K200 in *E. coli*) had also been reported to regulate the Salmonella virulence by disrupting the interaction with DNA.[6] In these regards, it is conceivable that lipoylation of these sites would have similar effects since lipoyl group also neutralizes the positive charge of lysine and has even larger size than acetyl group.

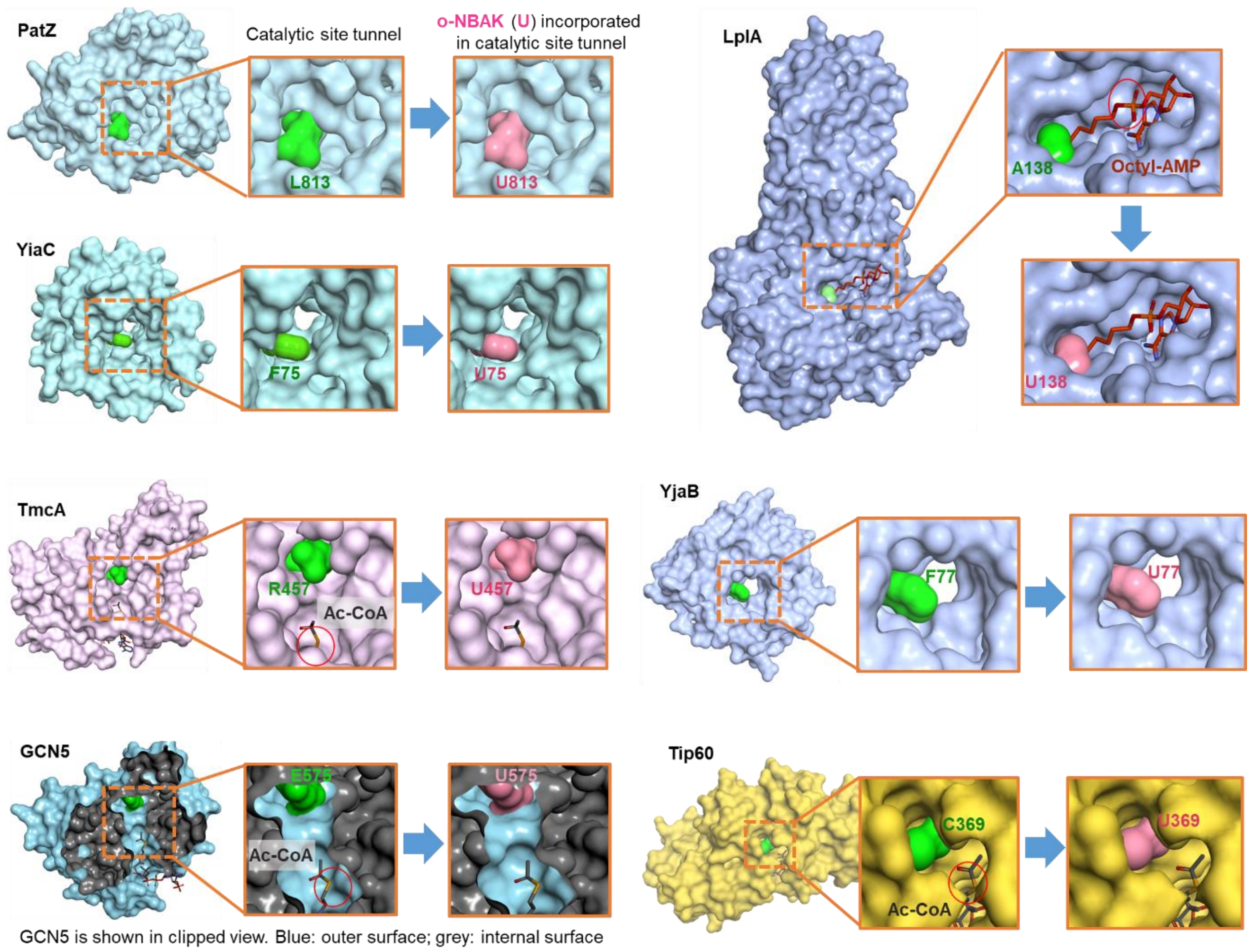

Supplementary Fig. 10. Structures of model enzymes in this study and the residue sites for the incorporation of o-NBAK. PatZ (catalytic domain is shown) and YiaC enzymes are homology-modeled (please see Supplementary Experimental Procedures for details). For other enzymes, the structures were downloaded from PDB: LplA (PDB ID: 3A7A), TmcA (PDB ID: 2ZPA, catalytic domain is shown), YjaB (PDB ID: 2KCW), GCN5 (PDB ID: 1Z4R, catalytic domain is shown) and Tip60 (PDB ID: 2OU2). The enlarged areas show the substrate lysine binding pockets and the residues (in green surface mode) to be mutated to o-NBAK. Note that, the pink-colored residues only indicate the incorporation sites of the o-NBAK (U), rather than their structures. In the structure of LplA, the octyl-AMP molecule (a stable surrogate for the cofactor LA-AMP) is shown in red stick. In the structures of TmcA, GCN5 and Tip60, the bound Ac-CoA molecules are shown in stick. The bonds in cofactors to be reacted with substrate lysine molecules are shown by red circles, indicating where the substrate lysine molecules bind. In GCN5, since the E575 is covered by other residues in the front view, the clipped view is shown for better visualization of the spatial location of E575 and lysine binding pocket (Note that E575 still has access to the lysine binding pocket).

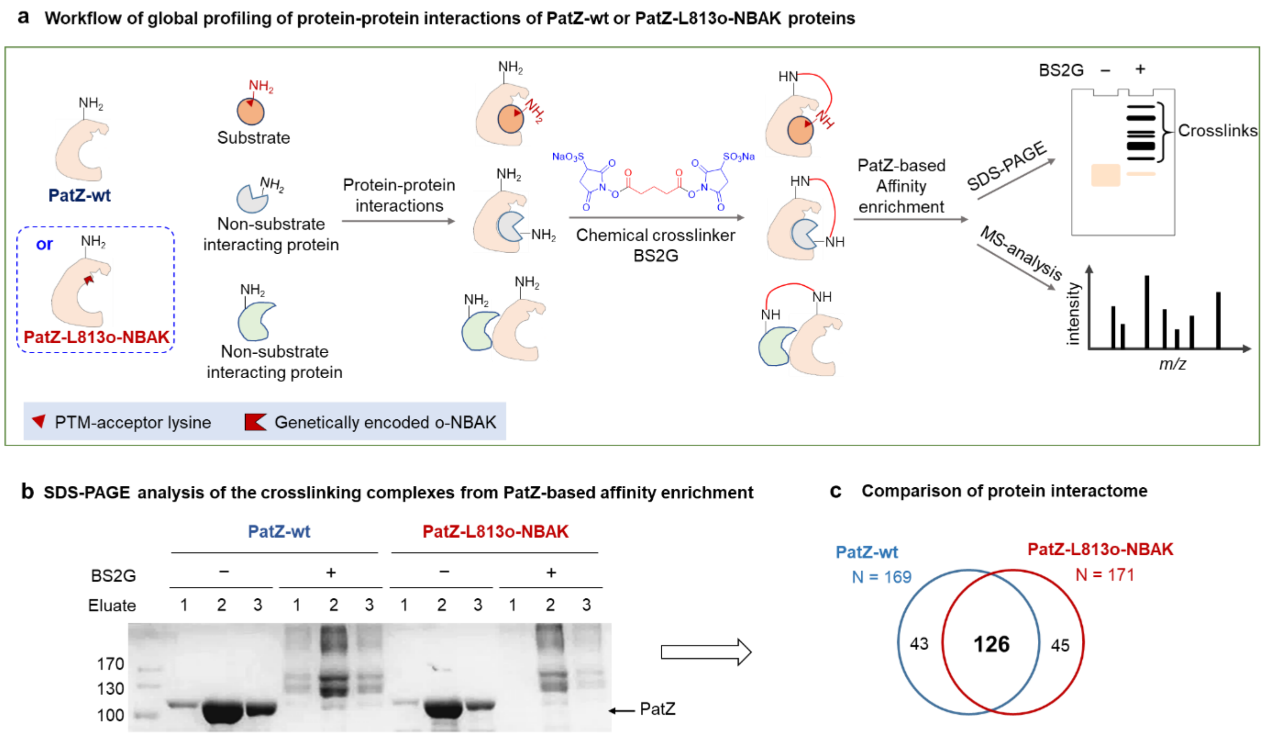

Supplementary Fig. 11. Global profiling of protein-protein interactions of PatZ-wt or PatZ-L813o-NBAK proteins. a, workflow of profiling interacting proteins of PatZ-wt (or PatZ-L813o-NBAK) in *E. coli* cell lysate. **b**, SDS-PAGE analysis of eluate from affinity purification. **c**, Venn diagram showing protein-protein interactome overlap between PatZ-wt and PatZ-L813o-NBAK. Proteins significantly enriched (ratio > 2 and p-value < 0.05 from triplicate experiments) in BS2G-treated group were considered as protein interactome of PatZ. Source data are provided as a Source Data file.

a

PatZ-catalyzed Kac sites	Normalized ratios of MS intensities		
	Experimental group	Control group 1 (C 1)	Control group 2 (C 2)
	PatZ +	PatZ −	PatZ −
	Ac-CoA +	Ac-CoA +	Ac-CoA −
	Substrate +	Substrate +	Substrate +
DnaJ-368Kac	2.2	1	1
DnaJ-369Kac	6	1.5	1
HflX-147Kac	4	1	1

b

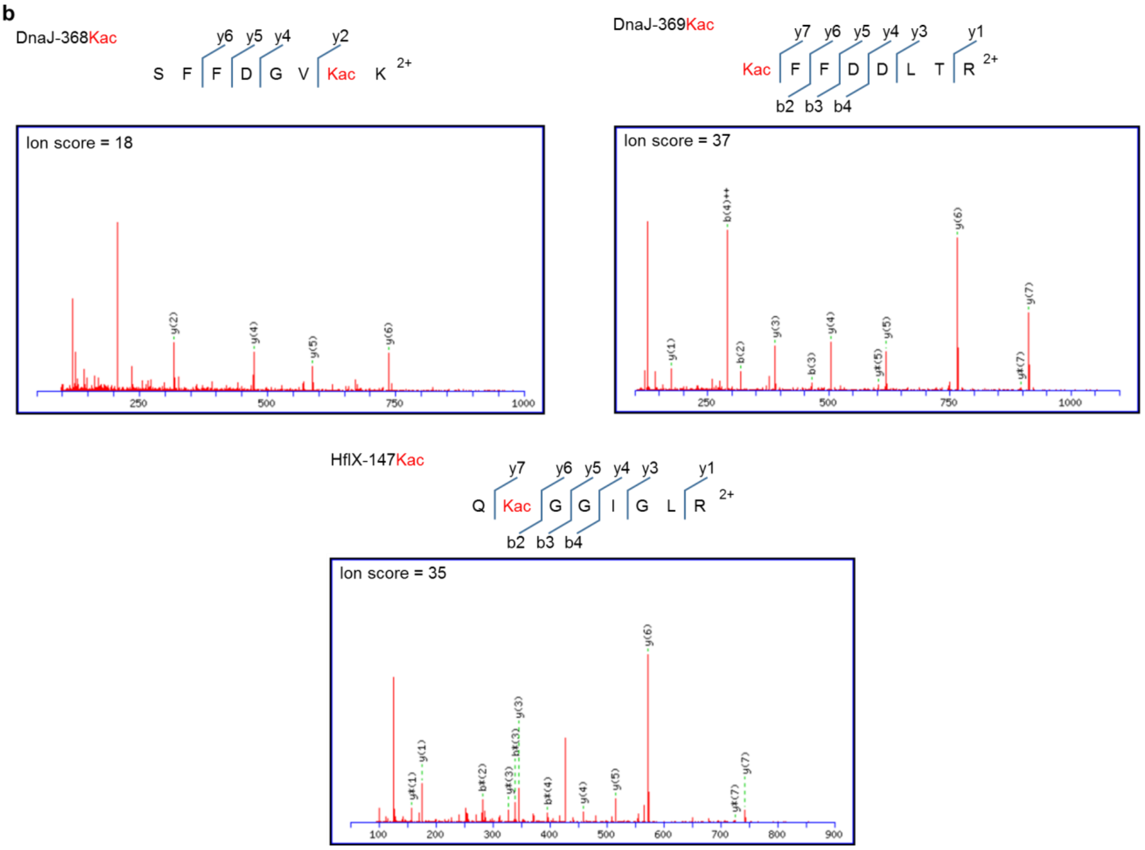

Supplementary Fig. 12. *In vitro* acetylation of candidate substrates DnaJ and HflX by PatZ-wt. a, Summary of wild type PatZ-catalyzed lysine acetylation (Kac) sites of candidate substrates and the normalized ratios of the Kac levels in three tested groups. The tested groups were set as follows: in experiment group, purified candidate substrate DnaJ or HflX protein was incubated with PatZ-wt and Ac-CoA; sample without PatZ was used as control for non-enzymatic acetylation (control 1, C1); sample only containing substrate was used as control for intrinsic acetylation level (control 2, C2). The normalized ratios were calculated as follows: the ratios of peak areas of acetylated peptides from different testing groups were calculated, and then normalized against the averaged ratio of the peak areas of 3-4 unmodified peptides. **b,** Annotated MS/MS spectra are shown for acetylated peptides.

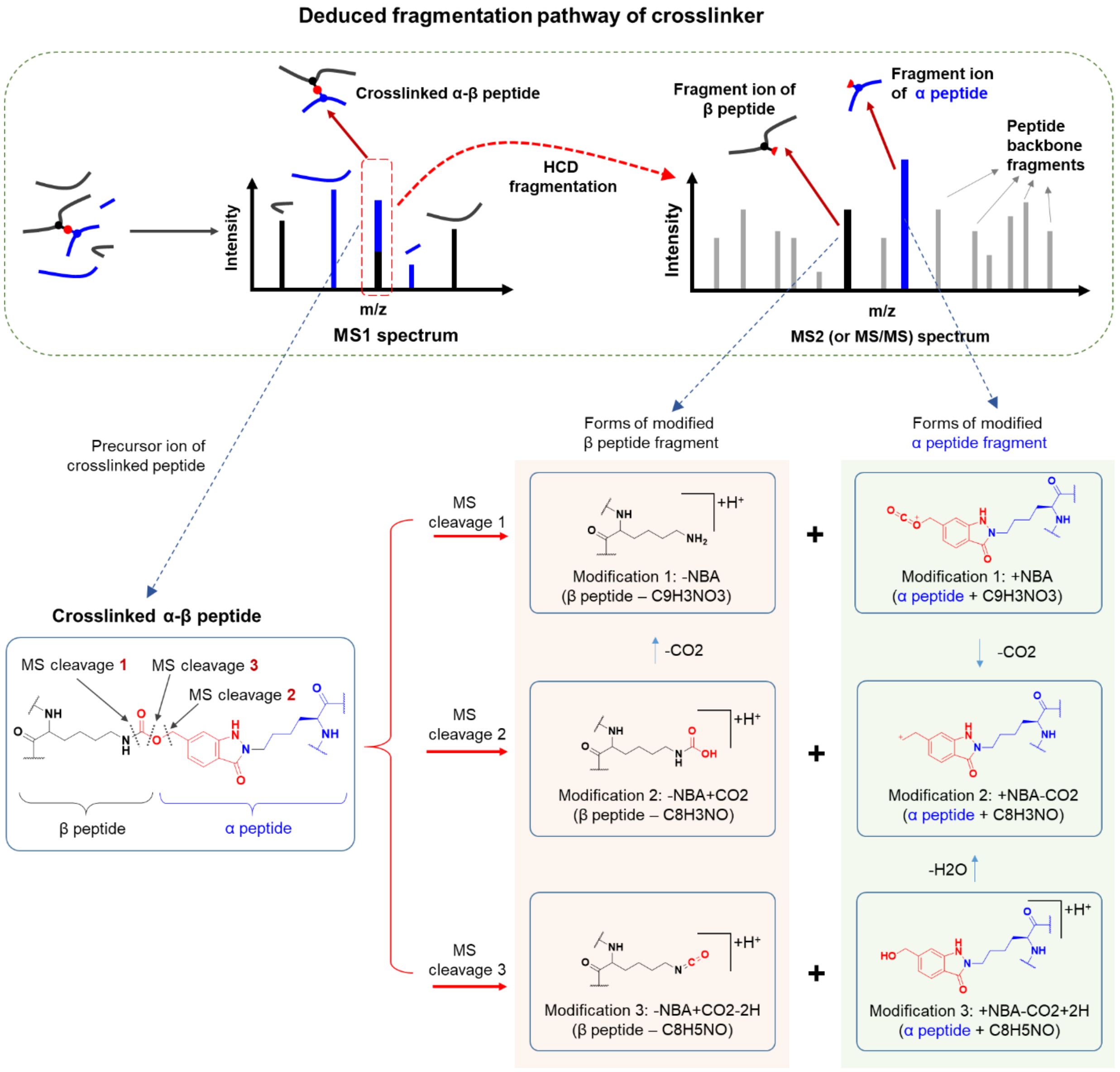

Supplementary Fig. 13. Deduced fragmentation of crosslinker in crosslinked α-β peptide. Upper panel: MS1 spectrum shows the precursor ions of crosslinked peptide and other co-eluted non-crosslinked peptides. The precursor ion of crosslinked peptide is then subjected to HCD fragmentation, which generates multiple forms of modified α/β peptide fragment ions as well as peptide backbone fragment ions shown in MS2 (or MS/MS) spectrum. Lower panel shows the structure and MS-cleavage bonds of the crosslinked peptide, as well as the modification types of α/β peptide fragments. Under the structure of each modification shows the arbitrary name and chemical formula. The protocol for preparing samples briefly as: *E. coli* cells expressing PatZ-L813o-NBAK, LplA-A138o-NBAK, or TmcA-R457o-NBAK were subjected to UV-irradiation, then His6-tag-based affinity purification, further SDS-PAGE separation, respectively. The bands with molecular weight higher than free enzyme in SDS-PAGE gel were cut and subjected to tryptic digestion and LC-MS/MS analysis.

Identified substrate proteins and their peptides crosslinked by LplA-A138o-NBAK in *E. coli* cells

Accession #	Gene name	Description (protein name)	Crosslinked peptide (red: crosslinked site)
P03024	galR	HTH-type transcriptional regulator GalR	MATIK(5)DVAR
P0A9B2	gapA	Glyceraldehyde-3-phosphate dehydrogenase A	K(116)VVMTGPSK
P60906	hisS	Histidine--tRNA ligase	K(371)QFARADK
P60716	lipA	Lipoyl synthase	MALIPVK(23)NVATEREALLR
P11349	narH	Respiratory nitrate reductase 1 beta chain	HYK(416)R
P0ADY7	rplP	50S ribosomal protein L16	MLQPK(5)R
P75864	rlmL	Ribosomal RNA large subunit methyltransferase K/L	K(405)FEKWAR *or* KFEK(408)WAR
P75864	rlmL	Ribosomal RNA large subunit methyltransferase K/L	DHLALMK(640)DLKRLLR *or* DHLALMKDLK(643)RLLR
P0A7M2	rpmB	50S ribosomal protein L28	SHALNATK(26)R
P10408	secA	Protein translocase subunit SecA	DPKQEYK(804)R
Q46814	xdhD	Probable hypoxanthine oxidase XdhD	MFGK(263)KMR *or* MFGKK(264)MR

Identified substrate proteins and their peptides crosslinked by PatZ-L813o-NBAK in *E. coli* cells

Accession #	Gene name	Description (protein name)	Crosslinked peptide (red: crosslinked site)
P08622	dnaJ	Chaperone protein DnaJ	AYK(26)R
P0A9Q1	arcA	Aerobic respiration control protein ArcA	K(188)MTGRE
P09372	grpE	Protein GrpE	EQK(7)TPE
P09372	grpE	Protein GrpE	TIRAAMVTVAK(194)AKA *or* TIRAAMVTVAKAK(196)A
P0AFG0	nusG	Transcription termination/antitermination protein NusG	GGQRRK(59)SE
P77488	dxs	1-deoxy-D-xylulose-5-phosphate synthase	EHIK(234)GMVVPGTLFE

Identified substrate proteins and their peptides crosslinked by TmcA-R457o-NBAK in *E. coli* cells

Accession #	Gene name	Description (protein name)	Crosslinked peptide (red: crosslinked site)
P36683	acnB	Aconitate hydratase B	K(571)NIFSGR
P21889	aspS	Aspartate--tRNA ligase	LK(123)YRYLDLRR
P0A9S3	gatD	Galactitol 1-phosphate 5-dehydrogenase	NAMPGK(341)VLLIP
P08956	hsdR	Type I restriction enzyme EcoKI endonuclease subunit	NAQEGVYLSK(669)GEQVER
P07813	leuS	Leucine--tRNA ligase	EK(34)YYCLSMLPYPSGR
P06720	melA	Alpha-galactosidase	K(55)LMDSAGASGKITCHTQQK
P06720	melA	Alpha-galactosidase	YEMFK(256)K
P15288	pepD	Cytosol non-specific dipeptidase	K(59)PATAGMENR

lplA(U138)-galR(K5) U = o-NBAK

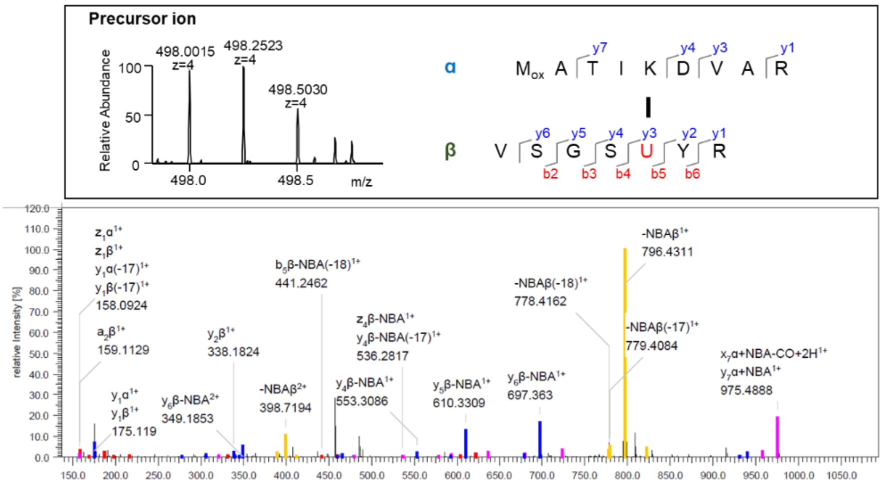

IplA(U138)-gapA(K116) U = o-NBAK

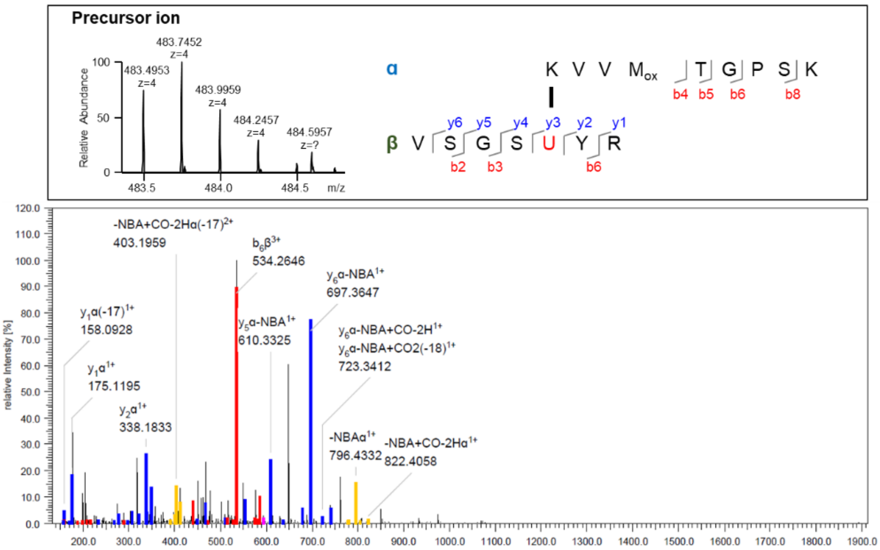

IplA(U138)-hisS(K371) U = o-NBAK

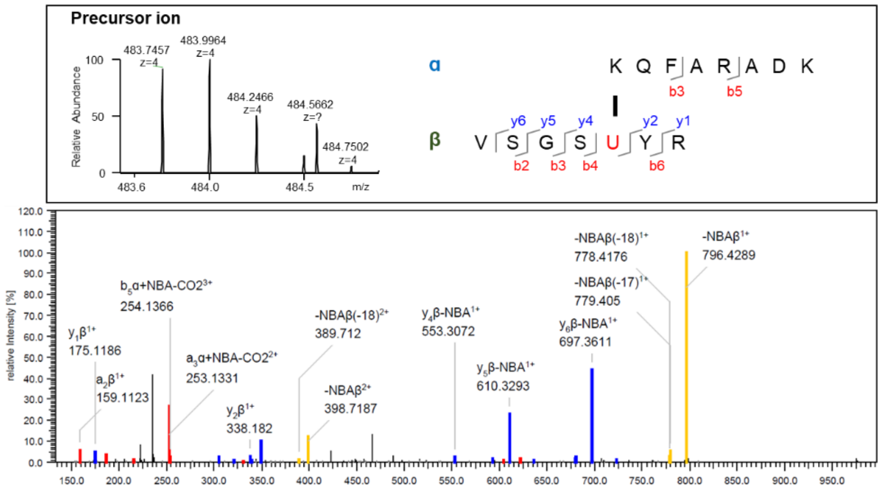

IplA(U138)-lipA(K23) U = o-NBAK

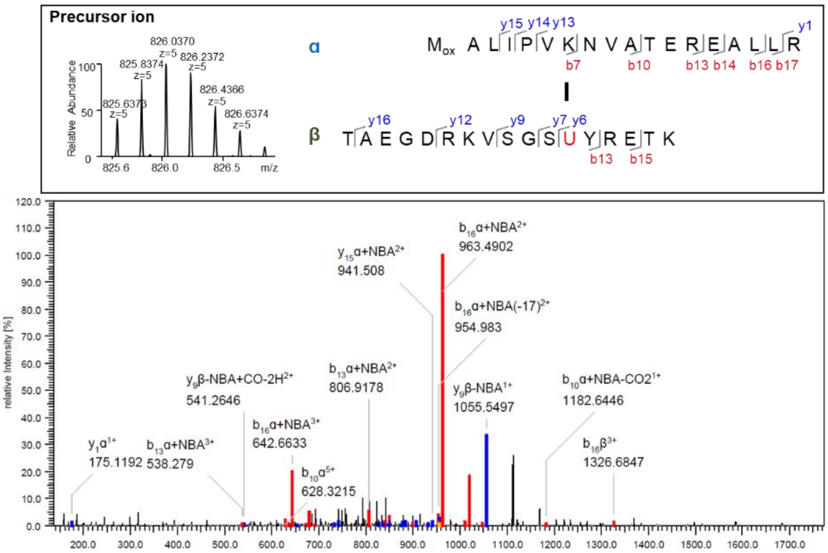

lplA(U138)-narH(K416) U = o-NBAK

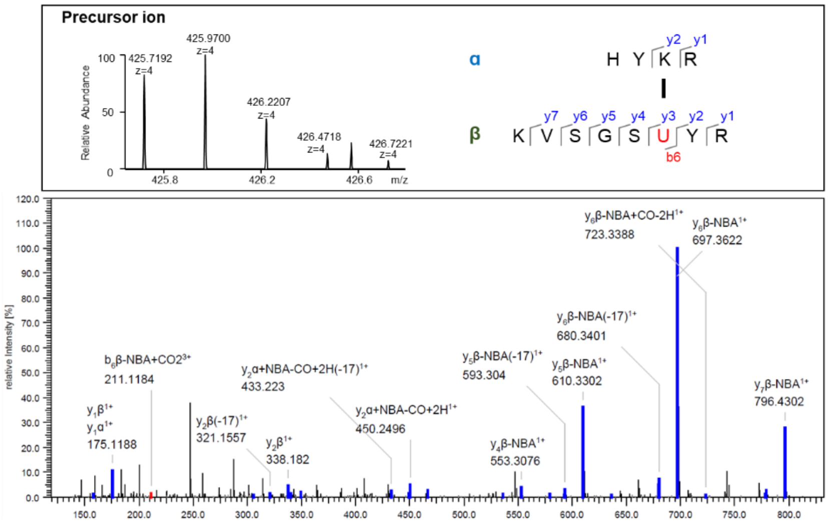

lplA(U138)-rplP(K5) U = o-NBAK

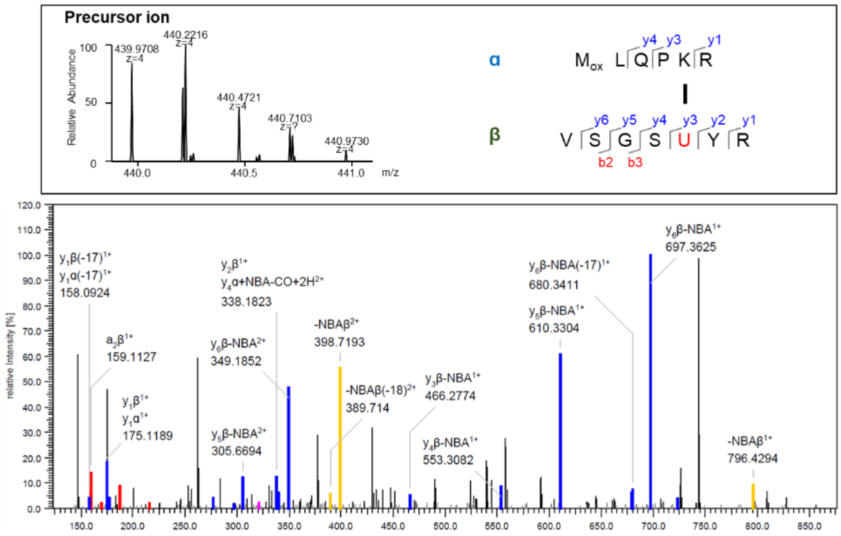

lplA(U138)-rlmL(K408) ***or*** **-rlmL(K405)** U = o-NBAK

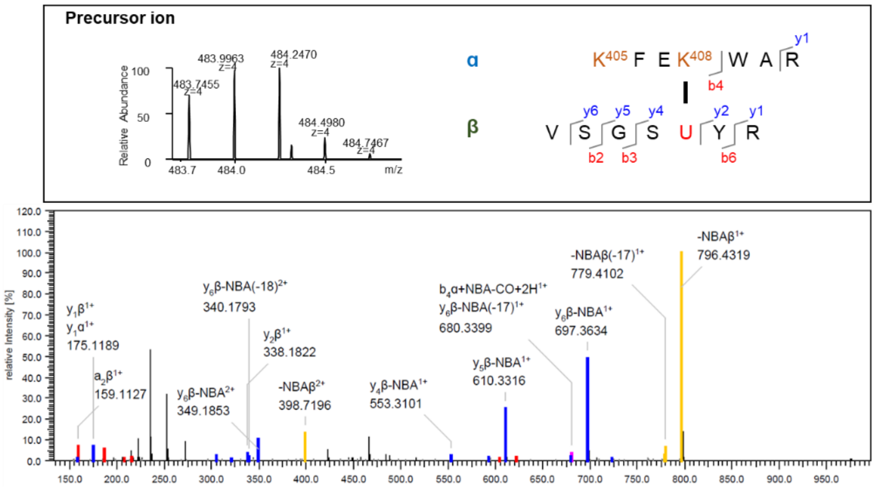

IplA(U138)-rlmL(K640) *or* -rlmL(K643)   U = o-NBAK

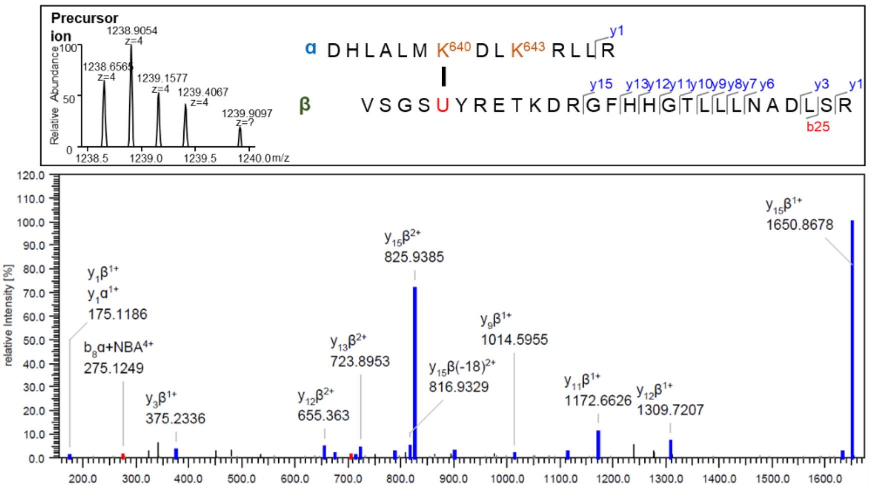

IplA(U138)-rpmB(K26)   U = o-NBAK

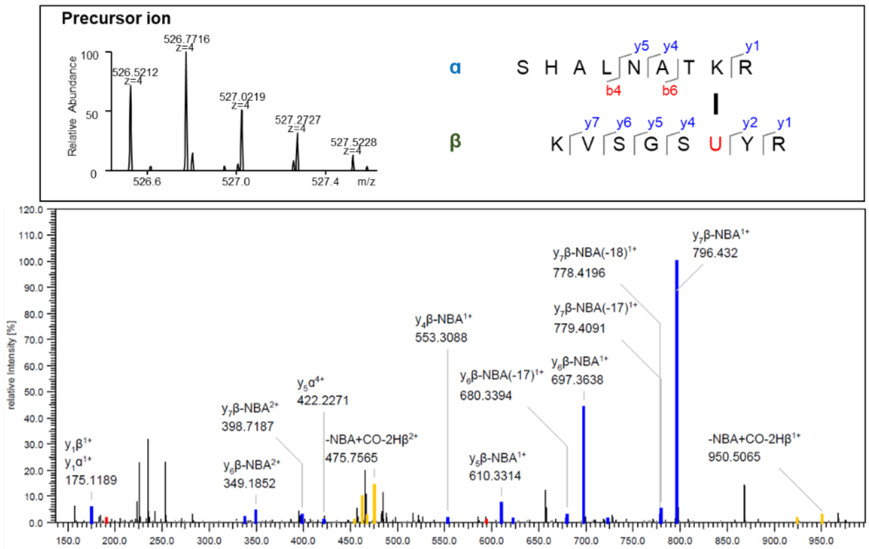

IplA(U138)-secA(K804)   U = o-NBAK

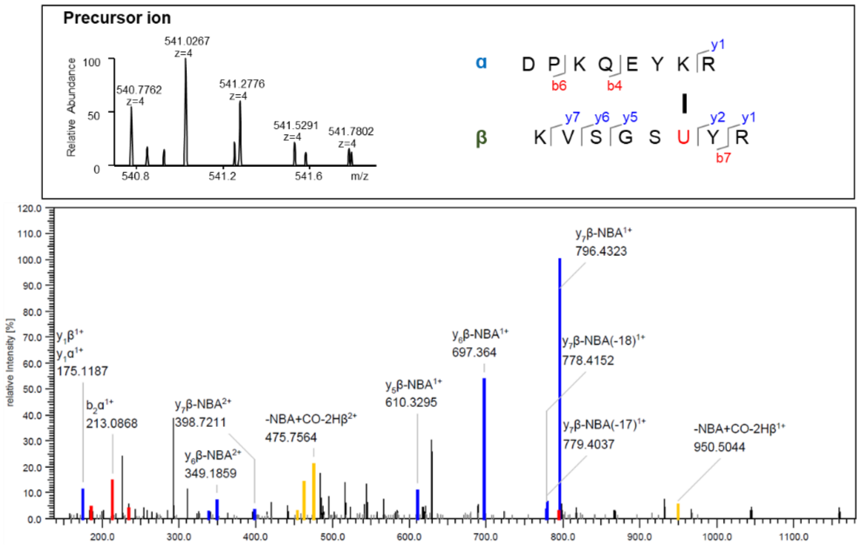

lplA(U138)-xdhD(K263) or -xdhD(K264) U = o-NBAK

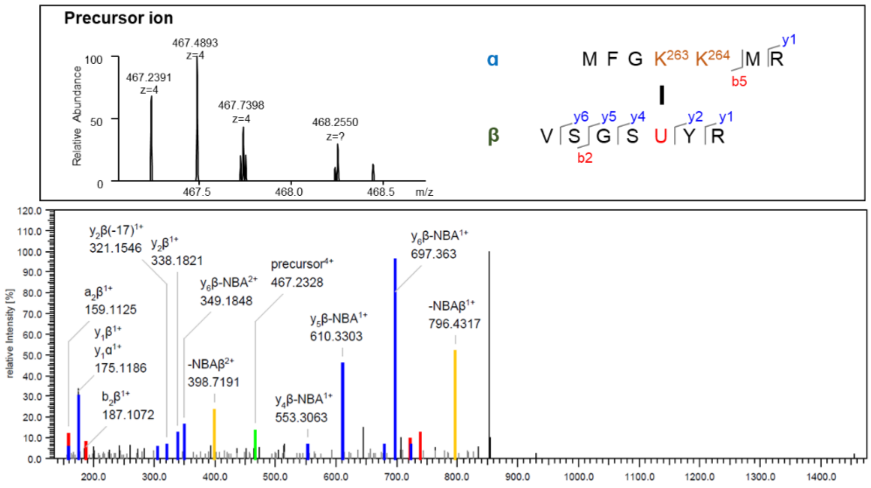

patZ(U813)-dnaJ(K26) U = o-NBAK

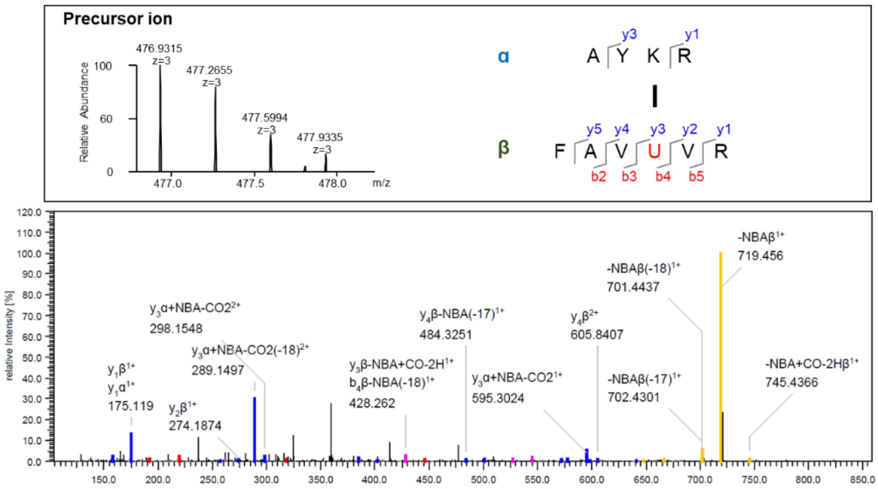

patZ(U813)-arcA(K188) U = o-NBAK

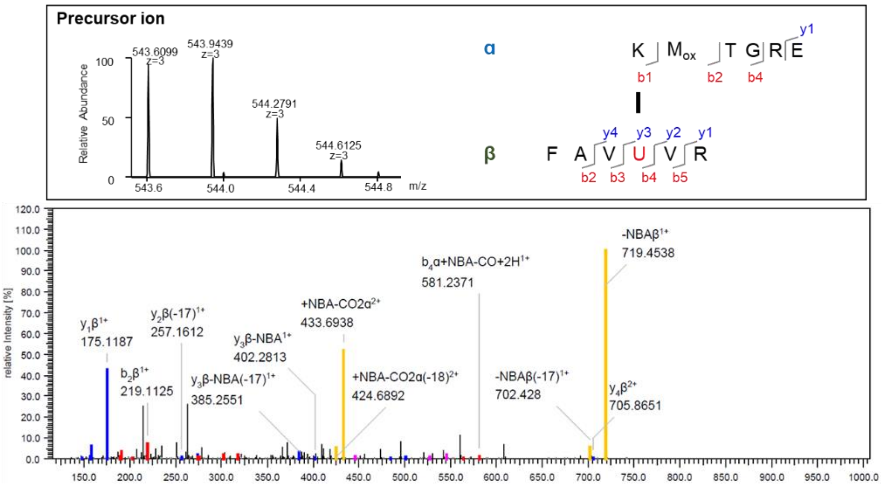

patZ(U813)-grpE(K7) U = o-NBAK

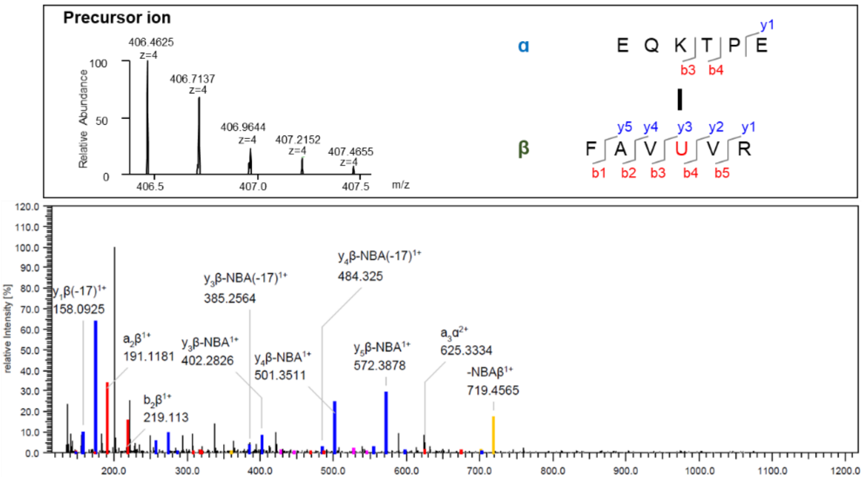

patZ(U813)-grpE(K194) *or* **-grpE(K196)** U = o-NBAK

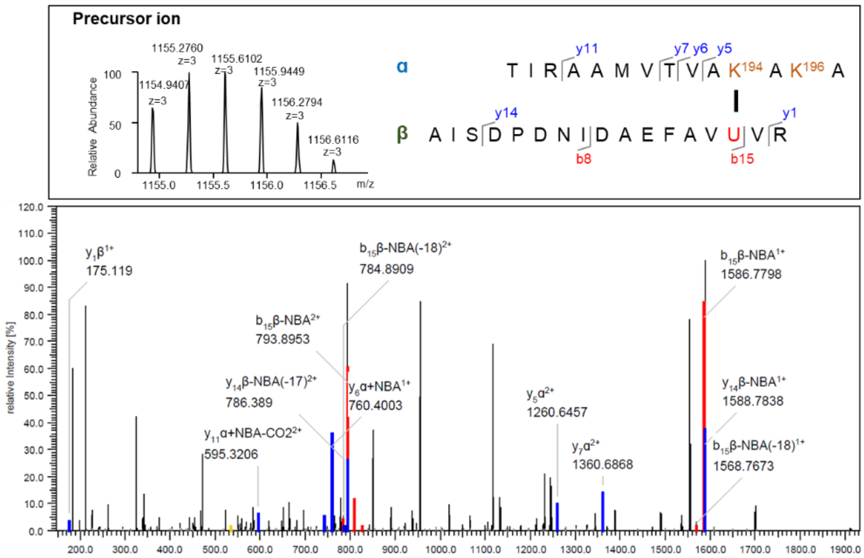

patZ(U813)-nusG(K59) U = o-NBAK

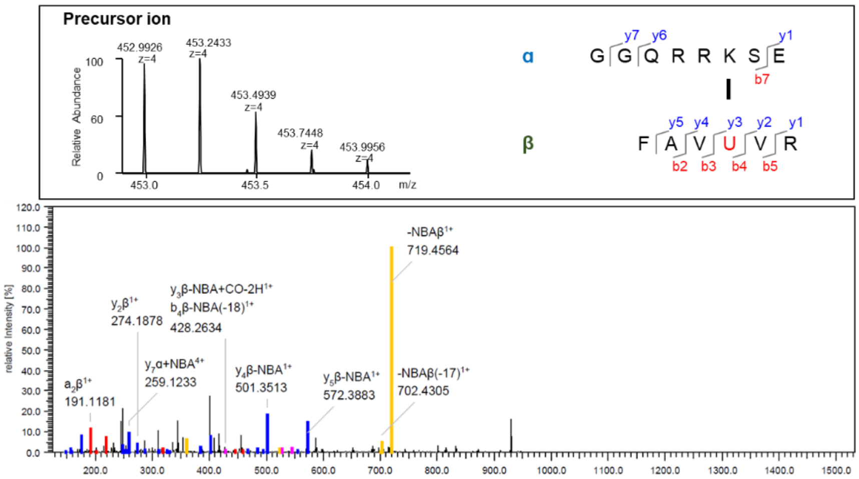

patZ(U813)-dxs(K234) U = o-NBAK

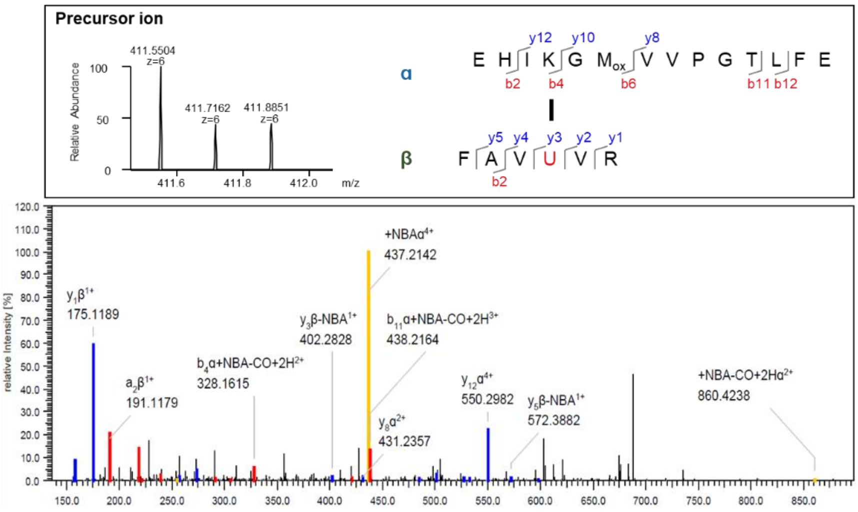

tmcA(U457)-acnB(K571) U = o-NBAK

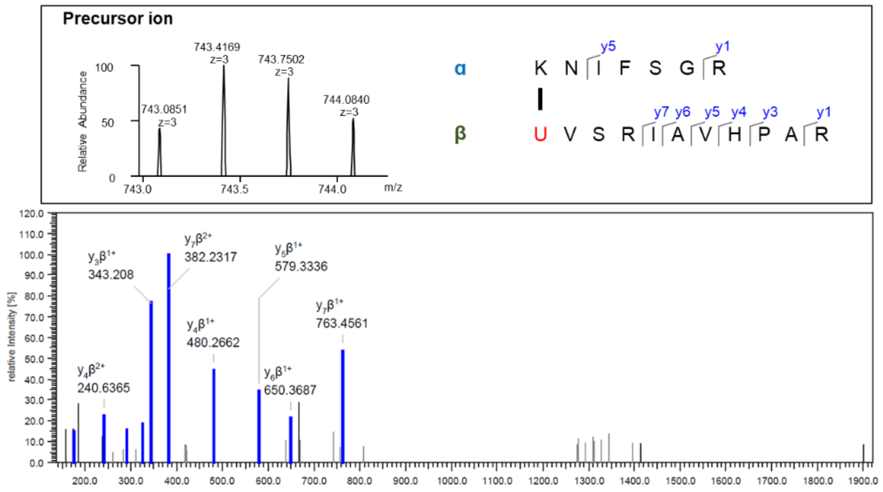

tmcA(U457)-aspS(K123) U = o-NBAK

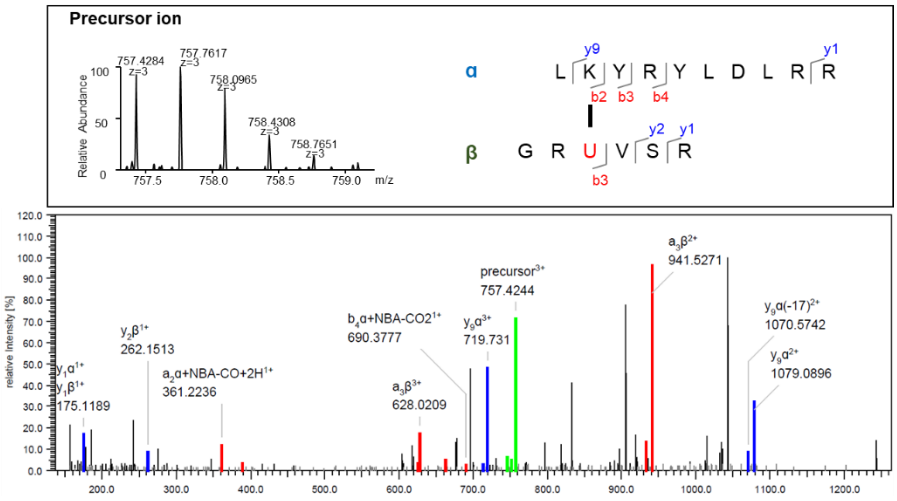

tmcA(U457)-gatD(K341) U = o-NBAK

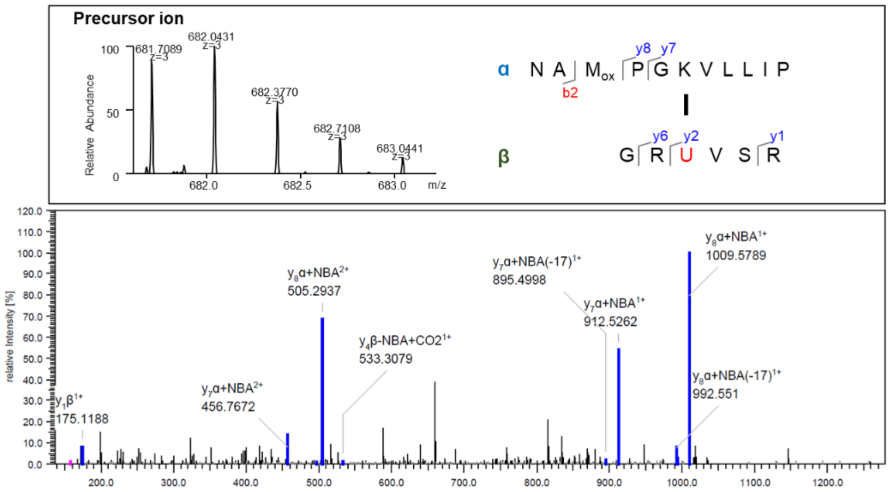

tmcA(U457)-hsdR(K669) U = o-NBAK

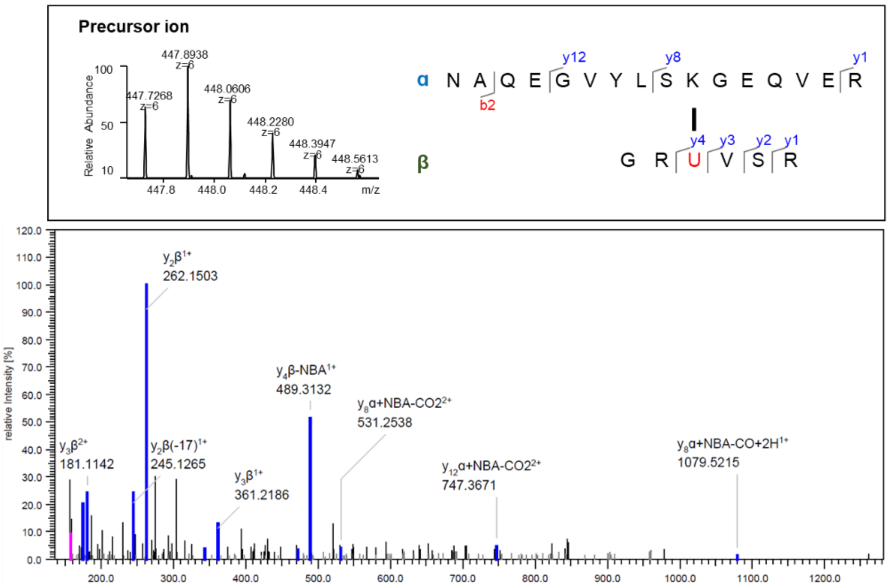

tmcA(U457)-leuS(K34) U = o-NBAK

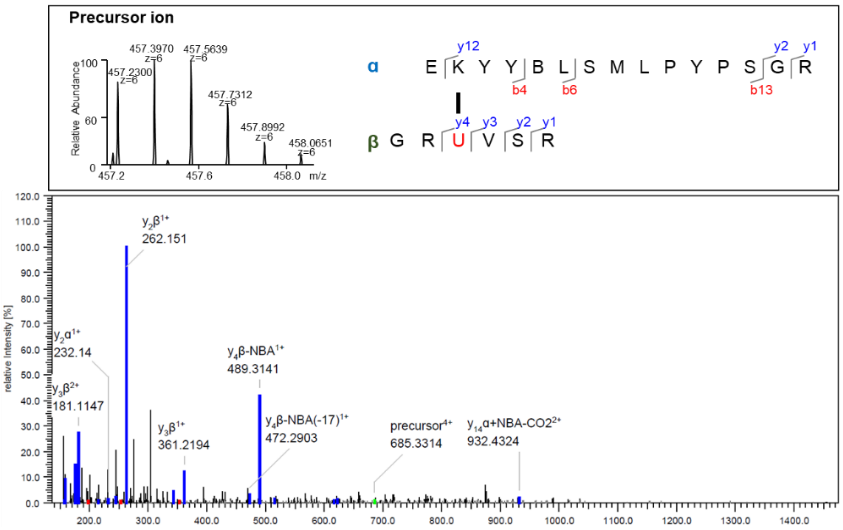

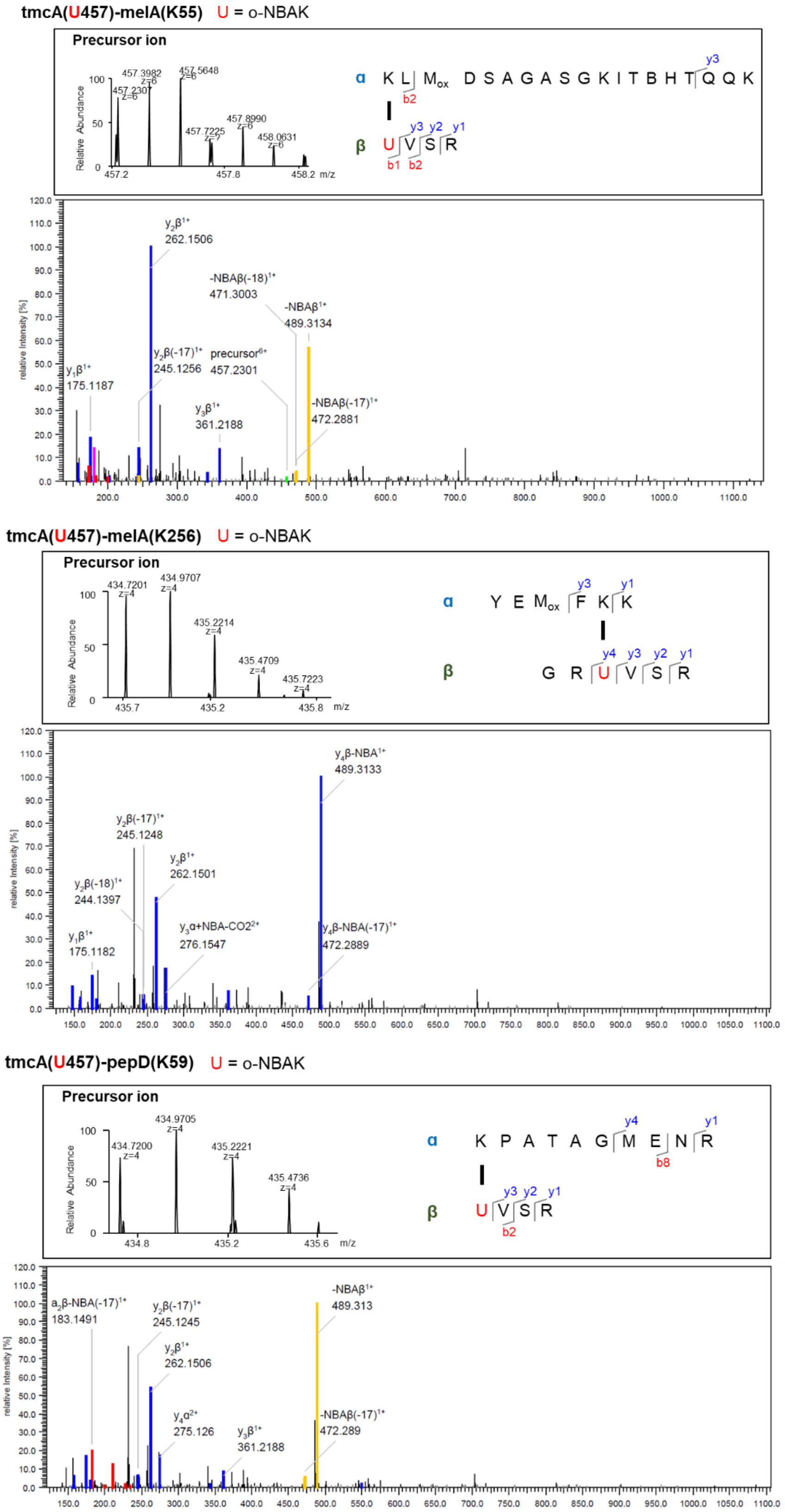

Supplementary Fig. 14. Identified substrate proteins and their peptides crosslinked by PatZ-L813o-NBAK, LplA-A138o-NBAK or TmcA-R457o-NBAK mutants in *E. coli* cells. Tables show crosslinked peptides/sites from the cell lysate samples directly. In each graph, upper panel shows the MS1 spectrum of the precursor ion and sequence of crosslinked peptides, and lower panel shows the annotated MS/MS spectrum. The crosslinked proteins are shown in gene names.

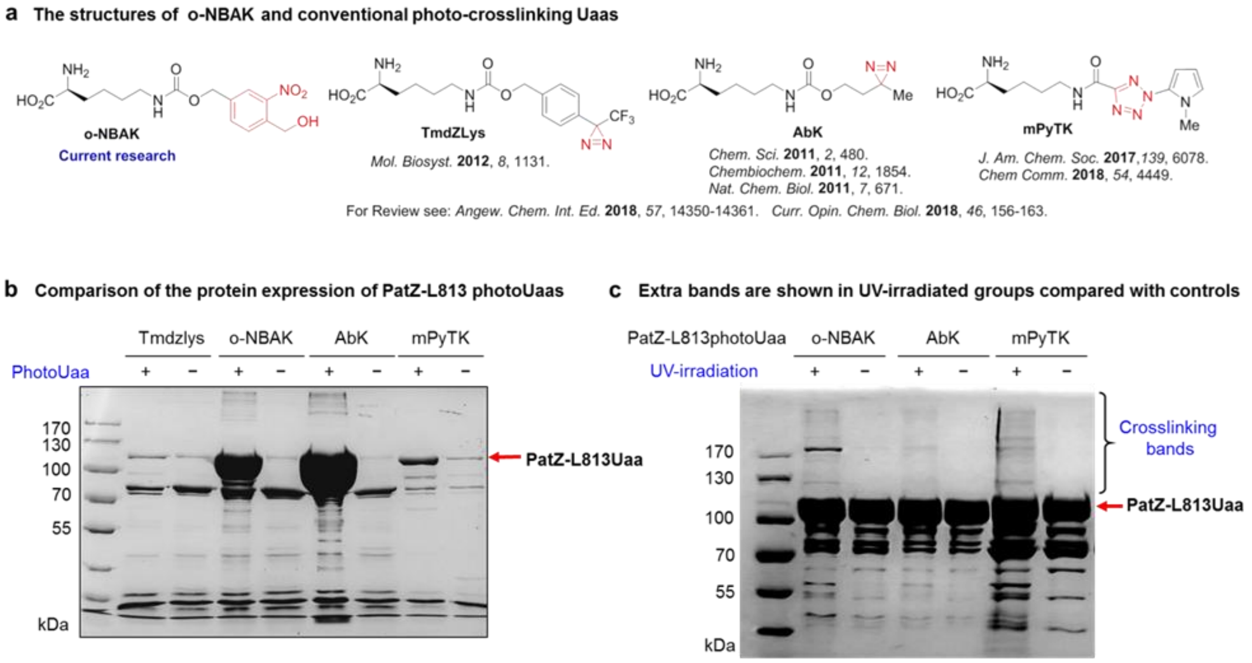

Supplementary Fig. 15. Genetic incorporation of photoUaas into PatZ and *in situ* crosslinking. **a**, Structures of o-NBAK, TmdZlys, AbK and mPyTK. **b**, Comparison of protein expression of PatZ-L813photoUaas. PatZ-L813photoUaas were expressed with or without photoUaas in *E. coli*, purified based on His6-tag, analyzed with SDS-PAGE and coomassie brilliant blue staining. **c**, Sliver staining of samples from *in situ* ±UV-irradiation treatment (20 min), cell lysis, His6-tag affinity-enrichment and SDS-PAGE analysis. The loading amounts of samples were adjusted so that PatZ-L813photoUaas were at uniform level.

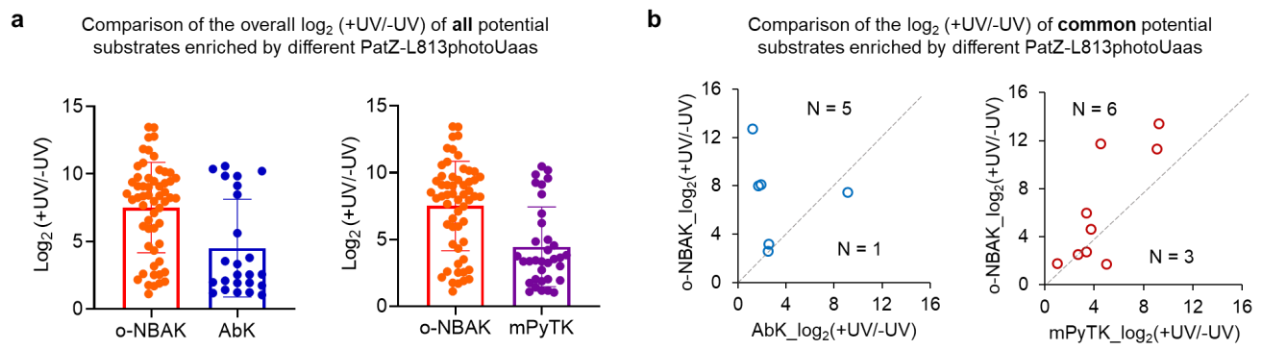

Supplementary Fig. 16. Quantitative comparison of enrichment efficiencies between PatZ-L813photoUaas. a, Column graphs comparing the averaged enrichment ratio (log$_2$ (+UV/-UV)) of potential substrates significantly enriched by PatZ-L813o-NBAK with those by PatZ-L813AbK or PatZ-L813mPyTK. +UV: UV-irradiation group; -UV: control group without UV-treatment. Error bars represent standard deviations. n=57 proteins enriched PatZ-L813o-NBAK, 24 proteins enriched PatZ-L813AbK, 34 proteins enriched PatZ-L813mPyTK. **b,** Comparison of enrichment ratios of potential substrates commonly enriched by PatZ-L813o-NBAK and PatZ-L813AbK, or commonly enriched by PatZ-L813o-NBAK and PatZ-L813mPyTK. The log$_2$(+UV/-UV) values of enriched proteins were averaged from technique triplicates. Source data are provided as a Source Data file.

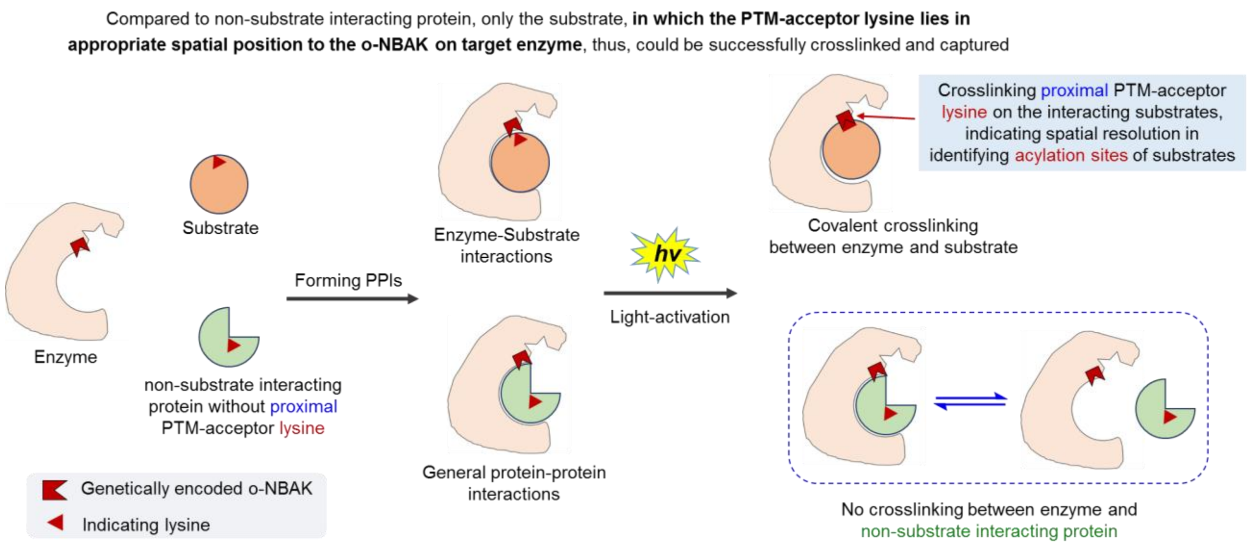

Supplementary Fig. 17. Schematics of spatial resolution of our direct capturing strategy. Enzyme only captures the certain lysine residues of the substrates with appropriate distances or suitable angles, thus realizing spatial resolution in identifying acylation sites of substrates.

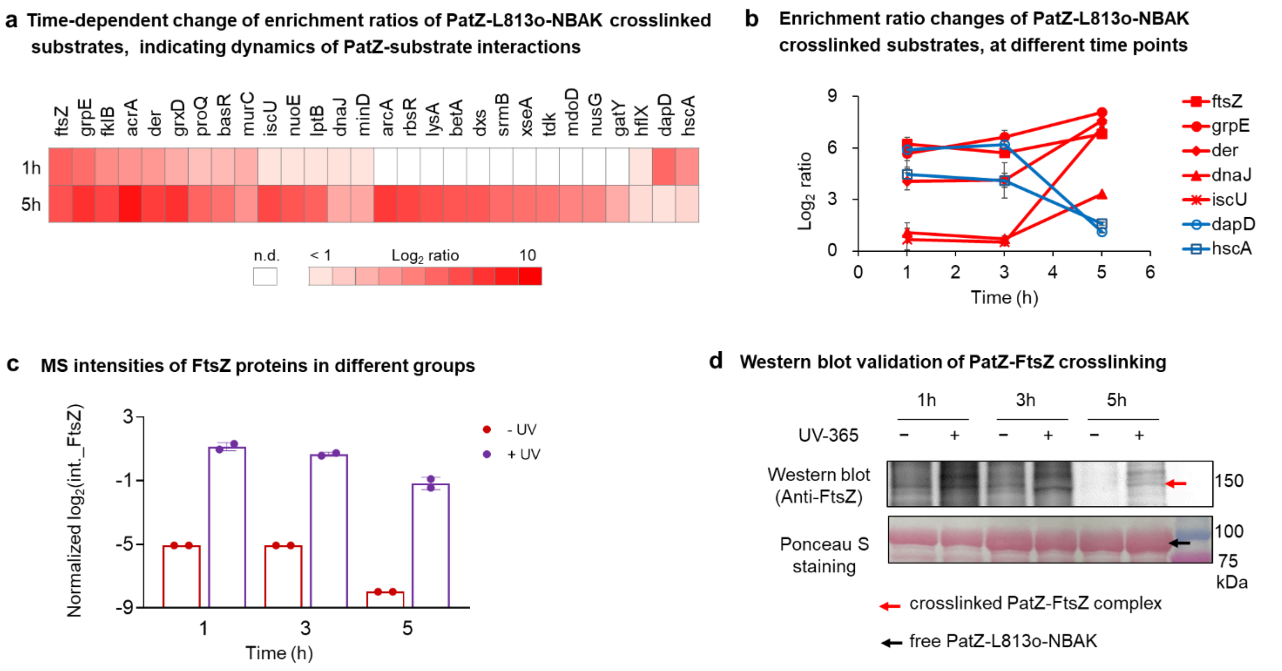

Supplementary Fig. 18. Time-resolved profiling of PatZ-L813o-NBAK crosslinking substrates. a, Time-dependent change of enrichment ratios of 28 PatZ-L813o-NBAK crosslinked candidate substrates reported in manuscript. Heatmap shows enrichment ratios (shown in log2-transformed +UV/-UV ratios) averaged from technique duplicates. n.d. = not detected. **b**, Changes of enrichment ratios (i.e. Log_2(+UV/-UV)) of selected candidate substrates at different time points of UV-crosslinking. The candidate substrates in (**a**) and (**b**) are shown in gene names. **c,** Column graph shows the normalized log2-transformed MS intensities of FtsZ protein in -UV and +UV groups across different time points. **d**, Validation of crosslinking between PatZ-L813o-NBAK and FtsZ protein at different time points. Western blot was performed with anti-FtsZ antibody. Unreacted PatZ-L813o-NBAK proteins visualized with Ponceau S staining was used as protein loading control. Source data are provided as a Source Data file.

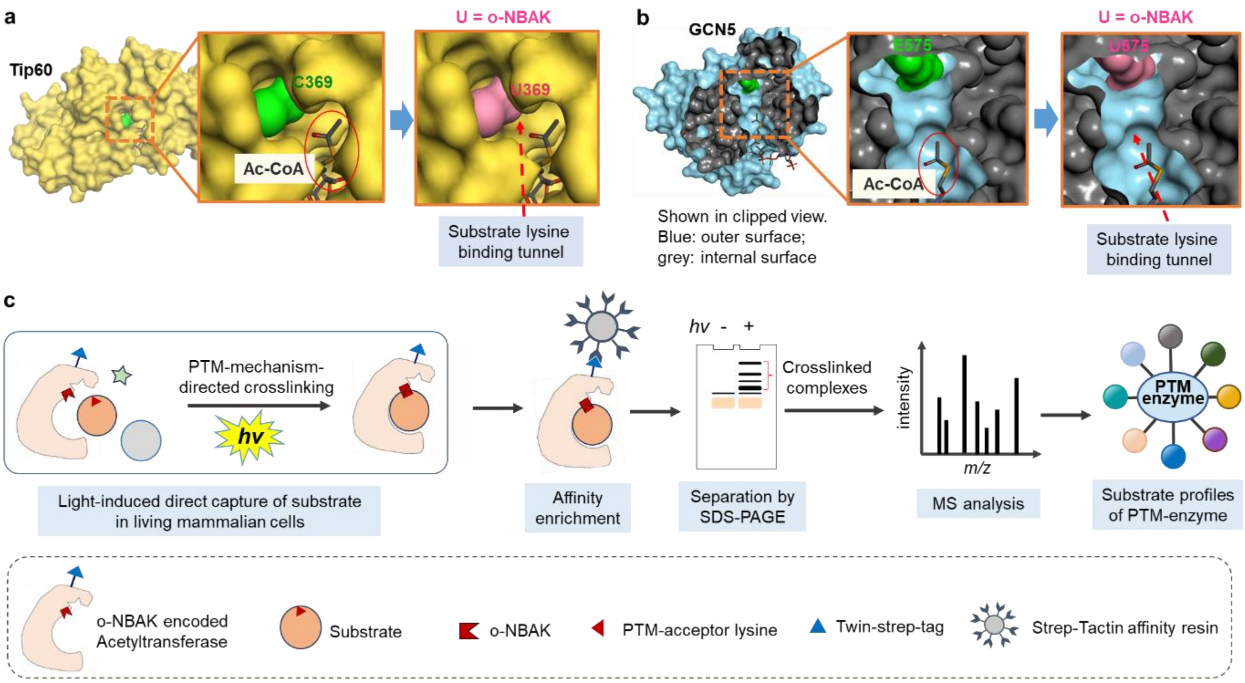

Supplementary Fig. 19. Schematics of global profiling substrates of acetyltransferases Tip60 or GCN5 via direct capturing strategy in living cells. a, Surface mode of Tip60 (PDB ID: 2OU2). **b**, Surface mode of GCN5 (PDB ID: 1Z4R, catalytic domain). The enlarged areas show the substrate lysine binding tunnel and the relative spatial arrangement of residues (in green surface mode) to be mutated to o-NBAK. Note that, the pink-colored residues only indicate the incorporation sites of the o-NBAK (U), rather than their structures. In GCN5, Since the E575 residue is covered by other residues in front view, the clipped view is shown for better visualization (Note that E575 still has access to the lysine binding pocket). The locations of the acetyl groups in the bound Ac-CoA (in sticks) are shown by red circles, indicating the substrate lysine molecules binding tunnel. **c**, Workflow of substrate profiling by direct capturing approach based on Tip60-C369o-NBAK or GCN5-E575o-NBAK. Human 293F cells expressing C-terminally twin-strep-tagged Tip60-C369o-NBAK or GCN5-E575o-NBAK were subjected to 365 nm UV light irradiation, using cells without UV irradiation (-UV) as controls. After cell lysis and affinity purification, the eluate was further resolved by SDS-PAGE. The bands with higher molecular weight than free Tip60-C369o-NBAK or GCN5-E575o-NBAK molecules in SDS-PAGE gel were excised and further subjected to trypsin digestion and LC-MS/MS analysis.

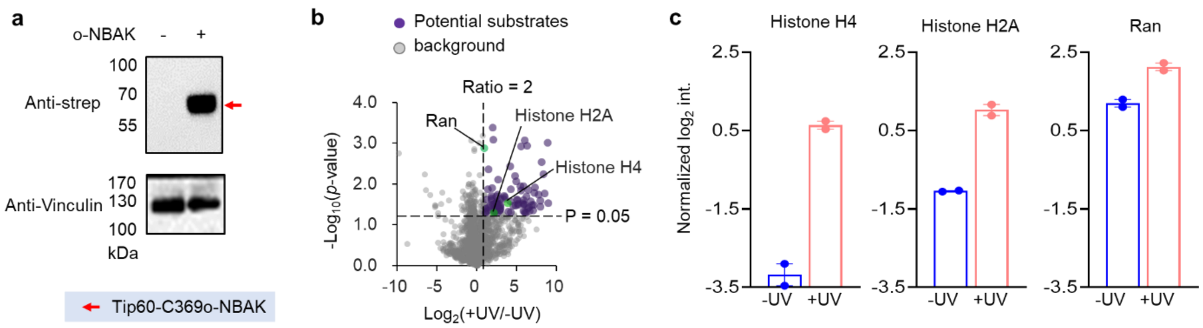

Supplementary Fig. 20. Profiling substrates of acetyltransferase Tip60 via direct capturing strategy in living cells. a, Western blot analysis of Tip60-C369o-NBAK expression in the absence or presence of o-NBAK. Vinculin was used the loading control protein. **b,** Volcano plot displaying proteins significantly enriched (+UV/-UV ratio > 2, p-value < 0.05, purple dots, considered as candidate substrates) by Tip60-C369o-NBAK from duplicate experiments, with histone H4, H2A, and Ran proteins (ratio = 1.9, p-value = 0.001) showing in green dots. Statistical tests are unpaired, two-sided two-sided Student's t-test. **c**, Normalized log2-transformed MS intensities of known Tip60 substrates histone H4, H2A, and Ran proteins. Error bars represent standard errors of mean (s.e.m.). Source data are provided as a Source Data file.

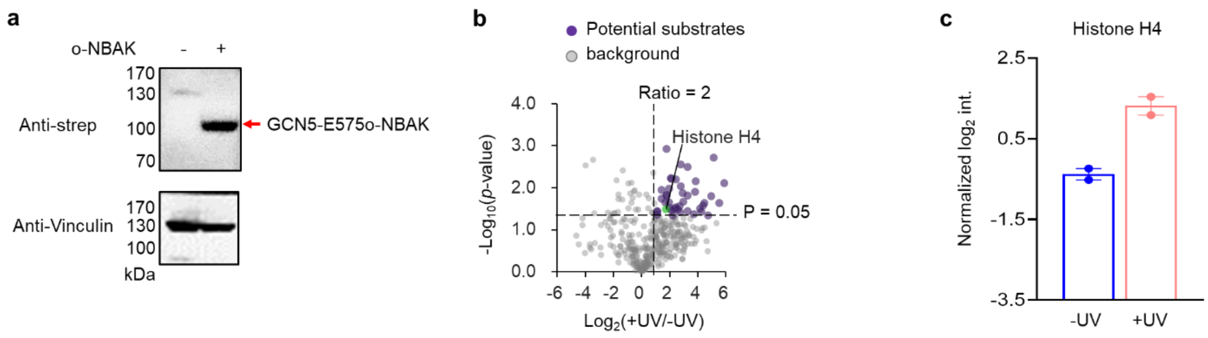

Supplementary Fig. 21. Profiling substrates of acetyltransferase GCN5 via direct capturing strategy in living cells. a, Western blot analysis of GCN5-E575o-NBAK expression in the absence or presence of o-NBAK. Vinculin was used the loading control protein. **b,** Volcano plot displaying proteins significantly enriched (+UV/-UV ratio > 2, p-value < 0.05, purple dots, considered as candidate substrates) by GCN5-E575o-NBAK from duplicate experiments, with histone H4 protein showing in green dot. Statistical tests are unpaired, two-sided two-sided Student's t-test. **c**, Normalized log2-transformed MS intensities of known GCN5 substrate histone H4 protein. Error bars represent standard errors of mean (s.e.m.). Source data are provided as a Source Data file.

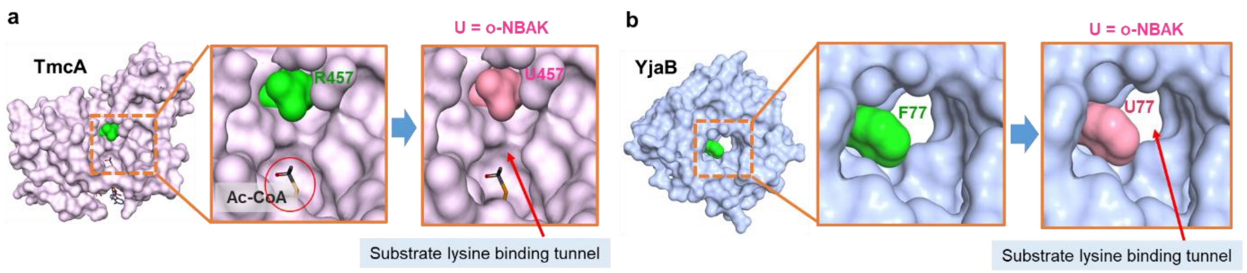

Supplementary Fig. 22. Structures of acyltransferases TmcA or YjaB. a, Surface mode of the catalytic domain of TmcA (PDB ID: 2ZPA). The enlarged area shows the substrate lysine binding pocket and relative spatial arrangement of R457 (in green surface mode) to be mutated to o-NBAK. Note that, the pink-colored residue only indicates the incorporation site of the o-NBAK (U), rather than its structure. The location of the acetyl groups in the bound Ac-CoA (shown in sticks) is red circled, which indicates the substrate lysine molecule binding tunnel. **b**, Surface mode of YjaB (PDB ID: 2KCW). The enlarged area shows the lysine binding pocket as indicated from structural alignment with the homologs PatZ and YiaC (the result of alignment is not shown). Note that, the pink-colored residue only indicates the incorporation site of the o-NBAK (U), rather than its structure. The F77 to be mutated to o-NBAK is shown in green surface mode.

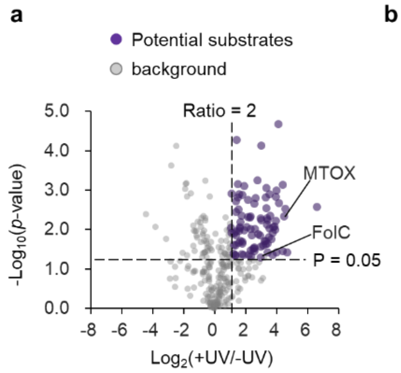

b

	Detection of Khib-containing peptides in tested groups		
TmcA-catalyzed Khib sites	Experimental group	Control group 1	Control group 2
	TmcA +	TmcA −	TmcA −
	Hib-CoA +	Hib-CoA +	Hib-CoA −
	Substrate +	Substrate +	Substrate +
MTOX-358Khib	Detected	not detected	not detected
FolC-318Khib	Detected	not detected	not detected

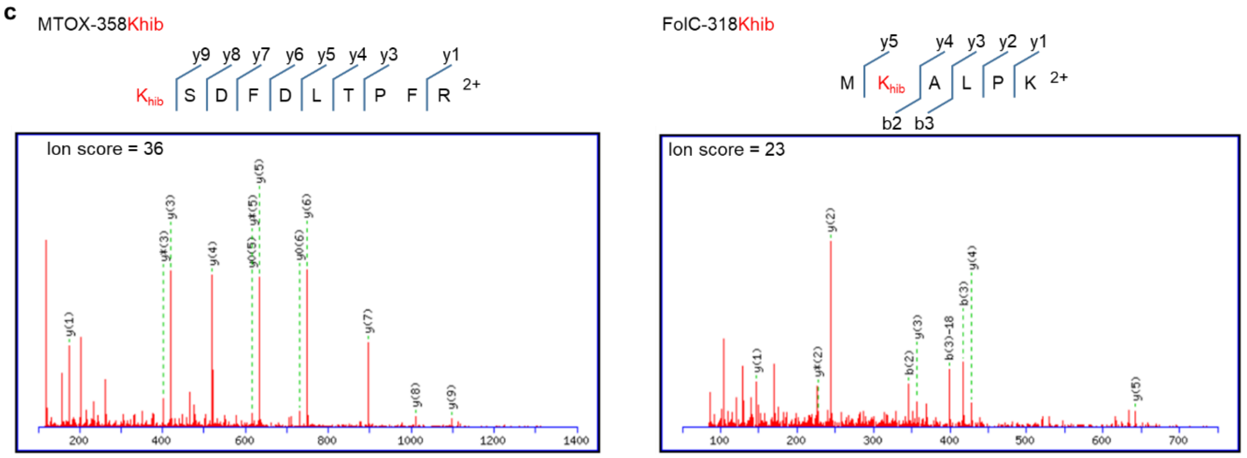

Supplementary Fig. 23. Global substrate profiling of TmcA enzyme and validation of substrate 2-hydroxyisobutyrylation by TmcA enzyme. a, Volcano plot displaying proteins significantly enriched (+UV/-UV ratio > 2, p-value < 0.05, purple dots, considered as candidate substrates) by TmcA-R457o-NBAK from three replicates. Statistical tests are unpaired, two-sided two-sided Student's t-test. The experiments were performed with the same workflow as that of PatZ substrate profiling reported in manuscript. Briefly, *E. coli* cells expressing C-terminally His6-tagged TmcA-R457o-NBAK with or without UV-irradiation were subjected to His6-tag-based affinity purification, SDS-PAGE separation. The bands with higher molecular weight than free enzyme in SDS-PAGE gel were excised and further subjected to trypsin digestion and LC-MS/MS analysis for protein identification and quantification. **b**, Summary of wild type TmcA-catalyzed lysine 2-hydroxyisobutyrylation (Khib) sites of candidate substrates. Purified candidate substrate protein was incubated with wild type TmcA and Hib-CoA, as experimental group. Sample without TmcA was used as control for non-enzymatic acetylation (control 1) and sample only containing substrate was used as control for intrinsic acetylation (control 2). **c**, Annotated MS/MS spectra of peptides bearing MTOX-358Khib and FolC-318Khib.

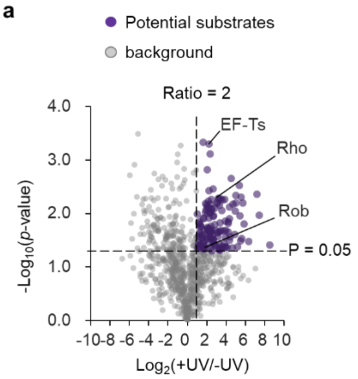

b

YjaB-catalyzed Kac sites	Normalized ratios of MS intensities		
	Experimental group	Control group 1	Control group 2
	YjaB +	YjaB −	YjaB −
	Ac-CoA +	Ac-CoA +	Ac-CoA −
	Substrate +	Substrate +	Substrate +
EF-Ts-104Kac	1.9	1	Not detected
EF-Ts-222Kac	2.1	1.3	1
Rho-258Kac	2.7	1.7	1
Rho-402Kac	99	50	1
Rob-35Kac	11.8	7.7	1

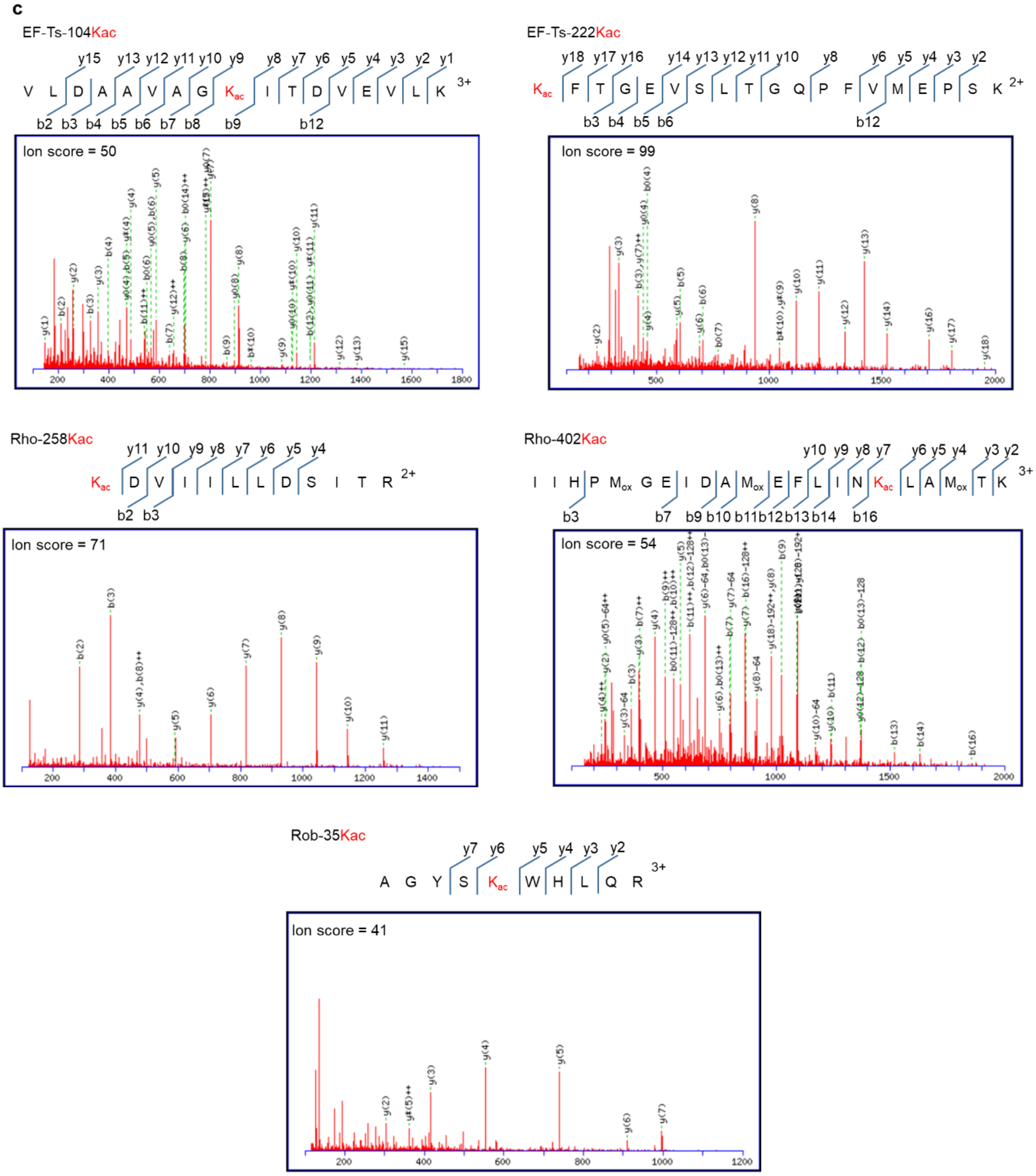

Supplementary Fig. 24 Global substrate profiling of YjaB enzyme and substrate validation. a, Volcano plot displaying proteins significantly enriched (+UV/-UV ratio > 2, p-value < 0.05, purple dots, considered

as candidate substrate) by YjaB-F77o-NBAK from two replicates. Statistical tests are unpaired, two-sided two-sided Student’s t-test. *E. coli* cells expressing C-terminally His6-tagged YjaB-F77o-NBAK with or without UV-irradiation were subjected to His6-tag-based affinity purification, SDS-PAGE separation. The bands with higher molecular weight than free enzyme in SDS-PAGE gel were excised and subjected to trypsin digestion and LC-MS/MS analysis. **b**, Summary of wild type YjaB-catalyzed lysine acetylation (Kac) sites of candidate substrates and the normalized ratios of the Kac levels in three tested groups. Purified candidate substrate protein was incubated with wild type YjaB and Ac-CoA, as experimental group. Sample without YjaB was used as control for non-enzymatic acetylation (control 1) and sample only containing substrate was used as control for intrinsic acetylation (control 2) The normalized ratios were calculated as follows: for each tested group, the peak areas of all peptides containing the same acetylation site were added up; then, the ratios of total peak areas from different groups were calculated, and normalized using peak areas of 3-4 unmodified peptides (as loading control). **c,** Annotated MS/MS spectra are shown for acetylated peptides.

a

YiaC-catalyzed lysine acylation sites	Normalized ratios of MS intensities		
	Experimental group	Control group 1	Control group 2
	YiaC +	YiaC −	YiaC −
	Acyl-CoA +	Acyl-CoA +	Acyl-CoA −
	Substrate +	Substrate +	Substrate +
RPL14-40Khib	1.9	1	Not detected
RPL14-66Kpr/67Kpr	1.5	1	Not detected
RPL14-40Kmal	3.2	1	Not detected
RPL14-111Kmal	2.1	1	Not detected
RPL14-114Kmal	1.7	1	Not detected
GrpE-194Kpr	2.4	1	Not detected

b

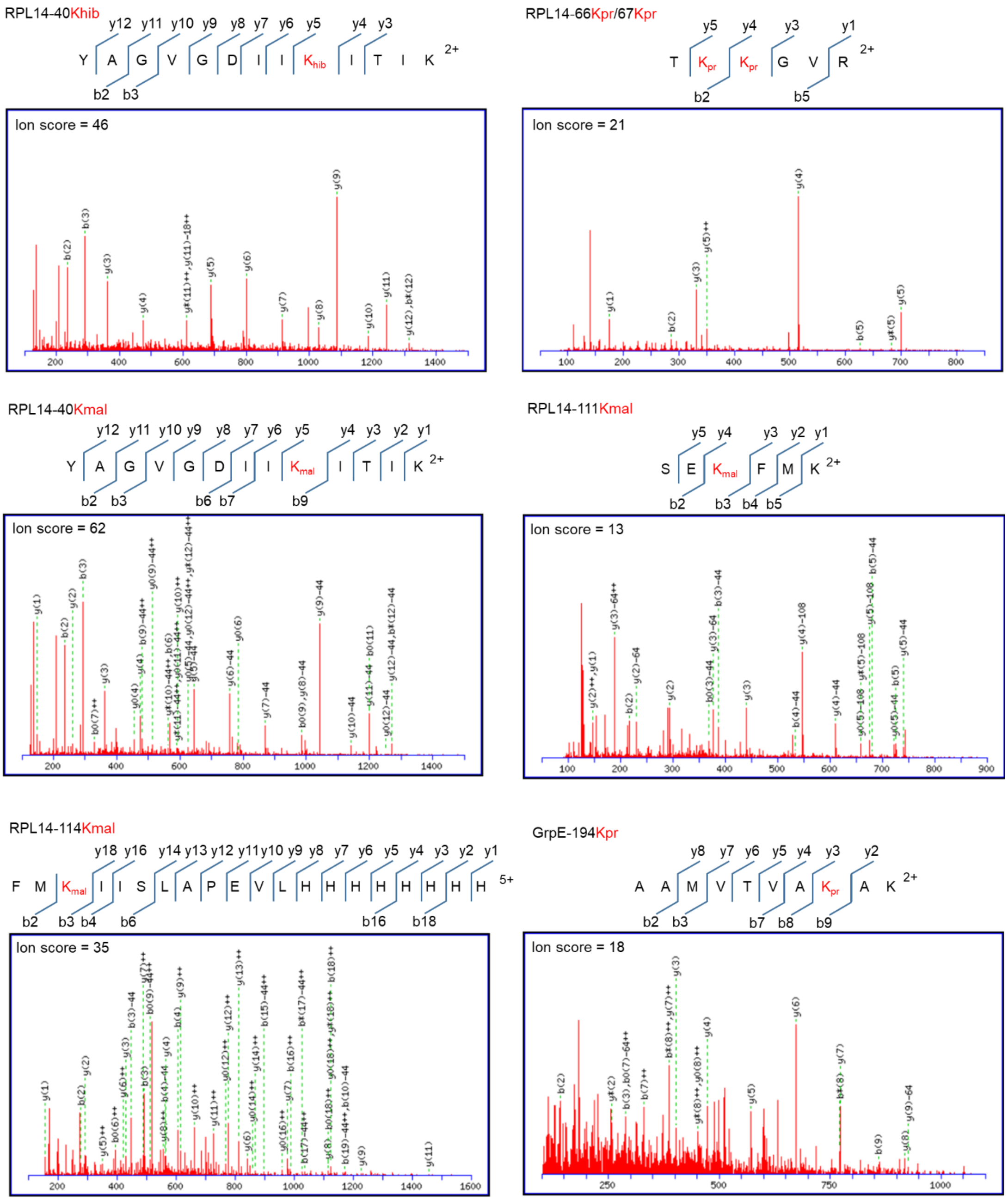

Supplementary Fig. 25. YiaC-catalyzed acylation on RPL14 and GrpE proteins. a, Summary of wild type YiaC-catalyzed lysine acylation (Khib, Kpr, and Kmal) sites of candidate substrates and the normalized ratios of the acylation levels in three tested groups. Purified candidate substrate protein was incubated with

wild type YiaC and Acyl-CoA, as experimental group. Sample without YiaC was used as control for non-enzymatic acylation (control 1) and sample only containing substrate was used as control for intrinsic acylation (control 2). The normalized ratios were calculated as follows: for each tested group, the peak areas of all peptides containing the same acylation site were added up; then, the ratios of total peak areas from different groups were calculated, and normalized using peak areas of 3-4 unmodified peptides (as loading control). **b**, Annotated MS/MS spectra of YiaC-catalyzed acylation peptides from RPL14 and GrpE proteins.

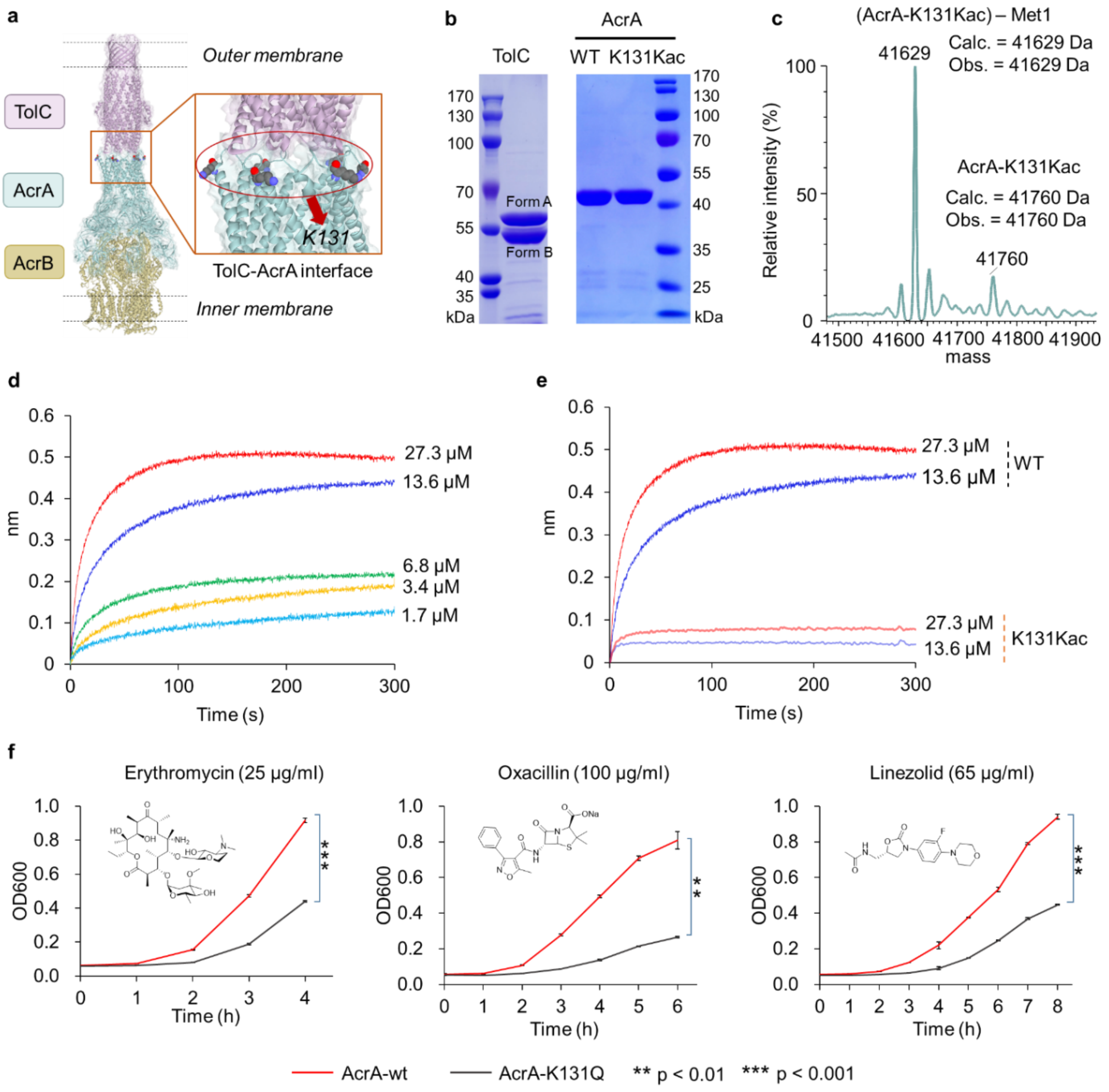

Supplementary Fig. 26 Acetylation on K131 of AcrA affects binding with TolC. a, Structure of the AcrAB-TolC multidrug efflux pump (PDB ID: 5V5S), with the interface between TolC trimer and AcrA hexamer enlarged. K131 in each AcrA monomer is shown in CPK forms. **b**, SDS-PAGE analysis of purified TolC, AcrA-wt and AcrA-K131Kac. Two protein forms (form A and form B) of TolC were obtained, which was the same as reported. **c**, Deconvoluted mass spectrum of AcrA-K131Kac indicating successful incorporation of Kac into AcrA. **d**, BLI analysis of interaction between TolC (4 μM) and serial concentrations of AcrA-wt. Higher signal indicates stronger binding. **e**, Comparison of BLI signals of AcrA-wt and AcrA-K131Kac. Minimal signal changes of AcrA-K131Kac indicate rather weak interaction with TolC compared to AcrA-wt. **f**, Growth curves of *E. coli* BW25113*ΔacrA* cells harboring either *acrA-wt* gene, or *acrA-K131Q* gene in liquid LB culture media containing antibiotics as indicated in graphs. OD600 value at each time point was averaged from triplicate experiments. Error bars represent standard errors of mean (s.e.m.). Source data are provided as a Source Data file.

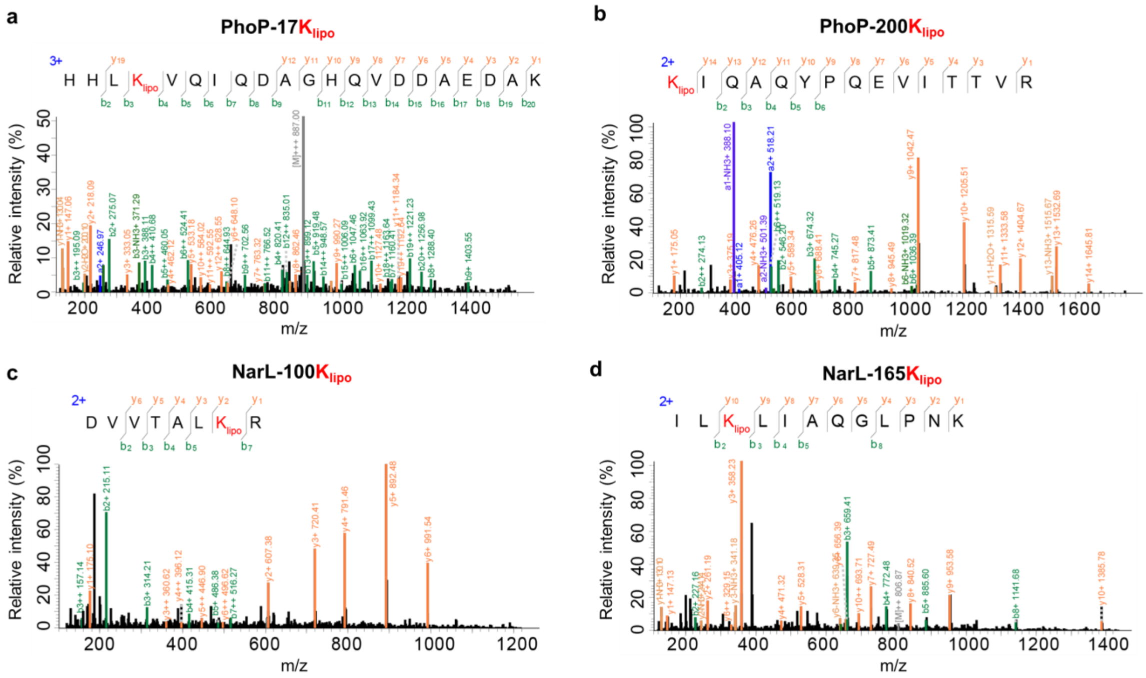

Supplementary Fig. 27 Validation of substrate lipoylation by LplA enzyme. a-d, Annotated MS/MS spectra of lipoylated peptides from PhoP (**a**, PhoP-17Klipo, and **b,** PhoP-200Klipo) and NarL (**c**, NarL-100Klipo, and **d**, NarL-165Klipo).

Supplementary Note 1. Guideline for the selection of appropriate photoUaa incorporation sites.

Step 1: Identify the binding pocket or enzyme active site tunnel for substrate lysine.

Retrieve structure information for the enzyme of interest (EOI) by download crystal structure from PDB database, homology-modeling, or using AlphaFold-predicted structure (https://alphafold.com/). **II)**. Perform literature survey to obtain information on the location of the substrate lysine binding pocket or tunnel. If there is no such information reported, molecular docking could be performed using lysine-protein (or protein-protein) docking software (e.g. HADDOCK, Z-DOCK, GRAMM-X, RosettaDock etc.) to display possible enzyme-substrate recognition mode and the pocket (or tunnel) where the enzyme accommodates substrate lysine. Note that many possible binding modes would be generated for a given substrate from docking analysis, and the true one does not necessarily have highest docking score. In this regard, we recommend to perform the docking study for several canonical or well-known substrates (and/or with different docking software) and choose the shared docking site(s) as the candidate pocket(s).

Step 2: Selecting proper sites to incorporate o-NBAK Uaa, which includes two situations:

I). The 3D structure of EOI and GNAT domain share certain similarity. Over several thousands of members are found in GNAT domain superfamily from all life domain (from prokaryote to eukaryote). Although their primary amino acid sequences share poor similarity, the 3D structures of GNAT fold are highly conserved.[7,8] In addition, albeit not belonged to the GNAT domain superfamily, the catalytic domains of p300/CBP family and MYST family acetyltransferases also share high structural homology to GNAT fold regarding to the binding site of substrate lysine and Ac-CoA.[9,10] In this regard, the model enzyme (e.g. PatZ) we chose in this work is highly representative. Therefore, for EOIs that share structural similarity to GNAT domain, we think choosing those sites with similar spatial location to L813 of PatZ would be a good start point to determine the optimal residues to be replaced with o-NBAK Uaa. This concept is demonstrated by the substrate profiling experiments on *E. coli* acyltransferases YiaC, TmcA and YjaB as well as human acetyltransferases GCN5 and Tip60.

II). The 3D structure of EOI is irrelevant to GNAT domain (such as LplA in our manuscript). In this case, the rules of thumb for selecting residues to be replaced by o-NBAK include: choosing residues lining the wall of the binding pocket accommodating the substrate Lys, with 1-2 residues away or reasonable distance (e.g. 5-15 Å) from the substrate Lys. If o-NBAK is situated immediately to the substrate Lys, the long side chain would cause a steric hindrance to impede the entrance of Lys into pocket. In addition, choosing the site half-buried in the binding pocket while allowing o-NBA group to project towards substrate Lys. Installation of o-NBAK on a fully exposed site would cause non-specific crosslinking, while on a fully buried site would perturb the structural stability. Moreover, avoiding those sites that are essential to maintain structural integrity of enzyme or mediate enzyme-substrate recognition.

Supplemental Methods

General Information

Solvents and chemicals were purchased from commercial sources and used directly without further purification. Ac-CoA (Cat. No. TX20016) was purchased from Shanghai Yingxin laboratory equipment Co., Ltd (Shanghai, China). Propionyl-CoA (Cat. No. P5397) and malonyl-CoA (Cat. No. M4263) were purchased from Sigma-Aldrich. 2-hydroxyisobutyryl CoA,[11] unnatural amino acid o-NBAK,[12] AbK,[13] mPyTK[14] and TmdZLys[15] were synthesized as previously described. BS2G crosslinker (Cat. No. 21610), BCA assay kit (Cat. No. 23225), Silver Stain Kit (Cat. No. 24612) and FreeStyle™ 293-F cells (Cat. No. R79007) was purchased from Thermo Scientific. Protease Inhibitor Cocktail was purchased from Sigma Aldrich (Cat. No. 4693159001). Ni-NTA agarose resin for was purchased from Qiagen (Cat. No. 130210) or Smart-Lifesciences (ChangZhou, China). MagStrep type3 XT beads (Cat. No. 2-4090-002) was purchased from IBA-Lifesciences. SMM 293-TII Expression Medium (Cat. No. M293TII) was purchased from Sino Biological, Inc. (China). Primer synthesis and sequencing were conducted by GENEWIZ (Suzhou, China). Complement *E. coli* cells DH10B and BL21 (DE3) were purchased from Biofeng. Isopropyl-β-d-thiogalactoside (IPTG) was from Sinopharm (Shanghai, China). Polyethylenimine (PEI) (Cat. No. 24885-2) was purchased from Polysciences, Inc.. DNA polymerase and restriction enzymes were from NEB (Beijing, China). Site-directed mutagenesis kit (Cat. No. 11003ES10), and Thrombin (Cat. No. 20402ES03) were obtained from Yeasen (Shanghai, China). RIPA lysis buffer (Cat. No. P0013C) was purchased from Beyotime (China). Mouse anti-His tag monoclonal antibodies (Cat. No. HRP-66005), mouse anti-vinculin monoclonal antibody (Cat. No. 66305-1-Ig) and HRP-conjugated goat anti-mouse antibody (Cat. No. SA00001-1) were purchased from Proteintech (Shanghai, China). Acetylated-lysine mouse monoclonal antibody (Cat. No. 9681) and HRP-conjugated goat anti-rabbit antibody (Cat. No. 7074) were purchased from CST (Shanghai, China), and rabbit polyclonal FtsZ antibody (Cat. No. CSB-PA359270HA01EGX) was purchased from CUSABIO. Mouse Anti-Strep-Tag II Monoclonal Antibody (Cat. No. ABT2230) was purchased from Abbkine (China). The plasmid pNEU-hMbPylRS-4×U6M15 (Cat. No. 105830) was purchased from Addgene. Light-induced reactions were conducted with ZF-7A 16-W 365-nm UV lamp (Shanghai Gucun Optic Instrument Factory, Shanghai, China) as light source.

1. Protein expression and purification

1.1 Construction of plasmid and gene-deletion strain

The DNA fragment of each wild type protein was amplified by PCR using total genomic DNA of *E. coli* BL21(DE3) strain as templates. TmcA-R457TAG was mutated from pBR322-*TmcA* (a gift from Kai Zhang) using overlapping PCR. The PCR product containing a sequence coding His-tag at the C-terminal and a restriction enzyme recognition sites (coding two amino acids T and S) following the initiation codon was cloned into the predigested pTAK vector by recombinase to obtain the wild-type protein expression plasmid. The *acrA* gene was further amplified from pTAK-*acrA* plasmid and cloned into pET24a plasmid. For the construction of mutant plasmids, the wild type plasmid was set as the template and the site-directed mutation method or overlapping PCR was used according to the manufacturer’s protocol.

To generate vectors for protein expression in mammalian cells, Tip60-C369TAG or GCN5-E575TAG was mutated

from pcDNA3.1-HA-GCN5 or pCMV-Flag-Tip60 (gifts from Jiemin Wong) using overlapping PCR and amplified and introduced into pcDNA3.1 plasmid backbone_with 4×U6-tRNA[M15]. A twin-Strep-tag was introduced at the C-terminus of Tip60-C369TAG or GCN5-E575TAG.

E. coli BW25113*ΔacrA* strain was constructed by Forhigh Biotech (Hangzhou, China) based on reported method.[16] Briefly, genomic DNA of *E. coli* BW25113 strain using DOKDO-Prep bacterial genomic DNA purification extraction kit. Up- and downstream homologous arms of *acrA* were amplified from *E. coli* BW25113 genomic DNA. pCas vector was transformed into *E. coli* BW25113 cells, and the λ-red recombinase was induced by L-arabinose. The guide RNA and editing template were transformed into Cas9 BW25113 electrocompetent cells by electroporation and cultured on kanamycin /chloramphenicol agar plate. The positive clones were identified by PCR, and grow on LB agar plate containing kanamycin and 0.1 mM IPTG to eliminate gRNA plasmid, and then grow in LB agar plate at 42 °C overnight to eliminate pCas vector.

1.2 Protein sequences used in this study

For numbering of protein sequences in main text, the two residue in position 2 and 3 at N-terminal which are coded by the restriction enzyme recognition sites are ignored, such that the number of sequence is the same as those recorded in UniProt database.

PatZ-wt-His8:

MTSSQRGLEALLRPKSIAVIGASMKPNRAGYLMMRNLLAGGFNGPVLPVTPAWKAVLGVLAWPDIASLPFTPD
LAVLCTNASRNLALMEELGEKGCKTCIILSAPASQHEDLRACALRHNMRLLGPNSLGLLAPWQGLNASFSPVPI
KRGKLAFISQSAAVSNTILDWAQQREMGFSYFIALGDSLDIDVDELLDYLARDSKTSAILLYLEQLSDARRFVSA
ARSASRNKPILVIKSGRSPAAQRLLNTTAGMDPAWDAAIQRAGLLRVQDTHELFSAVETLSHMRPLRGDRLMIIS
NGAAPAALALDALWSRNGKLATPSEETCQKLRDALPEHVAVSNPLDLRDDASSEHYVKTLDILLHSQDFDALM
VIHSPSAAAPATESAQVLIEAVKHHPRSKYVSLLTNWCGEHSSQEARRLFSEAGLPTYRTPEGTITAFMHMVEYR
RNQKQLRETPALPSNLTSNTAEAHLLLQQAIAEGATSLDTHEVQPILQAYGMNTLPTWIASDSTEAVHIAEQIGY
PVALKLRSPDIPHKSEVQGVMLYLRTANEVQQAANAIFDRVKMTWPQARVHGLLVQSMANRAGAQELRVVVE
HDPVFGPLIMLGEGGVEWRPEDQAVVALPPLNMNLARYLVIQGIKSKKIRARSALRPLDVAGLSQLLVQVSNLI
VDCPEIQRLDIHPLLASGSEFTALDVTLDIAPFEGDNESRLAVRPYPHQLEEWVELKNGERCLFRPILPEDEPQLQ
QFISRVTKEDLYYRYFSEINEFTHEDLANMTQIDYDREMAFVAVRRIDQTEEILGVTRAISDPDNIDAEFAVLVRS
DLKGLGLGRRLMEKLITYTRDHGLQRLNGITMPNNRGMVALARKLGFNVDIQLEEGIVGLTLNLAQREESHHH
HHHHH.

LplA-wt-His8:

MTSSTLRLLISDSYDPWFNLAVEECIFRQMPATQRVLFLWRNADTVVIGRAQNPWKECNTRRMEEDNVRLARR
SSGGGAVFHDLGNTCFTFMAGKPEYDKTISTSIVLNALNALGVSAEASGRNDLVVKTAEGDRKVSGSAYRETK
DRGFHHGTLLLNADLSRLANYLNPDKKKLAAKGITSVRSRVTNLTELLPGITHEQVCEAITKAFFAHYGERVEA
EIISPDKTPDLPNFAETFARQSSWEWNFGQAPAFSHLLDERFSWGGVELHFDVEKGHITRAQVFTDSLNPAPLEA
LAGRLQGCLYRADMLQQECEALLVDFPDQEKELRKLSTWMAGAVRHHHHHHHH.

YiaC-wt-His8:

MTSIREAQRSELPAILELWLESTTWGHPFIKANYWRECIPLVRDAYLANAQNWVWEEDSKLLGFVSIMEGRFL

AAMFVAPKAVRRGIGKALMQYVQQRHPHLMLEVYQKNQPAIDFYQAQGFHIVDCAWQDETQLPTWIMSWPV
VQTLHHHHHHHH.

IscU-wt-His6

MTSAYSEKVIDHYENPRNVGSFDNNDENVGSGMVGAPACGDVMKLQIKVNDEGIIEDARFKTYGCGSAIASSS
LVTEWVKGKSLDEAQAIKNTDIAEELELPPVKIHCSILAEDAIKAAIADYKSKREAKHHHHHH.

DnaJ-wt-His6:

MTSAKQDYYEILGVSKTAEEHEIKKAYKRLAMKYHPDRNQGDKEAEAKFKEIKEAYEVLTDSQKRAAYDQYG
HAAFEQGGMGGGGFGGGADFSDIFGDVFGDIFGGGRGRQRAARGADLRYNMELTLEEAVRGVTKEIRIPTLEE
CDVCHGSGAKPGTQPQTCPTCHGSGQVQMRQGFFAVQQTCPHCQGRGTLIKDPCNKCHGHGRVERSKTLSVK
IPAGVDTGDRIRLAGEGEAGEHGAPAGDLYVQVQVKQHPIFEREGNNLYCEVPINFAMAALGGEIEVPTLDGRV
KLKVPGETQTGKLFRMRGKGVKSVRGGAQGDLLCRVVVETPVGLNEKQKQLLQELQESFGGPTGEHNSPRSK
SFFDGVKKFFDDLTRHHHHHH.

AcrA-wt-His6:

MTSNKNRGFTPLAVVLMLSGSLALTGCDDKQAQQGGQQMPAVGVVTVKTEPLQITTELPGRTSAYRIAEVRPQ
VSGIILKRNFKEGSDIEAGVSLYQIDPATYQATYDSAKGDLAKAQAAANIAQLTVNRYQKLLGTQYISKQEYDQ
ALADAQQANAAVTAAKAAVETARINLAYTKVTSPISGRIGKSNVTEGALVQNGQATALATVQQLDPIYVDVTQ
SSNDFLRLKQELANGTLKQENGKAKVSLITSDGIKFPQDGTLEFSDVTVDQTTGSITLRAIFPNPDHTLLPGMFV
RARLEEGLNPNAILVPQQGVTRTPRGDATVLVVGADDKVETRPIVASQAIGDKWLVTEGLKAGDRVVISGLQK
VRPGVQVKAQEVTADNNQQAASGAQPEQSKSHHHHHH.

dN-AcrA-wt-His6 (His6-tagged AcrA without N-signal peptide):

MTSCDDKQAQQGGQQMPAVGVVTVKTEPLQITTELPGRTSAYRIAEVRPQVSGIILKRNFKEGSDIEAGVSLYQI
DPATYQATYDSAKGDLAKAQAAANIAQLTVNRYQKLLGTQYISKQEYDQALADAQQANAAVTAAKAAVETA
RINLAYTKVTSPISGRIGKSNVTEGALVQNGQATALATVQQLDPIYVDVTQSSNDFLRLKQELANGTLKQENGK
AKVSLITSDGIKFPQDGTLEFSDVTVDQTTGSITLRAIFPNPDHTLLPGMFVRARLEEGLNPNAILVPQQGVTRTP
RGDATVLVVGADDKVETRPIVASQAIGDKWLVTEGLKAGDRVVISGLQKVRPGVQVKAQEVTADNNQQAAS
GAQPEQSKS ENLYFQGHHHHHH

ENLYFQG: TEV recognition site

TolC-wt-His6

MTSKKLLPILIGLSLSGFSSLSQAENLMQVYQQARLSNPELRKSAADRDAAFEKINEARSPLLPQLGLGADYTY
SNGYRDANGINSNATSASLQLTQSIFDMSKWRALTLQEKAAGIQDVTYQTDQQTLILNTATAYFNVLNAIDVLS
YTQAQKEAIYCQLDQTTQRFNVGLVAITDVQNARAQYDTVLANEVTARNNLDNAVEQLRQITGNYYPELAAL
NVENFKTDKPQPVNALLKEAEKRNLSLLQARLSQDLAREQIRQAQDGHLPTLDLTASTGISDTSYSGSKTRGAA
GTQYDDSNMGQNKVGLSFSLPIYQGGMVNSQVKQAQYNFVGASEQLESAHRSVVQTVRSSFNNINASISSINA
YKQAVVSAQSSLDAMEAGYSVGTRTIVDVLDATTTLYNAKQELANARYNYLINQLNIKSALGTLNEQDLLALN
NALSKPVSTNPENVAPQTPEQNAIADGYAPDSPAPVVQQTSARTTTSNGHNPFRNHHHHHHHH

RPL14-wt-His8

MTSIQEQTMLNVADNSGARRVMCIKVLGGSHRRYAGVGDIIKITIKEAIPRGKVKKGDVLKAVVVRTKKGVRR
PDGSVIRFDGNACVLLNNNSEQPIGTRIFGPVTRELRSEKFMKIISLAPEVLHHHHHHHH

FKBP22-wt-His6

MTSTTPTFDTIEAQASYGIGLQVGQQLSESGLQGLLPEALVAGIADALEGKHPAVPVDVVHRALREIHERADAV
RRQRFQAMAAEGVKYLEENAKKEGVNSTESGLQFRVINQGEGAIPARTDRVRVHYTGKLIDGTVFDSSVARGE
PAEFPVNGVIPGWIEALTLMPVGSKWELTIPQELAYGERGAGASIPPFSTLVFEVELLEILHHHHHH

apo-H protein-wt-His8:

MTSSNVPAELKYSKEHEWLRKEADGTYTVGITEHAQELLGDMVFVDLPEVGATVSAGDDCAVAESVKAASDI
YAPVSGEIVAVNDALSDSPELVNSEPYAGGWIFKIKASDESELESLLDATAYEALLEDEHHHHHHHH.

PhoP-wt-His8

MTSRVLVVEDNALLRHHLKVQIQDAGHQVDDAEDAKEADYYLNEHLPDIAIVDLGLPDEDGLSLIRRWRSND
VSLPILVLTARESWQDKVEVLSAGADDYVTKPFHIEEVMARMQALMRRNSGLASQVISLPPFQVDLSRRELSIN
DEVIKLTAFEYTIMETLIRNNGKVVSKDSLMLQLYPDAELRESHTIDVLMGRLRKKIQAQYPQEVITTVRGQGY
LFELRHHHHHHHH

NarL-wt-His8

MTSSNQEPATILLIDDHPMLRTGVKQLISMAPDITVVGEASNGEQGIELAESLDPDLILLDLNMPGMNGLETLDK
LREKSLSGRIVVFSVSNHEEDVVTALKRGADGYLLKDMEPEDLLKALHQAAAGEMVLSEALTPVLAASLRAN
RATTERDVNQLTPRERDILKLIAQGLPNKMIARRLDITESTVKVHVKHMLKKMKLKSRVEAAVWVHQERIFHH
HHHHHH

HflX-wt-His6

MTSFDRYDAGEQAVLVHIYFTQDKDMEDLQEFESLVSSAGVEALQVITGSRKAPHPKYFVGEGKAVEIAEAVK
ATGASVVLFDHALSPAQERNLERLCECRVIDRTGLILDIFAQRARTHEGKLQVELAQLRHLATRLVRGWTHLER
QKGGIGLRGPGETQLETDRRLLRNRIVQIQSRLERVEKQREQGRQSRIKADVPTVSLVGYTNAGKSTLFNRITEA
RVYAADQLFATLDPTLRRIDVADVGETVLADTVGFIRHLPHDLVAAFKATLQETRQATLLLHVIDAADVRVQEN
IEAVNTVLEEIDAHEIPTLLVMNKIDMLEDFEPRIDRDEENKPIRVWLSAQTGAGIPQLFQALTERLSGEVAQHTL
RLPPQEGRLRSRFYQLQAIEKEWMEEDGSVSLQVRMPIVDWRRLCKQEPALIDYLIGGGGGLVPRGSGGGGGH
HHHHHH

LVPRGSG: thrombin recognition site

GrpE-wt-His6

MTSSSKEQKTPEGQAPEEIIMDQHEEIEAVEPEASAEQVDPRDEKIANLEAQLAEAQTRERDGILRVKAEMENL
RRRTELDIEKAHKFALEKFINELLPVIDSLDRALEVADKANPDMSAMVEGIELTLKSMLDVVRKFGVEVIAETN
VPLDPNVHQAIAMVESDDVAPGNVLGIMQKGYTLNGRTIRAAMVTVAKAKAGGGGGLVPRGSGGGGGHHHH
HH

LVPRGSG: thrombin recognition site

TmcA-wt-His6

MTSAELTALHTLTAQMKREGIRRLLVLSGEEGWCFEHTLKLRDALPGDWLWISPRPDAENHCSPSALQTLLGRE
FRHAVFDARHGFDAAAFAALSGTLKAGSWLVLLLPVWEEWENQPDADSLRWSDCPDPIATPHFVQHLKRVLT
ADNEAILWRQNQPFSLAHFTPRTDWYPATGAPQPEQQQLLKQLMTMPPGVAAVTAARGRGKSALAGQLISRIA
GRAIVTAPAKASTDVLAQFAGEKFRFIAPDALLASDEQADWLVVDEAAAIPAPLLHQLVSRFPRTLLTTTVQGY
EGTGRGFLLKFCARFPHLHRFELQQPIRWAQGCPLEKMVSEALVFDDENFTHTPQGNIVISAFEQTLWQSDPETP
LKVYQLLSGAHYRTSPLDLRRMMDAPGQHFLQAAGENEIAGALWLVDEGGLSQQLSQAVWAGFRRPRGNLV
AQSLAAHGNNPLAATLRGRRVSRIAVHPARQREGTGRQLIAGALQYTQDLDYLSVSFGYTGELWRFWQRCGF
VLVRMGNHREASSGCYTAMALLPMSDAGKQLAEREHYRLRRDAQALAQWNGETLPVDPLNDAVLSDDDWL
ELAGFAFAHRPLLTSLGCLLRLLQTSELALPALRGRLQKNASDAQLCTTLKLSGRKMLLVRQREEAAQALFALN
DVRTERLRDRITQWQLFHHHHHHHH

YjaB-wt-His8

MTSMVISIRRSRHEEGEELVAIWCRSVDATHDFLSAEYRTELEDLVRSFLPEAPLWVAVNERDQPVGFMLLSGQ
HMDALFIDPDVRGCGVGRVLVEHALSMAPELTTNVNEQNEQAVGFYKKVGFKVTGRSEVDDLGKPYPLLNLA
YVGARSHHHHHHHH

MTOX-wt-His6

MTSKYDLIIIGSGSVGAAAGYYATRAGLNVLMTDAHMPPHQHGSHHGDTRLIRHAYGEGEKYVPLVLRAQTL
WDELSRHNEEDPIFVRSGVINLGPADSTFLANVAHSAEQWQLNVEKLDAQGIMARWPEIRVPDNYIGLFETDSG
FLRSELAIKTWIQLAKEAGCAQLFNCPVTAIRHDDDGVTIETADGEYQAKKAIVCAGTWVKDLLPELPVQPVR
KVFAWYQADGRYSVKNKFPAFTGELPNGDQYYGFPAENDALKIGKHNGGQVIHSADERVPFAEVASDGSEAFP
FLRNVLPGIGCCLYGAACTYDNSPDEDFIIDTLPGHDNTLLITGLSGHGFKFASVLGEIAADFAQDKKSDFDLTPF
RLSRFQHHHHHH

FolC-wt-His6

MTSIIKRTPQAASPLASWLSYLENLHSKTIDLGLERVSLVAARLGVLKPAPFVFTVAGTNGKGTTCRTLESILMA
AGYKVGVYSSPHLVRYTERVRVQGQELPESAHTASFAEIESARGDISLTYFEYGTLSALWLFKQAQLDVVILEV
GLGGRLDATNIVDADVAVVTSIALDHTDWLGPDRESIGREKAGIFRSEKPAIVGEPEMPSTIADVAQEKGALLQR
RGVEWNYSVTDHDWAFSDAHGTLENLPLPLVPQPNAATALAALRASGLEVSENAIRDGIASAILPGRFQIVSESP
RVIFDVAHNPHAAEYLTGRMKALPKNGRVLAVIGMLHDKDIAGTLAWLKSVVDDWYCAPLEGPRGATAEQLL
EHLGNGKSFDSVAQAWDAAMADAKAEDTVLVCGSFHTVAHVMEVIDARRSGGKHHHHHH

EF-Ts-wt-His6

MTSAEITASLVKELRERTGAGMMDCKKALTEANGDIELAIENMRKSGAIKAAKKAGNVAADGVIKTKIDGNY
GIILEVNCQTDFVAKDAGFQAFADKVLDAAVAGKITDVEVLKAQFEEERVALVAKIGENINIRRVAALEGDVLGS
YQHGARIGVLVAAKGADEELVKHIAMHVAASKPEFIKPEDVSAEVVEKEYQVQLDIAMQSGKPKEIAEKMVEG
RMKKFTGEVSLTGQPFVMEPSKTVGQLLKEHNAEVTGFIRFEVGEGIEKVETDFAAEVAAMSKQSHHHHHH

Rho-wt-His6

MTSNLTELKNTPVSELITLGENMGLENLARMRKQDIIFAILKQHAKSGEDIFGDGVLEILQDGFGFLRSADSSYL
AGPDDIYVSLSQIRRFNLRTGDTISGKIRPPKEGERYFALLKVNEVNFDKPENARNKILFENLTPLHANSRLRME
RGNGSTEDLTARVLDLASPIGRGQRGLIVAPPKAGKTMLLQNIAQSIAYNHPDCVLMVLLIDERPEEVTEMQRL

VKGEVVASTFDEPASRHVQVAEMVIEKAKRLVEHKKDVIILLDSITRLARAYNTVVPASGKVLTGGVDANALH
RPKRFFGAARNVEEGGSLTIIATALIDTGSKMDEVIYEEFKGTGNMELHLSRKIAEKRVFPAIDYNRSGTRKEELL
TTQEELQKMWILRKIIHPMGEIDAMEFLINKLAMTKTNDDFFEMMKRSHHHHHH

Rob-wt-His6

MTSDQAGIIRDLLIWLEGHLDQPLSLDNVAAKAGYSKWHLQRMFKDVTGHAIGAYIRARRLSKSAVALRLTAR
PILDIALQYRFDSQQTFTRAFKKQFAQTPALYRRSPEWSAFGIRPPLRLGEFTMPEHKFVTLEDTPLIGVTQSYSC
SLEQISDFRHEMRYQFWHDFLGNAPTIPPVLYGLNETRPSQDKDDEQEVFYTTALAQDQADGYVLTGHPVMLQ
GGEYVMFTYEGLGTGVQEFILTVYGTCMPMLNLTRRKGQDIERYYPAEDAKAGDRPINLRCELLIPIRRHHHHH
H

GCN5-wt-twin-strep-tag:

MAEPSQAPTPAPAAQPRPLQSPAPAPTPTPAPSPASAPIPTPTPAPAPAPAAAPAGSTGTGGPGVGSGGAGSGGDP
ARPGLSQQQRASQRKAQVRGLPRAKKLEKLGVFSACKANETCKCNGWKNPKPPTAPRMDLQQPAANLSELC
RSCEHPLADHVSHLENVSEDEINRLLGMVVDVENLFMSVHKEEDTDTKQVYFYLFKLLRKCILQMTRPVVEG
SLGSPPFEKPNIEQGVLNFVQYKFSHLAPRERQTMFELSKMFLLCLNYWKLETPAQFRQRSQAEDVATYKVNY
TRWLCYCHVPQSCDSLPRYETTHVFGRSLLRSIFTVTRRQLLEKFRVEKDKLVPEKRTLILTHFPKFLSMLEEEIY
GANSPIWESGFTMPPSEGTQLVPRPASVSAAVVPSTPIFSPSMGGGSNSSLSLDSAGAEPMPGEKRTLPENLTLED
AKRLRVMGDIPMELVNEVMLTITDPAAMLGPETSLLSANAARDETARLEERRGIIEFHVIGNSLTPKANRRVLL
WLVGLQNVFSHQLPRMPKEYIARLVFDPKHKTLALIKDGRVIGGICFRMFPTQGFTEIVFCAVTSNEQVKGYGT
HLMNHLKEYHIKHNILYFLTYADEYAIGYFKKQGFSKDIKVPKSRYLGYIKDYEGATLMECELNPRIPYTELSHII
KKQKEIIKKLIERKQAQIRKVYPGLSCFKEGVRQIPVESVPGIRETGWKPLGKEKGKELKDPDQLYTTLKNLLA
QIKSHPSAWPFMEPVKKSEAPDYYEVIRFPIDLKTMTERLRSRYYVTRKLFVADLQRVIANCREYNPPDSEYCR
CASALEKFFYFKLKEGGLIDKGSSAWSHPQFEKGGGSGGGSGGSAWSHPQFEK

Tip60-wt-twin-strep-tag:

MAEVGEIIEGCRLPVLRRNQDNEDEWPLAEILSVKDISGRKLFYVHYIDFNKRLDEWVTHERLDLKKIQFPKKE
AKTPTKNGLPGSRPGSPEREVPASAQASGKTLPIPVQITLRFNLPKEREAIPGGEPDQPLSSSSCLQPNHRSTKRK
VEVVSPATPVPSETAPASVFPQNGAARRAVAAQPGRKRKSNCLGTDEDSQDSSDGIPSAPRMTGSLVSDRSHDDI
VTRMKNIECIELGRHRLKPWYFSPYPQELTTLPVLYLCEFCLKYGRSLKCLQRHLTKCDLRHPPGNEIYRKGTIS
FFEIDGRKNKSYSQNLCLLAKCFLDHKTLYYDTDPFLFYVMTEYDCKGFHIVGYFSKEKESTEDYNVACILTLP
PYQRRGYGKLLIEFSYELSKVEGKTGTPEKPLSDLGLLSYRSYWSQTILEILMGLKSESGERPQITINEISEITSIK
KEDVISTLQYLNLINYYKGQYILTLSEDIVDGHERAMLKRLLRIDSKCLHFTPKDWSKRGKWGSSAWSHPQFE
KGGGSGGGSGGSAWSHPQFEK

1.2 Medium and buffer solutions

2YT medium (16 g/L tryptone, 10 g/L yeast extract, 5 g/L NaCl, pH 7.4) was used for bacterial culture and protein expression, SOC medium (20 g/L tryptone, 5 g/L yeast extract, 0.5 g/L NaCl, 2.5 mM KCl, 10 mM $MgCl_2$, 20 mM glucose) was used for heat stroke recovery of the complement *E. coli* cells. Lysis buffer (50 mM NaH_2PO_4, 300 mM NaCl, 10 mM imidazole, 10%v/v glycerol, 1%v/v Tween-20, 0.5 mg/mL lysozyme, 1 mM PMSF, pH 7.5), washing buffer (50 mM NaH_2PO_4, 300 mM NaCl, 20 mM imidazole, pH 7.5), elution buffer (50 mM NaH_2PO_4, 300 mM NaCl, 250 mM imidazole, pH 7.5), dialysis buffer (20 mM NaH_2PO_4, 150 mM NaCl, 10% glycerol, 0.5 mM TCEP, pH 7.5 or

8) were used for bacteria lysis, protein purification and storage.

1.3 Site-specific incorporation of o-NBAK (or other photoUaas) into proteins, expression and purification

The plasmid pBK-*Mm*PylRS-Y384F/Y306A encoding the evolved PylRS and its cognitive tRNA which inserts o-NBAK or TmdZLys into the in-frame amber codon site on target genes in *E. coli* cells was described in the previous literature.[12,15] The plasmid pBK-AbKRS encoding the evolved PylRS and its cognitive tRNA which inserts AbK into the in-frame amber codon site on target genes in *E. coli* cells was described in the previous literature.[13] The plasmid pBK-mPyTKRS encoding the evolved PylRS and its cognitive tRNA which inserts mPyTK into the in-frame amber codon site on target genes in *E. coli* cells was described in the previous literature.[14] Plasmids pTAK-PatZ-I800TAG, pTAK-PatZ-F810TAG, pTAK-PatZ-L813TAG, pTAK-PatZ-S816TAG, pTAK-YiaC-F75TAG, pTAK-LplA-A138TAG, pTAK-TmcA-R457TAG, or pTAK-YjaB-F77TAG were co-transformed with pBK-*Mm*PylRS-384F/306A into BL21(DE3) complement cells (50 μL) using heat shock. The transformants were recovered in 500 μL SOC media at 37 ℃ for 1 hour before plating to 2YT agar plate containing 50 μg/mL of kanamycin and 34 μg/mL of chloramphenicol. A single colony from the plate was picked and used to inoculate 5 mL 2YT containing 50 μg/mL kanamycin and 34 μg/mL chloramphenicol. A 2-mL aliquot of overnight culture was then used to inoculate 200 mL 2YT containing the same concentrations of antibiotics. The cells were grown at 37 ℃ until OD600 reached ~0.5. The culture was divided into two 100 mL portions. One portion of the culture was supplemented with 1 mM o-NBAK (or 1mM TmdZLys, for expression of PatZ-L813TmdZLys) and the other portion served as a control by adding medium at the same volume. After culturing for another 20 min at 37 ℃, the protein expression was induced by adding 0.5 mM IPTG at 30 ℃ for 8 hours. The cells were pelletized in 50-mL conical tubes by centrifugation at 4200 g for 30 min at 4 ℃ and stored at -80 ℃. For expression of PatZ-L813Abk or PatZ-L813mPyTK, the produce is basically the same, except that 1) plasmids pTAK-PatZ-L813TAG were co-transformed with pBK-AbKRS or pBK-mPyTKRS into BL21(DE3) complement cells (50 μL) using heat shock, and 2) cell culture was supplemented with 1 mM Abk or mPyTK after OD600 reached ~0.5.

Next day, the cell pellets were re-suspended in 10 mL lysis buffer. After incubation on ice for 30 min, the lysates were further treated with ultrasonication. Following centrifuge, the supernatants were incubation with 100 μL Ni-NTA agarose beads at 4 ℃ for 2 hours with gentle shaking. The resin was centrifuged briefly and washed 10 column volume (CV) of lysis buffer (devoid of lysozyme) and 10 CV of washing buffer. Finally, the protein was eluted with elution buffer and the buffer was exchanged against dialysis buffer. For GrpE and HflX, the proteins were further treated by thrombin (enzyme : protein = 1 : 1000) at 4 ℃ overnight and incubated with Ni-NTA resin to remove His6-tag peptides. The flow-through was collected. A 10-μL aliquot from the elution fraction was mixed with one fourth amount of 5X Laemmli buffer and heated at 98 ℃ for 8 minutes, before analyzed by SDS-PAGE. The rest protein was aliquoted, flash-frozen by liquid nitrogen and stored at -80 ℃.

1.4 Expression and purification of dN-AcrA-wt-His6 and site-specifically acetylated AcrA (K131Kac)

dN-AcrA-wt-His6 was expressed as previous study reported.[17] Acetylated AcrA (dN-AcrA-K131Kac) was produced according to previous study reported.[6] Briefly, *E. coli* strain BL21(DE3) was transformed with plasmid pBK-AcKRS3 and pTAK-dN-AcrA-K131(TAG) and then grown overnight. One liter of 2YT medium was inoculated with 20 mL bacteria seed and incubated at 37℃. When OD600 reached 0.5, the culture was supplemented with one liter

of fresh 2YT with 10 mM acetyllysine (AcK). After culturing for another 20 min at 37 ℃, protein expression was induced by addition of 0.5 mM IPTG for 8 h at 30℃, cells were harvested by centrifugation, and stored at -80℃. Protein purification was performed using the same procedures as mentioned in **Supplemental Experimental Procedures 1.3**.

1.5 Expression and purification of wild type proteins

BL21(DE3) cells were respectively transformed with each expression plasmid using heat shock and recovered in 500 μL SOC media and incubated at 37 ℃ for 1 hour before plating to 2YT agar plate containing 34 μg/mL of chloramphenicol. A single colony from the plate was picked and used to inoculate 50-mL 2YT containing corresponding probiotic. A 10-mL aliquot of overnight culture was then used to inoculate 1-L 2YT containing the same concentrations of antibiotics. The cells were grown at 37 ℃ until OD600 reached ~0.5, and the protein expression was induced by adding 0.5 mM IPTG. Protein purification and analyses were performed using the same procedure as mentioned in **Supplemental Experimental Procedures 1.3**.

1.6 Expression and purification of membrane protein TolC

Purification and expression procedure of TolC was similar to previous studies with modifications. [17,18] TolC–6His were extracted from the membrane fraction of BL21(DE3). Cell pellets from 250 mL cultures were thawed on ice and resuspended in 10 mL of 50 mM PBS (pH7.5), containing 300 mM NaCl, 1 mM PMSF, 10%v/v glycerol and 1 mg/ml lysozyme (buffer A). After sonication, cell fragments were removed by centrifugation (8000 g for 30 min at 4 ℃) and the whole membrane fraction was recovered by ultracentrifugation at 110 000 g for 60 min at 4 ℃. Membranes were resuspended in 10 mL buffer A containing 0.05% DDM and stirred on ice for at least 60 min before centrifugation (8 000 g, 20 min). His-tagged TolC protein was purified using the same procedure as mentioned in **Supplemental Experimental Procedures 1.3**, and the eluted protein was dialyzed in 20 mM PBS buffer containing 150 mM NaCl, 5% glycerol and 0.03% DDM.

1.7 Expression of Tip60-369o-NBAK or GCN5-575o-NBAK in 293-F cells

FreeStyle™ 293-F cells were cultured in SMM 293-TII Expression Medium and shaken at 135 rpm in incubator at 37 ℃ with 8% CO_2. Cells were passaged every 2–3 days, starting with the cell density around 0.2×10^6 cells per mL. Transfection was performed at cell density around 2.0×10^6 cells per mL. Polyethyleneimine molecular mass 40,000 (PEI, 1 mg/mL) of 60 μL was diluted in 1 mL SMM 293-TII Expression Medium. DNA mixture (Tip60-C369TAG or GCN5-E575TAG : PylRS = 1 : 3; or empty vector: PylRS = 1 : 3) of 20 μg was diluted in 1 mL SMM 293-TII Expression Medium. Diluted DNA and PEI solution were mixed and incubated at room temperature for 15 min before adding to the cell culture. o-NBAK (0.5 mM) was added immediately after transfection for o-NBAK incorporation. Forty to forty-eight hours after transfection, the cells were collected for subsequent analysis.

2. *In vitro* photo-crosslinking reactions

Purified o-NBAK-containing proteins (in PBS buffer 150 mM NaCl, pH 7.5) was mixed with partner proteins (in PBS buffer 150 mM NaCl, pH 7.5), the mixture was divided and transferred to a 96-well plate (BD Biosciences) and irradiated at 10 mm distance with a handheld 365-nm UV lamp for 15 minutes on ice. Samples were collected, diluted with Laemmli buffer and analyzed by SDS-PAGE.

3. Photo-crosslinking of o-NBAK (or other photoUaas) incorporated acyltransferases in living cells

3.1 Photo-crosslinking in *E. coli* cells

After the enzyme of interested is expressed for 5 hours (alternatively, 1 hour and 3 hours in the time-resolved profiling of PatZ substrate experiment), cells were harvested, washed twice with PBS (Gibco) and re-suspended with PBS (with the OD600 of 90-fold diluted sample equal to 0.4~0.5). The mixture was divided into two portions. One portion was transferred to 6-well plate (3-4 mL/well) and irradiated at 10 mm distance with a hand held UV lamp for 20 min on ice (365-nm for o-NBAK, AbK; 302-nm for mPyTK). The other portion was kept in dark as the control group. The cells were harvested by centrifugation at 1,000 g for 5 min and lysed in lysis buffer. The lysate was incubated with Ni-NTA agarose beads at 4 ℃ for 2 hours with gentle shaking. Protein purification and analyses were performed using the same procedure as mentioned in **Supplemental Experimental Procedures 1.3**. Samples were resolved with SDS-PAGE. For substrate profiling experiment, the whole gel lane above the free enzyme was excised for in-gel digestion. For crosslinking site identification, the gel lane above the free enzyme were sliced into several gel bands for in-gel digestion.

3.2 Photo-crosslinking in human 293-F cells

The cells were collected, washed with cold PBS twice and illuminated for 15 min at 10 mm distance from a ZF-7A 16 W 365 nm UV light on ice. The cells were collected, washed with cold PBS, lysed ultrasonically in cooled RIPA lysis buffer supplemented with containing protease inhibitor cocktail. The lysates were centrifuged at 15000 *g* for 30 min at 4 ℃. The supernatant was obtained and normalized by BCA protein assay. The lysates were subjected to western blot or affinity enrichment by MagStrep type3 XT beads (IBA, #2-4090-002). For affinity enrichment, 0.1 mL MagStrep type3 XT beads were added to the solution and incubated at 4 ℃ overnight. The beads were washed with 2-fold diluted RIPA lysis buffer and wash buffer (100 mM Tris•HCl, 150 mM NaCl, 1 mM EDTA, pH = 8) three times each. Proteins attached to the beads were eluted in 2× Laemmli buffer by heating at 95 ℃ for 8 min. The eluates were resolved by SDS-PAGE and visualized by Silver Stain Kit (Thermo Fisher, #24612). Gel lanes above the free Tip60 or GCN5 enzyme were cut for subsequent in-gel digestion.

4. *In vitro* enzymatic activity assays

The *in vitro* enzymatic acylation was performed in the reaction buffer containing 20 mM sodium phosphate (pH 8.0), 150 mM NaCl, 10% glycerol, 0.5 mM tris(2-chloroethyl) phosphate (TCEP). The *in vitro* acetylation by PatZ was carried out by incubation of PatZ (20 μg), acetyl-CoA (with concentrated indicated in Fig. 4 for AcrA; or 0.1 mM for DnaJ, HflX), and candidate substrates (30 μg) in 100 μL reaction buffer at 37 °C for two hours. The *in vitro* acetylation by YiaC was performed in 50 μL reaction buffer containing YiaC (4 μg), acyl-CoA (1 mM for acetyl CoA; 0.5 mM for 2-hydroxyisobutyryl-CoA, propionyl-CoA, or malonyl-CoA), and candidate substrates (20 μg) at 37 °C for three hours. The *in vitro* acetylation by TmcA was performed in 50 μL reaction buffer containing TmcA (15 μg), 2-hydroxyisobutyryl-CoA (0.5 mM), ATP (1 mM) and candidate substrates (20 μg) at 37 °C for three hours. The *in vitro* acetylation by YjaB was performed in 50 μL reaction buffer containing YjaB (4 μg), Ac-CoA (0.5 mM), and candidate substrates (20 μg) at 37 °C for three hours. The samples were resolved by SDS-PAGE for subsequent Western blot

analysis, or in-gel digestion and LC-MS/MS analysis.

The *in vitro* lipoylation reaction was performed in the buffer containing 40 mM PBS (pH 7.0), 0.5 mM dithiothreitol (DTT), 10 μM LplA, 20 μM PhoP or NarL, 2 mM ATP, 2 mM $MgCl_2$, 1 mM lipoic acid for 3 h at 37 ℃. Then, the reaction mixture was separated by SDS-PAGE, and subjected to in-gel digestion and LC-MS/MS analysis.

5. Western blot analysis

Laemmli buffer with additional *β*-ME (2%) to denature the protein sample, protein samples in SDS-PAGE sample buffer were boiled for 8 min, resolved on a SDS-PAGE gel and transferred to PVDF membrane (Thermo Scientific). The membrane was blocked in 5% skim milk in TBST (50 mM Tris, 150 mM NaCl, 0.05% Tween-20, pH 7.5) at room temperature for 1 hour. Then the membrane was incubated with mouse anti-His tag antibody (1:10000, Proteintech, Cat. No. HRP-66005), mouse anti-vinculin monoclonal antibody (1:2000, Cat. No. 66305-1-Ig), mouse monoclonal anti-acetyl lysine antibody (1:1000, Cell Signaling Technology, Cat. No. 9681), mouse anti-strep-tag II monoclonal antibody (1:2000, Abbkine, Cat. No. ABT2230), rabbit polyclonal FtsZ antibody (1:1000, CUSABIO, Cat. No. CSB-PA359270HA01EGX) in TBST at 4 ℃ overnight. The membrane was washed with TBST (4 × 5 min) before addition of the secondary goat anti-mouse horseradish peroxidase conjugate (1:4000, Proteintech, Cat. No. SA00001-1) or HRP-conjugated goat anti-rabbit antibody (1:5000, Cell Signaling Technology, Cat. No. 7074). After 1 hour, the membrane was washed with TBST (4 × 5 min). Final detection of HRP activity was performed using ECL Plus chemiluminescent substrate (Thermo Fisher Scientific). Image was acquired with Image Quant LAS 4000 or ChemiScope 6300. Uncropped images can be found in the Source Data File.

6. Biolayer interferometry (BLI) assay

The binding characteristics between dN-AcrA-wt or dN-AcrA-K131Kac and TolC were determined using anti-HIS (HIS2) biosensors in the Octet RED96 system (ForteBio Inc., Menlo Park, CA, USA). First, the membrane protein TolC was loaded onto the HIS2 biosensors at 4 μM in PBS containing 150 mM NaCl, 0.03% DDM and 5% glycerol. The biosensors were blocked with His8 tagged MBP protein (2 mg/ml) for 60 s. Diluted AcrA in PBS solution containing 150 mM NaCl, 0.03% DDM and 5% glycerol was then added onto the HIS2 biosensors loaded with TolC. The real time binding response (Δλ in nanometer, nm) between AcrA and TolC was calculated by subtracting the nonspecific binding of AcrA to the HIS2 biosensors from the binding of AcrA with TolC.

7. Profiling of protein-protein interactome (PPI) by BS2G-mediated crosslinking

E. coli BL21(DE3) cells were inoculated into 2YT media and grow at 37 ℃ overnight. The overnight cultures were inoculated into fresh 2YT media, grown till OD600 of 0.8. Cells were pelletized by centrifugation at 4200 *g* for 30 min at 4 ℃ and stored at -80 ℃. Cell pellets were re-suspended in denaturing lysis buffer (8M urea, 20mM sodium phosphate, 150 mM NaCl, pH = 7.5) supplemented with protease inhibitor cocktail. After incubation on ice for 30 min, the lysate was further treated with ultrasonication and the debris were removed by centrifugation (15000 *g*). Protein concertation in the clarified lysate was quantified by BCA assay. 0.5 mL lysate (8 mg/mL) was incubated with purified

His6-tagged PatZ-wt or PatZ-L813o-NBAK protein (1 mg/mL) for 30 min on ice. The crosslinking was triggered by addition of BS2G crosslinker (1 mM in DMSO) as experimental group and DMSO of the same volume as control group. The reaction mixture was gently shaken at room temperature for 1h and quenched by 1 M Tris-HCl buffer (pH 7.5). Then, the PatZ-wt (or PatZ-L813o-NBAK) and crosslinked complex were purified by Ni-NTA affinity purification as described in **Supplemental Experimental Procedures 1.3.** The purified proteins were sequentially buffer-exchanged into denaturing buffer (8M urea, 50 mM NH_4HCO_3, pH 8.0) and digestion buffer (2M urea, 50 mM NH_4HCO_3, pH 8.0). To 50 μg protein samples (0.5 mg/mL), 1 μg trypsin was added, and incubated at 37 °C overnight. The digested peptides were vacuum-dried and desalted with ZipTip C18 pipette tips (Merck-Millipore, Darmstadt, Germany) which is ready for subsequent LC-MS/MS analysis.

8. In-gel digestion

The gel slices containing bands of interest were destained with 50% ethanol. After balanced with distilled water, the bands were cut into cubes (around 1mm^3) and dehydrated in acetonitrile (ACN). The cubes were then incubated with 25 mM NH_4HCO_3 containing 10 mM DTT at 56 ℃ for 1 h and then with 25 mM NH_4HCO_3 containing 55 mM iodoacetamide (IAA) at r.t. for 45 min in darkness. The gel cubes were then dehydrated in 50% ACN, vacuum-dried, and incubated with 50 mM NH_4HCO_3 containing 10-20 ng trypsin (sequencing grade, Hualishi Scientific, Beijing) at 37 ℃ overnight. For samples of PatZ-L813o-NBAK mediated *in vitro* and in-cell crosslinking, *in vitro* crosslinking between LplA-A138o-NBAK and substrate (apoH or PhoP), and lipoylation of NarL by wild type LplA, trypsin-digested samples were further incubated with 10-20 ng endoproteinase Glu-C (sequencing grade, Roche) at 37 ℃ for additional 4 h. The peptides were sequentially extracted with 50% ACN (containing 5% TFA), 75% ACN (containing 0.1% TFA) and 100% ACN, and vacuum-dried. The peptides were desalted with ZipTip C18 pipette tips (Merck-Millipore, Darmstadt, Germany) and ready for subsequent LC-MS/MS analysis.

9. LC-MS/MS analysis

The peptides were reconstituted in buffer A (0.1% formic acid, 2% ACN) and loaded onto 15 cm C18 reversed phase capillary analytical column (3 μm particle size, 90 Å pore size, Dikma Technologies, Lake Forest, CA) connected to an EASY-nLC 1000 HPLC system (Thermo Fisher Scientific, Waltham, MA).

For analyses of the samples from substrate profiling by PatZ-L813o-NBAK (or PatZ-L813AbK, PatZ-L813mPyTK) crosslinking in living cells, the peptides were eluted with a linear gradient of buffer B (0.1% formic acid, 90% ACN) from 8% to 32% in 58 min, followed by an increase to 48% in 6 min and further increase to 80% in 2 min at a constant flow rate of 300 nL/min. The samples from crosslinking substrate profiling in living cells (by YiaC-F75o-NBAK, LplA-A138o-NBAK, TmcA-R457o-NBAK, YjaB-F77o-NBAK, GCN5-E575o-NBAK or Tip60-C369o-NBAK), substrate validation by *in vitro* enzymatic assays (catalyzed by wild type YiaC, LplA, TmcA, and YjaB), as well as PPI profiling by BS2G-mediated crosslinking, are analyzed as follows: the peptides were eluted with a linear gradient of buffer B from 8% to 13% in 20 min, followed by an increase to 26% in 31 min, to 45% in 5min and to 80% in 1 min at a constant flow rate of 300 nL/min. The eluted peptides were ionized and sprayed into an Orbitrap Fusion mass spectrometer (Thermo Fisher Scientific, Waltham, MA) in a positive mode. The mass spectrometric

analysis was carried out in a "Top speed" data dependent acquisition (DDA) mode with a cycle time of 3 s. The peptides with a range of m/z 350-1300 were analyzed by an Orbitrap mass analyzer with a resolution of 120000, automatic gain control (AGC) target of 5×10^5 and maximum ion injection time (IT) of 50 ms. The dynamic exclusion was set as 60 s, and the charge inclusion was set to 2~6+. The isolated precursor ions were subjected to fragmentation via Higher-energy collisional dissociation (HCD) with a normalized collision energy (NCE) of 32%, and analyzed by ion trap analyzer.

For analyses of samples from *in vitro* acetylation by wt PatZ, the peptides were eluted with a linear gradient of buffer B from 7% to 35% in 24 min, followed by an increase to 80% in 3 min. The eluted peptides were ionized and sprayed into an Orbitrap Fusion mass spectrometer in a positive mode. The mass spectrometric analysis was carried out in a "Top 20" DDA mode. The peptides with a range of m/z 350-1500 were analyzed by an Orbitrap mass analyzer with a resolution of 240000, AGC target of 7×10^5 and IT of 50 ms. The isolated precursor ions were subjected to fragmentation via collision-induced dissociation (CID) with a NCE of 35%, and analyzed by ion trap analyzer.

To identify crosslinking sites, the peptides were eluted with a linear gradient of buffer B from 5% to 7% in 13 min, followed by an increase to 10% in 20 min, to 25% in 55 min, 45% in 22 min, 80% in 3 min at a constant flow rate of 300 nL/min. The eluted peptides were ionized and sprayed into an Orbitrap Fusion mass spectrometer in a positive mode. The mass spectrometric analysis was carried out in a "Top speed" DDA mode with a cycle time of 3 s. The peptides with a range of m/z 300-1700 were analyzed by an Orbitrap mass analyzer with a resolution of 120000, AGC target of 5×10^5 and maximum IT of 100 ms. The charge inclusion was set to 3~7+. The isolated precursor ions were subjected to fragmentation via HCD with a NCE of 27-33% (stepped collision energy = 3%), and analyzed by Orbitrap analyzer at a resolution of 15000, automatic AGC target of 2×10^5, IT of 250 ms, the dynamic exclusion was set as 10s.

10. MS data analysis

For identification of proteins in experiments of substrate profiling by enzyme-o-NBAK (or PatZ-L813AbK, PatZ-L813mPyTK) mutants or protein-protein interactome profiling by BS2G-mediated crosslinking, raw MS data were processed with MaxQuant software[19] (v. 1.6.6.0) against UniProt *Escherichia coli* database (2019.2 release, 4350 sequences) or human database (2019.2 release, 95943 sequences). Trypsin was set as digestion enzyme, allowing up to 2 missed cleavage. Cysteine carbamidomethylation (57.02 Da) was set to fixed modification, and protein N-terminal acetylation (28.03 Da) and methionine oxidation (15.99 Da) as variable modifications. The second peptide feature was disabled. For protein identification, at least two peptides were required. PSM and protein identifications were filtered to 1% false discovery rate (FDR). Other parameters were set as default. The "proteingroups.txt" file generated by MaxQuant were further processed to screen for enriched proteins. The proteins identified as "reverse", "potent contaminant" and "only identified by site" were filtered. Then, the intensities of proteins in each sample were log2-transformed and normalized by subtracting the median $\log_2$(intensity) in that sample (so that the median $\log_2$(intensity) = 0). Data from UV-treated (+UV) replicates and untreated (-UV) replicates were grouped, separately. Proteins were further filtered to have valid values across all replicates in at least one group. The missing values were replaced with the minimal intensity of the data matrix. Proteins with +UV/-UV ratio > 2 and p-value < 0.05 (two-sided Student's t-test) were considered as enriched.

For data from *in vitro* acetylation assays, MS raw data were analyzed by Mascot search engine (v2.3, Matrix Science, London, UK)[20] against the corresponding protein sequences. Mass tolerances were set to 10 ppm for precursor ions, 0.5 Da for fragment ions in ion trap detection. Trypsin was set as digestion enzyme. Up to 2 missed cleavage were allowed. Cysteine carbamidomethylation (57.02 Da) was set to fixed modification, and protein N-terminal acetylation (28.03 Da), lysine acetylation (28.03 Da), and methionine oxidation (15.99 Da) as variable modifications. For data from other *in vitro* acylation assay, the variable modification of lysine acetylation (28.03 Da) was replaced by lysine 2-hydroxyisobutyrylation (86.04 Da), propionylation (56.03 Da), or malonylation (86.00 Da, with neutral loss of 44 Da, -CO_2). For identification of lipoylation on PhoP and NarL, the parameters are basically the same, except: 1) a mass corresponding to thiol-reduced and IAA-alkylated lipoyl group (C12H20N2O3S2, 304.09 Da) instead of acetylation was set as variable modification of lysine; 2) for identification of lipoylation on NarL, trypsin-GluC (cleaving at C-terminal of K/R/E/D) was set as digestion enzyme instead of trypsin. Identified peptides were filtered with a Mascot ion score cutoff of 20 and the quality of peptide-spectrum matches were manually checked. For quantification of acylation or lipoylation level, peak areas of precursor ions of acylated or lipoylated peptides were manually integrated. The ratio of peak areas of modified peptides from different experimental conditions were calculated, and then normalized against the averaged ratio of the peak areas of 3-4 unmodified peptides.

All LC-MS/MS data were collected with XCalibur (3.0). For identification of *in vitro* crosslinking sites, raw data were searched against MeroX search engine[21] (v2.0) against the sequences for proteins of interest. In fasta files, the amino acid residues mutated into o-NBAK in the sequence of enzyme were replaced with letter U. Correspondingly, letter U was defined to represent o-NBAK in MeroX with a monoisotopic mass of 337.1273 Da (C15H19N3O6). C-terminal of R/K were set as protease site for trypsin and C-terminal of D/E was also set as protease site if Glu-C was used in protein digestion. Three missed cleavages were allowed for each amino acids and total missed cleavage were set to 3. Cysteine carbamidomethylation was set to fixed modification and methionine oxidation as variable modifications. The composition of crosslinker is set as –H4O2 (–36.0211 Da, loss of two water molecules after crosslinking. Please refer to Fig. 2b in main text for the crosslinking mechanism). N-terminal amine and lysine were set as potential crosslinking sites. For mass comparison, a signal-to-noise ratio of ≥ 1.5 was applied and mass deviations of 5 ppm and 10 ppm were applied for precursor and product ions, respectively. The quality of the crosslink-spectrum matches was manually inspected. For identification of the crosslinking sites in living cells, the MS-cleavage feature was considered during database-searching using MeroX software. Totally, three different MS-cleavage features were considered including the MS-cleavage of 1) carbamate C-N bond (α peptide + C9H3NO3, β peptide – C9H3NO3), 2) benzyl C-O bond (α peptide + C8H3NO, β peptide – C8H3NO), 3) carbamate C-O bond (α peptide + C8H5NO, β peptide – C8H5NO) of the crosslinked peptides during MS/MS fragmentation. All MS/MS spectra of crosslinking peptide identified by database search were manually checked and only the high quality spectra were reported.

The MS/MS spectra of identified acylated or crosslinked peptides were annotated by Mascot or MeroX software, respectively. In some cases, the annotated MS/MS spectra were displayed using pLabel[22,23] for better visualization.

11. Bioinformatics.

The gene ontology (GO) enrichment analysis and functional classification of candidate substrates were performed with PANTHER database[24] (v16.0). GO terms with BH-adjusted P-value (false discovery rate, FDR) < 0.05 were

considered as significantly enriched.

12. Homology modeling and protein-protein docking.

Homology models for YiaC and the GNAT domain of PatZ were constructed using the Mod Web server (https://modbase.compbio.ucsf.edu/modweb/) with the slow restraint selected for model generation. The templates used for each of the final homology models were PDB ID 2kcw for YiaC and PDB ID 4nxy for the GNAT domain of PatZ. The binding model of LplA-PhoP is generated by GRAMM-X Protein-Protein Docking Web Server (v.1.2.0)[3] with default parameters using crystal structures from PDB database LplA (PDB ID: 3A7A), PhoP (PDB ID: 2PKX) and NarL (PDB ID: 1RNL). Protein structures are visualized with Discover Studio 3.5 client.

13. Drug sensitivity assay.

pET24a-*acrA-wt*, pET24a-*acrA-K131A* or pET24a-*acrA-K131Q* plasmids was transformed into *E. coli* BW2513*ΔacrA* competent cell. Positive colonies harboring the target plasmids were selected and grown in LB medium supplemented with Kanamycin at 50 μg/mL overnight. 0.1 mL cultures were inoculated into 20 mL LB medium supplemented with Kanamycin at 50 μg/mL and antibiotics (Erythromycin at 25 μg/ml, Oxacillin at 100 μg/mL, Linezolid at 65 μg/mL). Growth curve was measured at OD600 every 1h.

References

1. de Diego Puente, T. et al. The protein acetyltransferase PatZ from Escherichia coli is regulated by autoacetylation-induced oligomerization. *J. Biol. Chem.* **290**, 23077-23093 (2015).

2. Fujiwara, K. et al. Global conformational change associated with the two-step reaction catalyzed by Escherichia coli lipoate-protein ligase A. *J. Biol. Chem.* **285**, 9971-9980 (2010).

3. Tovchigrechko, A. & Vakser, I.A. Gramm-X public web server for protein–protein docking. *Nucleic Acids Res.* **34**, W310-W314 (2006).

4. Bachhawat, P. & Stock Ann, M. Crystal structures of the receiver domain of the response regulator phop from Escherichia coli in the absence and presence of the phosphoryl analog beryllofluoride. *J. Bacteriol.* **189**, 5987-5995 (2007).

5. Ren, J. et al. Metabolic intermediate acetyl phosphate modulates bacterial virulence via acetylation. *Emerg. Microbes Infect.* **8**, 55-69 (2019).

6. Ren, J. et al. Acetylation of lysine 201 inhibits the DNA-binding ability of phop to regulate salmonella virulence. *PLOS Pathog.* **12**, e1005458 (2016).

7. Xie, L., Zeng, J., Luo, H., Pan, W. & Xie, J. The roles of bacterial Gcn5-related N-acetyltransferases. *Crit. Rev. Eukaryot. Gene Expr.* **24**, 77-87 (2014).

8. Vetting, M.W. et al. Structure and functions of the GNAT superfamily of acetyltransferases. *Arch. Biochem. Biophys.* **433**, 212-226 (2005).

9. Liu, X. et al. The structural basis of protein acetylation by the p300/CBP transcriptional coactivator. *Nature* **451**, 846-850 (2008).

10. Marmorstein, R. & Roth, S.Y. Histone acetyltransferases: Function, structure, and catalysis. *Curr. Opin. Genet. Dev.* **11**, 155-161 (2001).

11. Huang, H. et al. Landscape of the regulatory elements for lysine 2-hydroxyisobutyrylation pathway. *Cell Res.* **28**, 111-125 (2018).

12. Hu, W. et al. Genetically encoded residue-selective photo-crosslinker to capture protein-protein interactions in living cells. *Chem* **5**, 2955-2968 (2019).

13. Ai, H.-w., Shen, W., Sagi, A., Chen, P.R. & Schultz, P.G. Probing protein–protein interactions with a genetically encoded photo-crosslinking amino acid. *ChemBioChem* **12**, 1854-1857 (2011).

14. Tian, Y. et al. Genetically encoded 2-aryl-5-carboxytetrazoles for site-selective protein photo-cross-linking. *J. Am. Chem. Soc.* **139**, 6078-6081 (2017).

15. Yanagisawa, T. et al. Wide-range protein photo-crosslinking achieved by a genetically encoded N^{ε}-(benzyloxycarbonyl)lysine derivative with a diazirinyl moiety. *Mol. Biosyst.* **8**, 1131-1135 (2012).

16. Seo, J.-H., Baek, S.-W., Lee, J. & Park, J.-B. Engineering Escherichia coli BL21 genome to improve the heptanoic acid tolerance by using CRISPR-Cas9 system. *Biotechnol and Bioproc E* **22**, 231-238 (2017).

17. Masi, M., Pagès, J.-M. & Pradel, E. Overexpression and purification of the three components of the enterobacter aerogenes AcrA–AcrB–TolC multidrug efflux pump. *J. Chromatogr. B* **786**, 197-205 (2003).

18. Morona, R., Manning, P.A. & Reeves, P. Identification and characterization of the TolC protein, an outer membrane protein from escherichia coli. *J. Bacteriol.* **153**, 693-699 (1983).

19. Tyanova, S., Temu, T. & Cox, J. The maxquant computational platform for mass spectrometry-based shotgun proteomics. *Nat. Protoc.* **11**, 2301-2319 (2016).

20. Perkins, D.N., Pappin, D.J.C., Creasy, D.M. & Cottrell, J.S. Probability-based protein identification by searching sequence databases using mass spectrometry data. *Electrophoresis* **20**, 3551-3567 (1999).

21. Götze, M. et al. Automated assignment of MS/MS cleavable cross-links in protein 3D-structure analysis. *J. Am. Soc. Mass Spectrom.* **26**, 83-97 (2015).

22. Li, D. et al. Pfind: A novel database-searching software system for automated peptide and protein identification via tandem mass spectrometry. *Bioinformatics* **21**, 3049-3050 (2005).

23. Wang, L.-h. et al. Pfind 2.0: A software package for peptide and protein identification via tandem mass spectrometry. *Rapid Commun. Mass Spectrom.* **21**, 2985-2991 (2007).

24. Mi, H. et al. Panther version 16: A revised family classification, tree-based classification tool, enhancer regions and extensive api. *Nucleic Acids Res.* **49**, D394-D403 (2020).

Uncropped scans of blots and gels in Supplementary Figures

Supplementary Fig. 2

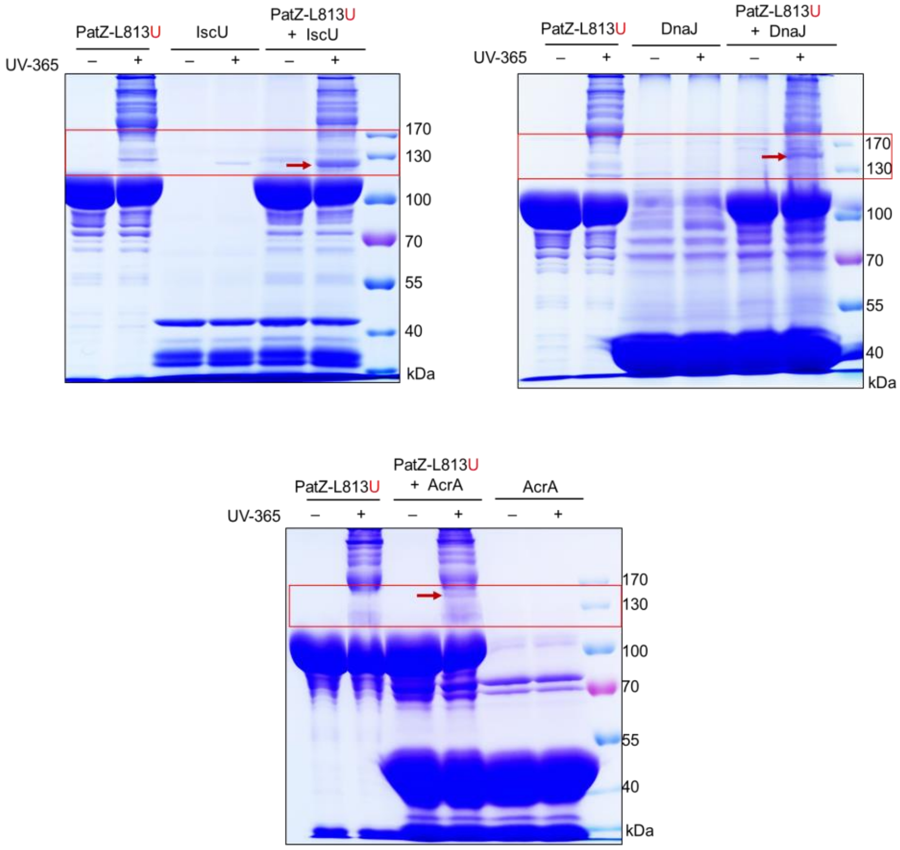

Supplementary Fig. 4

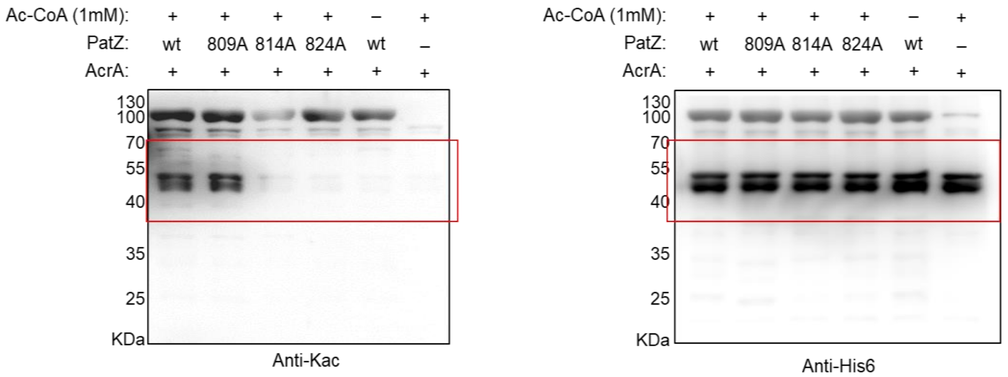

Supplementary Fig. 6b

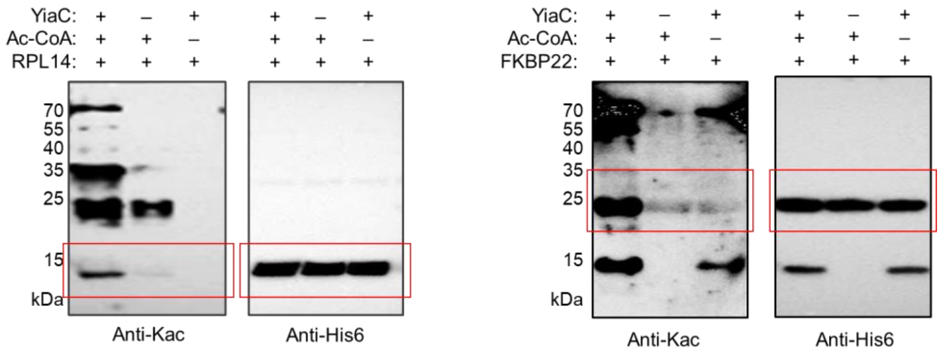

Supplementary Fig. 11b

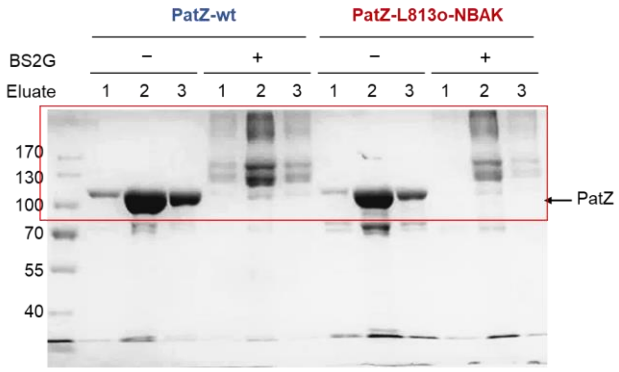

Supplementary Fig. 20a

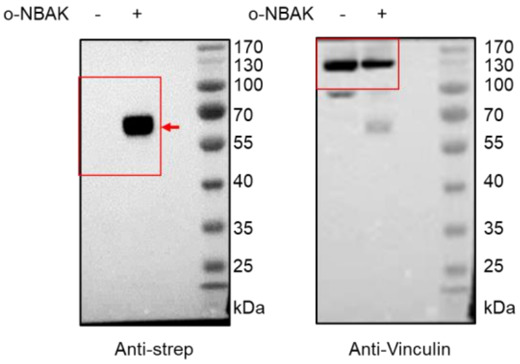

Supplementary Fig. 21a

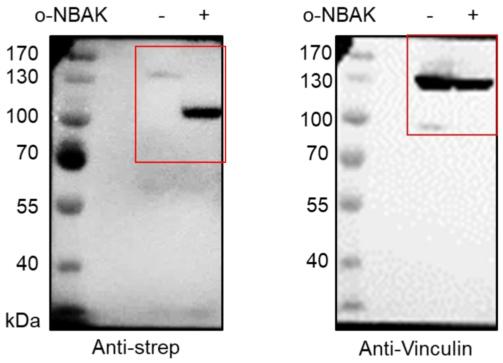

Supplement: Supplementary file 1 — Supplementary Information [file 41467_2024_45765_MOESM1_ESM.pdf]
