# Supplementary material for: Spatiotemporal and direct capturing global substrates of lysine-modifying enzymes in living cells
Source: Nat Commun. 2024 Feb 17;15:1465. doi: 10.1038/s41467-024-45765-3 (PMC10874396; doi:10.1038/s41467-024-45765-3)
Supplement: Supplementary file 2 — Reporting Summary [file 41467_2024_45765_MOESM2_ESM.pdf]

nature portfolio

Corresponding author(s): Xiao-Hua Chen; Minjia Tan

Last updated by author(s): Jan 18, 2024

Reporting Summary

Nature Portfolio wishes to improve the reproducibility of the work that we publish. This form provides structure for consistency and transparency in reporting. For further information on Nature Portfolio policies, see our Editorial Policies and the Editorial Policy Checklist.

Statistics

For all statistical analyses, confirm that the following items are present in the figure legend, table legend, main text, or Methods section.

n/a	Confirmed	
☐	☒	The exact sample size (*n*) for each experimental group/condition, given as a discrete number and unit of measurement
☐	☒	A statement on whether measurements were taken from distinct samples or whether the same sample was measured repeatedly
☐	☒	The statistical test(s) used AND whether they are one- or two-sided<br>*Only common tests should be described solely by name; describe more complex techniques in the Methods section.*
☒	☐	A description of all covariates tested
☒	☐	A description of any assumptions or corrections, such as tests of normality and adjustment for multiple comparisons
☐	☒	A full description of the statistical parameters including central tendency (e.g. means) or other basic estimates (e.g. regression coefficient) AND variation (e.g. standard deviation) or associated estimates of uncertainty (e.g. confidence intervals)
☐	☒	For null hypothesis testing, the test statistic (e.g. *F*, *t*, *r*) with confidence intervals, effect sizes, degrees of freedom and *P* value noted<br>*Give P values as exact values whenever suitable.*
☒	☐	For Bayesian analysis, information on the choice of priors and Markov chain Monte Carlo settings
☒	☐	For hierarchical and complex designs, identification of the appropriate level for tests and full reporting of outcomes
☒	☐	Estimates of effect sizes (e.g. Cohen's *d*, Pearson's *r*), indicating how they were calculated

Our web collection on statistics for biologists contains articles on many of the points above.

Software and code

Policy information about availability of computer code

Data collection	XCalibur (3.0); Image Quant LAS 4000; ChemiScope 6300
Data analysis	MaxQuant (v. 1.6.6.0). Merox (v 2.0). Mascot (v2.3). pLabel (v2.4). Mod Web server (v r271) . GRAMM-X Protein-Protein Docking Web Server (v.1.2.0). Discover Studio 3.5 client.

For manuscripts utilizing custom algorithms or software that are central to the research but not yet described in published literature, software must be made available to editors and reviewers. We strongly encourage code deposition in a community repository (e.g. GitHub). See the Nature Portfolio guidelines for submitting code & software for further information.

Data

Policy information about availability of data

All manuscripts must include a data availability statement. This statement should provide the following information, where applicable:

- Accession codes, unique identifiers, or web links for publicly available datasets
- A description of any restrictions on data availability
- For clinical datasets or third party data, please ensure that the statement adheres to our policy

All the mass spectrometry proteomics raw data and the database search result files have been deposited to ProteomeXchange consortium via the iProX partner repository under the dataset identifier PXD040318 (URL: https://www.iprox.cn/page/PSV023.html;?url=1676947743782NxiS, Password: Icgf). The GO-BP enrichment analysis was performed with PANTHER database (v16). Protein structures were retrieved from PBD: SlPatA (PDB ID: 4NXY), YjaB (PDB ID: 2KCW), LplA (PDB ID: 3A7A), PhoP (PDB ID: 2PKX; 2PL1), NarL (PDB ID: 1RNL), TmcA (PDB ID: 2ZPA), GCN5 (PDB ID: 1Z4R), Tip60 (PDB ID: 2OU2). The processed proteomics data

and the GO-BP enrichment analysis data are available in Supplementary Data file. Other data generated in this study are provided in the Supplementary Information/Source Data file.

Research involving human participants, their data, or biological material

Policy information about studies with human participants or human data. See also policy information about sex, gender (identity/presentation), and sexual orientation and race, ethnicity and racism.

Reporting on sex and gender	N/A
Reporting on race, ethnicity, or other socially relevant groupings	N/A
Population characteristics	N/A
Recruitment	N/A
Ethics oversight	N/A

Note that full information on the approval of the study protocol must also be provided in the manuscript.

Field-specific reporting

Please select the one below that is the best fit for your research. If you are not sure, read the appropriate sections before making your selection.

☒ Life sciences ☐ Behavioural & social sciences ☐ Ecological, evolutionary & environmental sciences

For a reference copy of the document with all sections, see nature.com/documents/nr-reporting-summary-flat.pdf

Life sciences study design

All studies must disclose on these points even when the disclosure is negative.

Sample size	No sample-size calculation was performed. For proteomic MS experiments, at least two biological replicates were performed to catculate the statistical significance. These sample sizes were commonly seen as in other proteomics studies.
Data exclusions	No data were excluded from the analysis.
Replication	The reproducibility of our findings was verified by repeating the experiments. All attempts to reproduce the data were successful
Randomization	For studies using cells or biochemical samples (e.g. purified proteins), samples were randomly allocated into experimental or control groups.
Blinding	Blinding is not relevant to this study because all comparisons were performed biochemical or cellular samples, with no animal or human subjects.

Reporting for specific materials, systems and methods

We require information from authors about some types of materials, experimental systems and methods used in many studies. Here, indicate whether each material, system or method listed is relevant to your study. If you are not sure if a list item applies to your research, read the appropriate section before selecting a response.

Materials & experimental systems

n/a	Involved in the study	
☐	☒	Antibodies
☐	☒	Eukaryotic cell lines
☒	☐	Palaeontology and archaeology
☒	☐	Animals and other organisms
☒	☐	Clinical data
☒	☐	Dual use research of concern
☒	☐	Plants

Methods

n/a	Involved in the study	
☒	☐	ChIP-seq
☒	☐	Flow cytometry
☒	☐	MRI-based neuroimaging

Antibodies

Antibodies used	Mouse anti-His tag monoclonal antibodies (Proteintech, Cat. No. HRP-66005, 1B7G5, 1:10000)<br>Mouse anti-vinculin monoclonal antibody (Proteintech, Cat. No. 66305-1-Ig, 2B5A7, 1:2000)

	HRP-conjugated goat anti-mouse antibody (Proteintech, Cat. No. SA00001-1, Polyclonal, 1:4000)<br>Acetylated-lysine mouse monoclonal antibody (Cell Signaling Technology, Cat. No. 9681, Ac-K-103, 1:1000)<br>HRP-conjugated goat anti-rabbit antibody (Cell Signaling Technology, Cat. No. 7074, 1:5000)<br>Rabbit polyclonal FtsZ antibody (CUSABIO, Cat. No. CSB-PA359270HA01EGX, Polyclonal, 1:1000)<br>Mouse Anti-Strep-Tag II Monoclonal Antibody (Abbkine, Cat. No. ABT2230, 8C12, 1:2000)
Validation	Commenrcial antibodies have been validated by the manufacturers. Detailed information for validation of primary antibodies are available form the websites:<br>Mouse anti-His tag monoclonal antibodies -https://www.ptgcn.com/products/6-His-His-Tag-Antibody-HRP-66005.htm<br>Mouse anti-vinculin monoclonal antibody -https://www.ptgcn.com/products/Vinculin-Antibody-66305-1-Ig.htm<br>Acetylated-lysine mouse monoclonal antibody -https://www.cellsignal.cn/product/productDetail.jsp?productId=9681<br>HRP-conjugated goat anti-rabbit antibody -https://www.cusabio.cn/Polyclonal-Antibodies/ftsZ-Antibody-1174745.html<br>Mouse Anti-Strep-Tag II Monoclonal Antibody -https://www.abbkine.cn/product/abt2230/

Eukaryotic cell lines

Policy information about cell lines and Sex and Gender in Research

Cell line source(s)	293F cells were purchased from ThermoFisher Scientific.
Authentication	Cell lines were not additionally authenticated in our hands.
Mycoplasma contamination	The cell lines were not tested for mycoplasma contamination.
Commonly misidentified lines (See ICLAC register)	No commonly misidentified cell lines were used in the study.

Plants

Seed stocks	N/A
Novel plant genotypes	N/A
Authentication	N/A
