# Supplementary material for: Spatiotemporal and direct capturing global substrates of lysine-modifying enzymes in living cells
Source: Nat Commun. 2024 Feb 17;15:1465. doi: 10.1038/s41467-024-45765-3 (PMC10874396; doi:10.1038/s41467-024-45765-3)
Supplement: Supplementary file 10 — Description of Additional Supplementary Files [file 41467_2024_45765_MOESM10_ESM.pdf]

Title: Supplementary Data 1.
Description: Substrate profiling by PatZ-L813o-NBAK

Title: Supplementary Data 2.
Description: Substrate profiling by YiaC-F75o-NBAK

Title: Supplementary Data 3.
Description: Substrate profiling by LplA-A138o-NBAK and GOBP analysis of candidate substrates

Title: Supplementary Data 4.
Description: Substrate profiling by TmcA-R457o-NBAK

Title: Supplementary Data 5.
Description: Substrate profiling by YjaB-F77o-NBAK

Title: Supplementary Data 6.
Description: Substrate profiling by Tip60-C369o-NBAK Supplementary Data 7. Substrate profiling by GCN5-E575o-NBAK
